# Light-Driven Organocatalytic Birch Reduction for Late-Stage Drug Modification and sp^3^‑Rich Spirocycle Synthesis

**DOI:** 10.1021/jacs.5c17373

**Published:** 2026-01-27

**Authors:** Florian Schiel, Luca di Martile, Roberta Coccia, Andrea Palone, Magdalena Medrzycka, Laura Kqiku, Paulo Neves, Nunzio Matera, Antonio Misale, Paolo Melchiorre

**Affiliations:** † Department of Industrial Chemistry ‘Toso Montanari’, 9296 University of Bologna, via Piero Gobetti 85, Bologna 40129, Italy; ‡ Institute of Chemical Research of Catalonia, 202569 ICIQ, Av. Països Catalans 16, Tarragona 43007, Spain; § Johnson & Johnson, In-Silico Discovery (ISD), Lisbon 2740-244, Portugal; ∥ Global Discovery Chemistry, Johnson & Johnson, Calle Rio Jarama 75, Toledo 45007, Spain

## Abstract

The Birch reduction is a cornerstone transformation in synthetic chemistry, yet its widespread application is limited by harsh conditions, poor functional group tolerance, and safety concerns. Recent photochemical strategies have offered milder alternatives but have largely been restricted to polycyclic arenes (*E*_red_ ∼ −2.6 V vs SCE) and poorly suited for complex molecules of pharmaceutical relevance. Here, we report a practical organocatalytic system that enables Birch-type reductions of electron-rich benzenes (*E*_red_ = <−3.4 V vs SCE) and heteroarenes under light irradiation and mild conditions. The method is based on an indoline-2-thione anion catalyst developed from our previously reported organic super-reducing photocatalysts. The protocol exhibits a broad substrate scope, including the late-stage functionalization of drug molecules bearing sensitive functional groups. Moreover, the synthetic utility of 1,4-dihydro products bearing an alcohol side chain was showcased through a two-step telescoped Birch reduction/spirocyclization sequence, granting streamlined access to sp^3^-rich spirocyclic scaffolds, a valuable motif in medicinal chemistry. Demonstrated scalability in both batch and continuous-flow operation further underscores the translational potential of this system. This collaborative work with colleagues at *Johnson & Johnson* highlights the power of light-driven organocatalysis to deliver industrially relevant methods for the synthesis of complex drug-like molecules.

## Graphical abstract

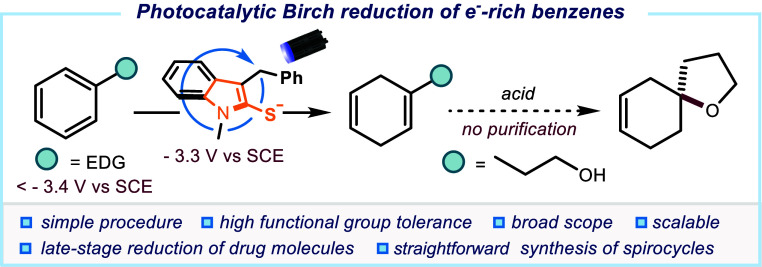

## Introduction

The Birch reduction^1^ remains one of the most powerful dearomatization strategies for converting abundant arenes into versatile 1,4-cyclohexadiene building blocks (Figure 1a).^2^ These intermediates have been extensively used in natural product synthesis and as entry points to a three-dimensional sp^3^-rich chemical space.^3^ However, the classical protocol, relying on alkali metals in liquid ammonia,^1^ suffers from significant drawbacks, including extreme reaction conditions, severe safety concerns, and poor tolerance of functional groups that preclude broad adoption in pharmaceutical settings. These limitations have motivated intense efforts to redesign the transformation under milder conditions,^4^ using electrochemical^5^ or mechanochemical^6^ strategies.

**Figure 1:**
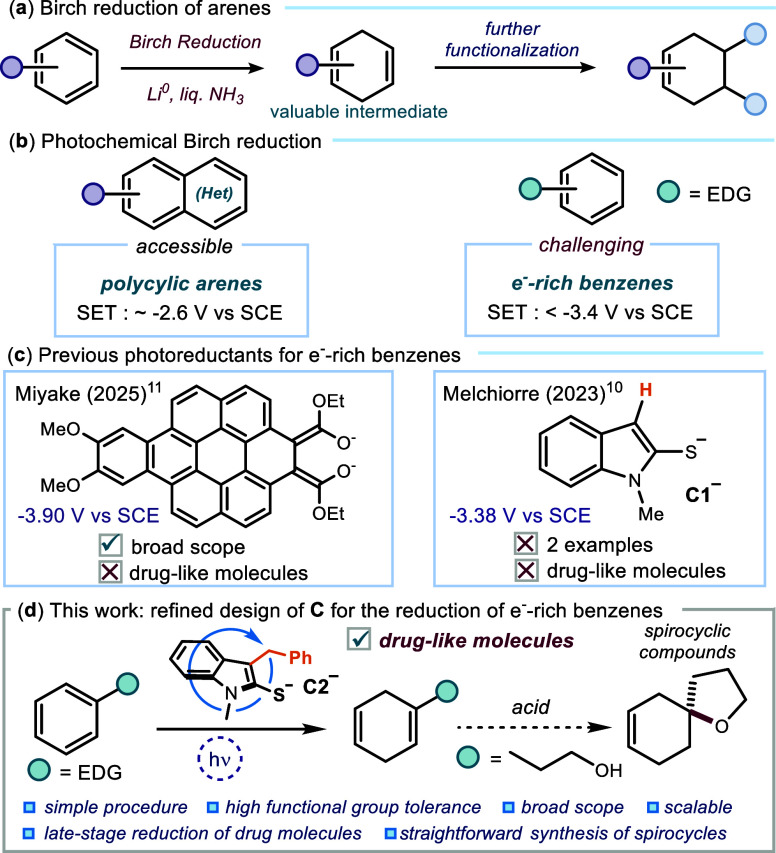
Birch reduction: (a) a classic dearomatization strategy converting arenes into 1,4-cyclohexadienes and (b) the state-of-the-art approach highlighting both easily and challenging-to-activate substrates. (c) The previous photocatalytic systems and (d) our newly developed class of organic photocatalysts for the reduction of electron-rich benzene derivatives.

Photochemical approaches are especially appealing, as they can harness light to generate highly reducing species under operationally simple and practical conditions.^7^ While recent advances have shown promise, their scope has been largely confined to polycyclic (hetero)­arenes or selected naphthalenes (*E*_red_ ∼ −2.6 V vs SCE, Figure 1b).^8^ Application to electron-rich benzenes (*E*_red_ = < −3.4 V vs SCE) and complex substrates has only been realized with systems that achieve modest yields or require prolonged reaction times.^9,10^

Recently, a relevant contribution by Miyake and co-workers^11^ offered a useful light-driven organocatalytic platform for Birch reductions, pushing the boundaries of what organic anionic photocatalysts can achieve (Figure 1c). Yet, despite these advances, general applicability to drug-like molecules remains difficult: the reported systems^8–11^ typically lack tolerance toward multiple functional groups and have not been exploited in the context of late-stage drug modification. Here, we report a photocatalytic system based on an indoline-2-thione anion **C** catalyst that overcomes these limitations (Figure 1d). Building on our previous discovery of strongly reducing organic anion **C1**,^10^ we have developed a catalyst capable of reducing electron-rich arenes and heteroarenes under mild conditions with broad tolerance of multiple, sensitive functional groups. Conducted in collaboration with colleagues at *Johnson & Johnson*, this work was designed to address real-case challenges in drug discovery, including late-stage functionalization of complex drug molecules and streamlined access to sp^3^-rich scaffolds. Moreover, 1,4-dihydro products bearing an alcohol side chain were converted through a two-step telescoped Birch reduction/cyclization sequence into spirocyclic scaffolds, a valuable motif in drug discovery. Preliminary scalability experiments in both batch and flow conditions underscore the practicality of the protocol.

## Results and Discussion

### Background and Photocatalyst Design

Our design strategy was inspired by our recent discovery^10^ that indoline-2-thione catalysts **C** can operate as strongly reducing organic anions^12^ upon light excitation. In particular, deprotonation of 1-methylindoline-2-thione (**C1**) generates a thiolate intermediate **C1** ^**–**^ capable of reaching an excited state with a reduction potential of about −3.3 V vs SCE. This strong photoreductant was shown to promote activation of otherwise inert C­(sp^2^)–X bonds via single-electron transfer (SET) pathways. In proof-of-concept studies, catalyst **C1** also mediated the Birch reduction of benzene and anisole. However, the modest efficiencies obtained in those early trials^10^ (yield of 32% and 38%, respectively) and the inability to reduce other electron-rich benzene derivatives underscored the need for a better photocatalyst. Considering the high intrinsic reducing power of this catalytic platform, we hypothesized that structural modification of the indoline-2-thione framework **C**, together with proper reaction condition optimization, could deliver a more robust and general system for the Birch reduction of electron-rich arenes and, ultimately, complex drug-like molecules.

### Developing a Strongly Reducing Photoredox Catalyst

To evaluate the potential of our catalytic platform for Birch reductions, we selected anisole **1a** as a benchmark substrate, given its relevance as a representative electron-rich arene. Using the original catalyst **C1** (5 mol %) in the presence of K_3_PO_4_ as base, *N,N*′-dimethylethylenediamine (DMEDA) as the hydrogen atom donor, MeOH as a proton source, and DMF as a solvent, and under 390 nm irradiation at 30 °C, the desired 1,4-dihydroanisole **2a** was obtained in 45% yield after 15 h (Figure 2a). Although this yield was higher than in our previous studies,^10^ primarily due to the altered reaction conditions,^14^ these results indicated that improved catalyst design was required to achieve practical efficiency. Systematic modification at the α-position of the thioamide revealed key structure–activity relationships. Introduction of a benzyl substituent (**C2**) enhanced the yield to 64%, whereas the fully methylated analogue **C3** was inactive, consistent with its inability to undergo deprotonation and form the catalytically active anion **C3** ^**–**^. Variations of the benzyl substituent showed that electron-donating groups had a minimal impact on activity (**C4**, *p*-OMe, 65% yield), whereas the electron-withdrawing *p*-CN group completely inhibited catalysis (catalyst **C5**). Replacement with saturated or aliphatic groups (**C6**–**C7**) preserved activity (59–65% yield), indicating that an aryl substituent at this position is not essential for catalysis. Extensive catalyst screening, together with the optimization of additional reaction parameters, is provided in the Supporting Information.

**Figure 2:**
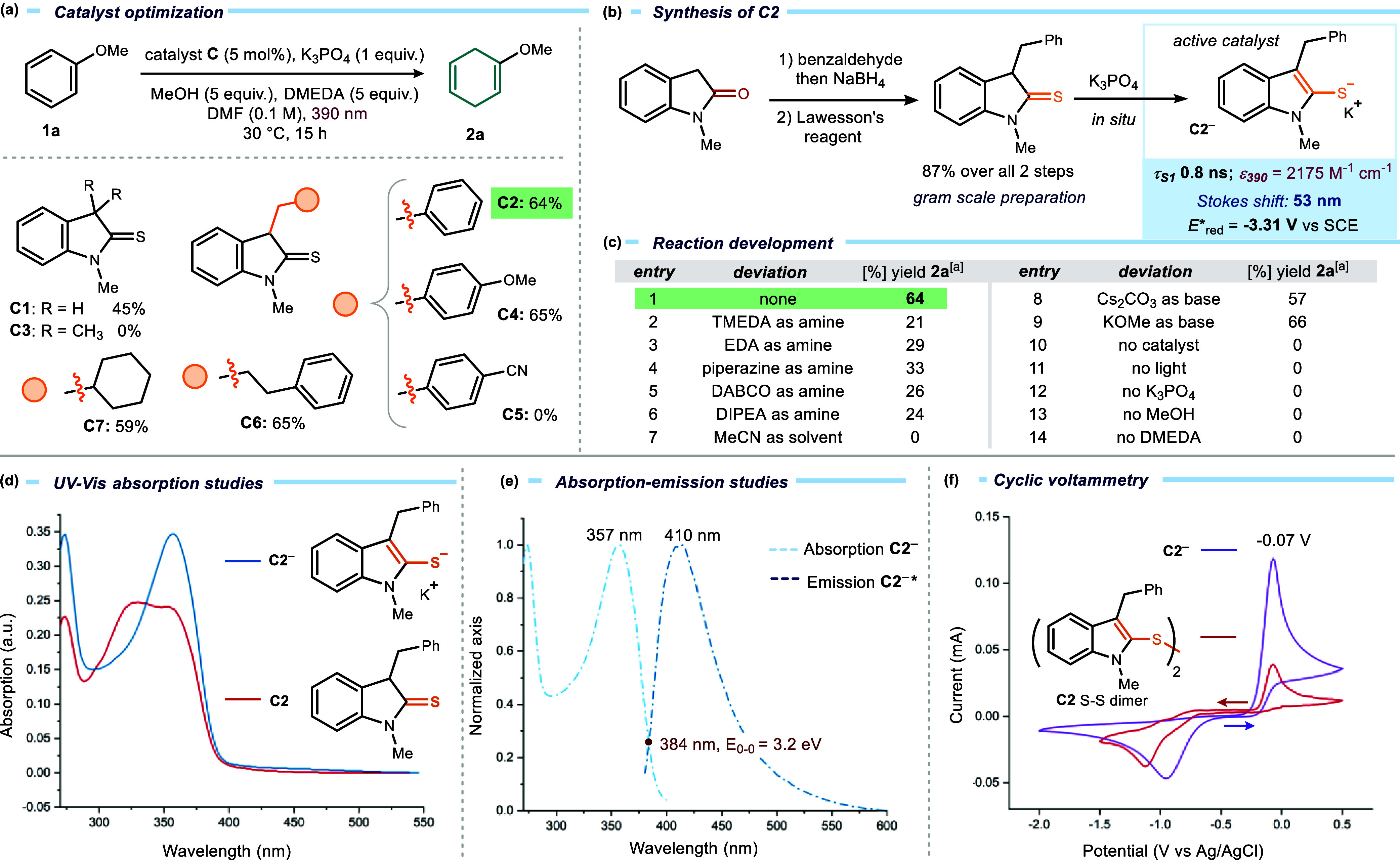
(a) Catalysts **C** development and application; model reaction with anisole **1a** performed on a 0.1 mmol scale in DMF under irradiation by purple LEDs (Kessil lamps) at 390 nm using a 3D-printed, temperature-controlled photoreactor.^13^ (b) Synthesis of catalyst **C2** and photophysical properties of the catalytic active anion **C2^–^**; τ_S1_ = singlet excited-state lifetime; ε_390_ = molar extinction coefficient at 390 nm (0.2 mM); Stokes shift = absorption–emission maxima difference; E*_red_ = excited-state reduction potential. (c) Optimization studies and control experiments. (d) UV–vis absorption spectra of catalyst **C2** and anion **C2** ^**–**^ (formed in situ treating **C2** with 2 equiv of K_3_PO_4_) in DMF. (e) Emission spectra of the excited anion **C2** ^–*^ in DMF (formed in situ treating **C2** with K_3_PO_4_) upon irradiation at 355 nm and its intercept at 384 nm with the normalized absorption spectrum, with a 0–0 transition energy (*E*_0‑0_) of 3.2 eV. (f) Cyclic voltammetry measurements of the deprotonated catalyst **C2** ^**–**^ (purple line) and the **C2** S–S dimer (red line) carried out in DMF vs Ag/AgCl at a scan rate of 100 mV/s. ^a^Yield of **2a** determined by ^1^H NMR analysis using mesitylene as the internal standard.

The improved activity of the new-generation catalysts such as **C2**, relative to progenitor **C1**, was attributed to enhanced chemical stability. Catalyst stability studies conducted under aerobic conditions—both in the dark and under light irradiation—revealed that **C1** underwent degradation, whereas **C2** remained significantly more stable (see Section F2 of the Supporting Information for details). These results established **C2** and related derivatives as a new generation of indoline-2-thione catalysts with the robustness required for broad application in Birch-type reductions.

Catalyst **C2** was selected for further studies owing to its performance, ease of synthesis, and cost-effectiveness. **C2** is an air-stable solid that can be prepared on a gram scale in two steps from inexpensive, commercially available *N*-methyl oxindole and benzaldehyde, proceeding in 87% overall yield (Figure 2b). This straightforward and scalable synthesis ensures broad accessibility of the catalyst. We next examined the role of additives (Figure 2c). DMEDA proved essential, providing the highest yield (entry 1), whereas its close analogues tetramethylethylenediamine (TMEDA) and ethylenediamine (EDA) gave significantly diminished efficiencies (21% and 29% yield, entries 2–3). Other amines, including piperazine, DABCO, and DIPEA, were also less effective (24–33% yield, entries 4–6). Solvent screening highlighted the necessity of amide-based media: DMF was optimal, while CH_3_CN was incompatible (entry 7). The inorganic base could be varied to some extent, with Cs_2_CO_3_ and KOMe providing 57% and 66% yield, respectively (entries 8–9). Finally, control experiments confirmed that each component—catalyst, light, DMEDA, MeOH, and inorganic base—was essential for reactivity (entries 10–14).

### Characterization of Photocatalyst C2

Treatment of **C2** with K_3_PO_4_ (2 equiv) in deuterated DMF (DMF-*d*7) rapidly and quantitatively generated the corresponding anion **C2** ^**–**^, as confirmed by ^1^H NMR analysis (see Section F1 in the Supporting Information). UV–Vis absorption spectroscopy showed that neutral **C2** absorbs predominantly in the near UV region, while deprotonation to give the anion **C2** ^**–**^ induces a small but detectable bathochromic shift, extending absorption until ∼390 nm, the excitation wavelength used in this study (Figure 2d), with a significant extinction coefficient (ε_390_ = 2175 M^–1^ cm^–1^ at 0.2 mM). The thiolate **C2** ^**–**^ exhibited an absorption maximum at 357 nm, while its emission spectrum after excitation at 355 nm showed a band centered at 410 nm (Figure 2e), confirming that the anion could access an electronically excited state. From the overlap of normalized absorption and emission spectra (λ = 384 nm), the 0–0 transition energy (*E*_0_,_0_) was determined to be 3.2 eV. We also established that deprotonated catalyst **C2** has a singlet excited-state lifetime of 0.8 ns (see Section F3.4 in the Supporting Information). Next, electrochemical studies using cyclic voltammetry showed that the anion **C2** ^**–**^ exhibits an irreversible oxidation peak at −0.07 V vs Ag/AgCl in DMF, corresponding to oxidation of the thiolate to a sulfur-centered radical (Figure 2f, purple line). This radical then rapidly dimerizes to form the disulfide (**C2** S–S dimer), which gives rise to a second irreversible reduction peak at 0.95 V. The assignment of this second peak to the S–S dimer was confirmed by recording the voltammogram of an authentic sample of the disulfide dimer, which showed a peak at the same potential (Figure 2f, red line). Using the Rehm–Weller formalism,^15^ the excited-state reduction potential of **C2** ^**–**^ was calculated as *E*(**C2** ^–*^/**C2** ^**•**^) = – 3.30 V vs Ag/AgCl in DMF (−3.31 V vs SCE in MeCN; see Section F5 of the Supporting Information), a value close to the reduction potential of benzene (−3.42 V vs SCE).^16^ This renders the SET reduction of benzene and related substrates thermodynamically feasible. Finally, Stern–Volmer quenching experiments demonstrated that the fluorescence of excited **C2** ^**–**^ was efficiently quenched by anisole **1a** (*K*_SV_ = 4.13 M^–1^; *k*_q_ = 5.16 × 10^9^ M^–1^ s^–1^; Section F3.3 in the Supporting Information), corroborating the ability of this photocatalyst to engage in productive SET with electron-rich arenes upon excitation.

### Establishing the Scope of Birch Reductions

With the optimized conditions in hand (Figure 2c, entry 1), we next explored the substrate scope of the Birch reduction (Figure 3, upper panel). The method proved broadly applicable to simple arenes, such as anisole, benzene, toluene, and indane, which delivered the corresponding 1,4-dihydro products **2a**–**2d** in 52–64% yield. Importantly, the protocol showed tolerance toward functional groups that are commonly encountered in drug discovery molecules and are often incompatible with classical Birch conditions. Benzene derivatives bearing free alcohols or an aliphatic amide side chain underwent smooth reduction, affording products **2e**–**2g** in good yields. Moreover, *N*-phenyl amides, a class of substrates that to the best of our knowledge has not previously been subjected to Birch reductions, were successfully reduced to **2h** and **2i** in 44 and 40% yield, respectively. Arenes containing free amines were also viable, with amino alcohol **2j** obtained in 57% yield. Importantly, benzylic amines were reduced without cleavage of the benzyl group, producing **2k** and **2l** in 52% and 39% yield, respectively. While the Birch reduction of primary benzylamines has precedents, analogous transformations of secondary or tertiary benzylic amines are, to our knowledge, unprecedented. The method also tolerated chiral enantiopure amines relevant to enantioselective organocatalysis: reduction of a representative example gave adduct **2m** in 49% yield with full stereochemical integrity at both stereocenters. Likewise, a chiral enantiopure *N*-Boc-protected aminoalcohol was smoothly converted to **2n** in 55% yield, with no loss of enantiomeric purity. Other sensitive motifs, such as carbamate side chains and acetal protecting groups, also remained untouched, giving products **2o** and **2p** in 54% and 43% yield. Finally, our catalytic platform displayed a notable chemoselectivity. Product **2q** demonstrates selective dearomatization of a benzene ring in the presence of a pyrrole heterocycle. Likewise, naphthalene derivatives bearing free alcohols were reduced efficiently, affording **2r** and **2s** in 54% and 82% yield, respectively. While the method accommodates a broad range of electron-rich and electron-neutral arenes, several limitations were identified. Besides pyridines and benzoic acids, substrates bearing strongly electron-withdrawing groups (such as nitriles, amides, esters, and CF_3_) proved unreactive, whereas substrates containing functionalities prone to competitive pathways (including epoxides and sensitive heterocycles) underwent decomposition. A full overview of low-yielding, unreactive, and decomposing substrates is provided in Figure S1 of the Supporting Information.

**Figure 3:**
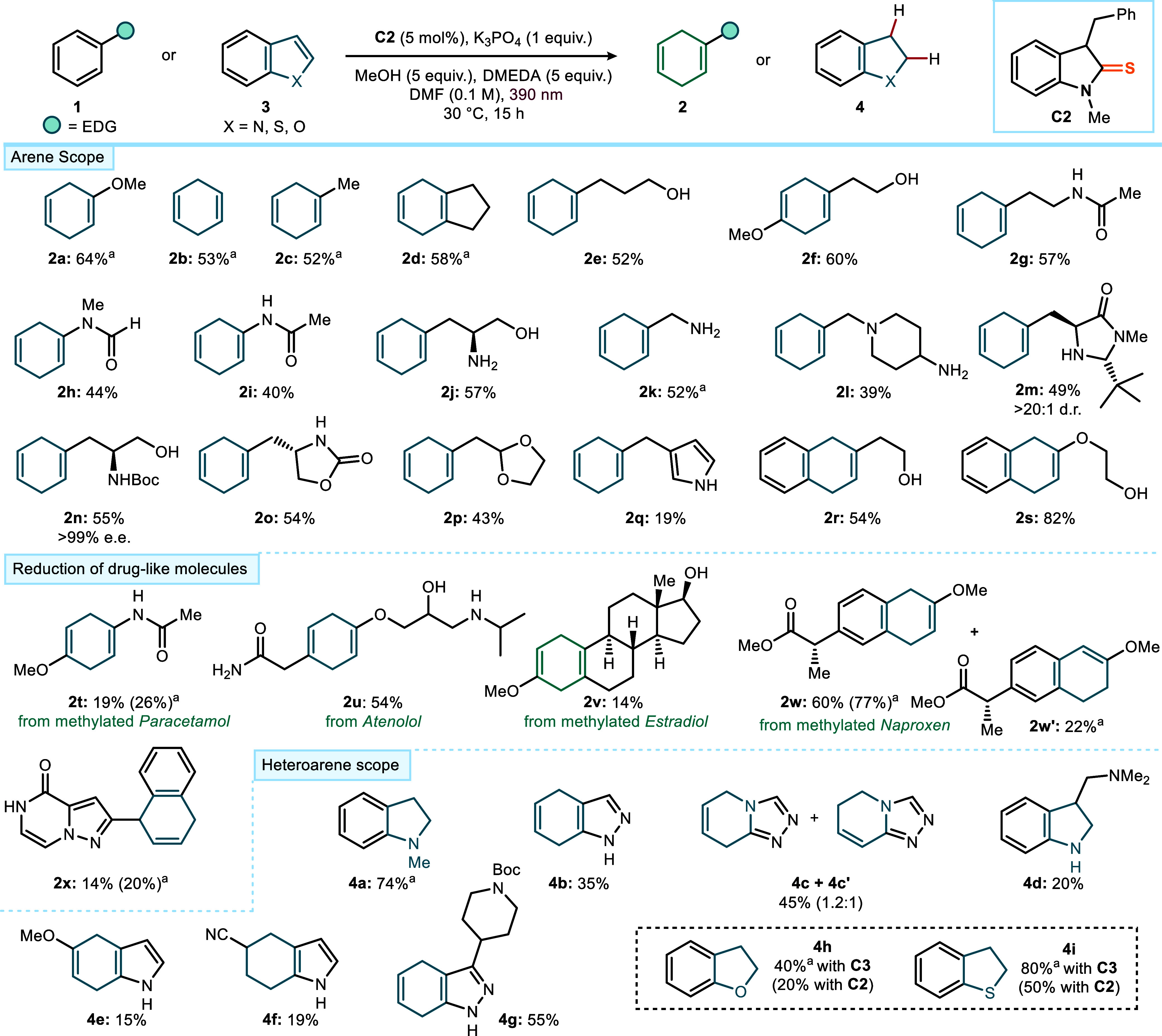
Photocatalytic Birch reduction of electron-rich arenes and heteroarenes. Reactions were performed in DMF (0.1 M) at 30 °C on a 0.2 mmol scale under illumination at 390 nm in a 3D-printed, temperature-controlled photoreactor;^13^ **C2** (5 mol%), K_3_PO_4_ (1 equiv), MeOH (5 equiv), and DMEDA (5 equiv). Yields refer to the isolated products **2** and **4** and are reported as the average of two runs. ^a^Yield measured by ^1^H NMR analysis using mesitylene as the internal standard.

### Late-Stage Functionalization of Drug Analogues

Next, we examined the potential of this highly reducing photocatalytic system in the late-stage functionalization of complex drug-like molecules (Figure 3, central panel). A *Paracetamol* derivative was successfully converted to the corresponding 1,4-dihydro product **2t** in 19% yield. The clinically used β-blocker *Atenolol*, which contains an unprotected amide and alcohol, as well as a secondary amine, underwent selective dearomatization to give **2u** in 54% yield. The ability to engage such a densely functionalized scaffold under mild conditions underscores the robustness of this method in real-life pharmaceutical settings. The trisubstituted, electron-rich *Estradiol* derivative was also activated, affording product **2v** in 14% yield. In addition, the naphthalene-containing anti-inflammatory drug *Naproxen* was reduced efficiently, delivering a mixture of regioisomeric products **2w** (60%) and **2w′** (22%). Even more challenging frameworks proved to be accessible: a naphthalene ring was reduced selectively in the presence of a pyrazolo­[1,5-*a*]­pyrazin-4­(5H)-one pharmacophore, affording product **2x** in 14% yield. These results highlight the capacity of our light-driven organocatalytic Birch reduction to operate directly on complex, drug-like scaffolds bearing multiple functional groups. Such late-stage transformations extend the utility of Birch chemistry beyond early-stage model systems and highlight its potential as a practical tool for drug discovery.

### Reduction of Heteroarenes

Heteroaromatic scaffolds are broadly encountered in pharmaceuticals and bioactive molecules, making their selective dearomatization a particularly valuable transformation. We therefore investigated the applicability of our light-driven protocol to a range of heteroarenes (Figure 3, lower panel). Using catalyst **C2**, several nitrogen-containing heteroarenes were successfully reduced. An indole derivative was converted to the corresponding product **4a** in 74% yield, while indazole afforded product **4b** in 35% yield with reduction occurring selectively on the benzene ring. Likewise, [1,2,4]­triazolo­[4,3-*a*]­pyridine underwent reduction to a mixture of adducts **4c**/**4c′** in 45% combined yield with a 1.2:1 ratio. Functionalized indoles carrying polar substituents were also viable: substrates bearing an aminoalkyl side chain or a methoxy group furnished reduced products **4d** and **4e** in 20% and 15% yield, respectively. The presence of a nitrile group as an electron-withdrawing substituent was tolerated, although it led to over-reduction to afford adduct **4f**. Finally, an indazole derivative bearing an *N*-Boc-protected piperidine moiety was transformed into **4g** in 55% yield. In contrast, oxygen- and sulfur-containing heteroarenes were more challenging under the optimized conditions with catalyst **C2**, as over-reduction competed with productive reactivity. Switching to catalyst **C3** offered better results, delivering the desired products **4h** and **4i** in 40% and 80% yield, respectively (compared to 20% and 50% with **C2**). Our mechanistic studies, detailed in Section F of the Supporting Information, show that **C3** does not operate through deprotonation but follows a distinct light-driven pathway initiated by quenching of its excited state by DMEDA, which generates a strongly reducing radical anion **C3^•^** ^**–**^.^17^ This mechanistic manifold differs fundamentally from the SET pathway operative with **C2** and highlights the versatility of our catalyst platform, which can be tuned to engage different heteroaromatic classes.

### Spirocyclization and Scale-Up

During the Birch reduction of anisole derivative **1y** bearing a pendant alcohol chain, the expected 1,4-dihydro product **2y** was obtained only in a moderate yield. However, during purification by column chromatography on silica gel, spontaneous transformation into corresponding spirocyclic scaffold **5y** was observed (Figure 4a). This serendipitous outcome suggested that intramolecular cyclization of dihydro intermediate **2** was feasible under mild acidic conditions, opening a straightforward route to sp^3^-rich spirocycles **5** directly from aromatic precursors. This outcome prompted further investigation, as compounds **5** are either not accessible through other methods or require complex synthetic routes.^18^

**Figure 4:**
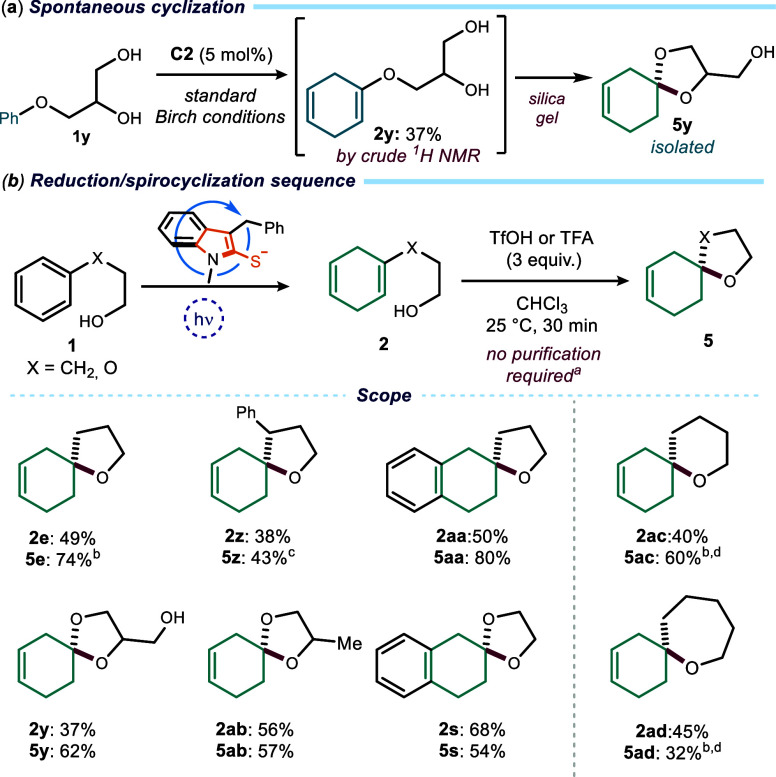
(a) Spontaneous cyclization of dihydro intermediate **2y** during silica gel purification, yielding spirocyclic scaffold **5y**. (b) Optimized two-step Birch reduction/spirocyclization sequence: treatment of crude Birch product **2** with TfOH or TFA in CHCl_3_ efficiently affords spirocycles **5** under mild conditions. Reported yields correspond to isolated **5**, calculated based on the 1,4-dihydro product **2**, while yields of **2** were determined by ^1^H NMR analysis of the crude mixture using CH_2_Br_2_ as the internal standard. TfOH was used for **5e**, **5z**, and **5aa** and TFA for **5y**, **5s**, and **5ab**. ^a^Filtration and solvent evaporation were carried out prior to the cyclization. ^b^Yield measured by ^1^H NMR analysis using CH_2_Br_2_ as the internal standard. ^c^Cyclization performed at 70 °C for 16 h. ^d^Reaction performed on the purified intermediate **2**, using 10 equiv of HCl in dioxane (4 N), 15 h, without CHCl_3_.

We therefore optimized conditions to promote spirocyclization (Figure 4b). After completion of the Birch reduction, the crude mixture was subjected to simple filtration to remove the inorganic base, followed by solvent evaporation. Subsequent addition of trifluoromethanesulfonic acid (TfOH, 3 equiv) in CHCl_3_ efficiently cyclized the crude Birch product **2**, affording the spiro-product **5**. The cyclization was complete within 30 min at room temperature. We therefore examined the generality of this Birch reduction/spirocyclization sequence. A variety of arenes proved compatible: five-membered ether derivatives furnished **5e**, **5z**, and **5aa** in good yield, while acetals such as **5y**, **5ab**, and **5s** were obtained in 54–62% yield when using the milder trifluoroacetic acid (TFA, 3 equiv). In addition, the sequence could be extended beyond five-membered rings: the six- and seven-membered spirocycles **5ac** and **5ad** were obtained in 60% and 32% yield, respectively. Collectively, these results demonstrate that the tandem Birch reduction/spirocyclization sequence offers a direct and streamlined entry to stereochemically complex sp^3^-rich spirocyclic motifs from simple, flat arene substrates.

The scalability of the photocatalytic Birch reduction was next evaluated in both batch and flow (Figure 5). In batch, substrate **1e** was reduced on a 3.5 mmol scale in the 3D-printed photoreactor, affording **2e** in 50% yield (480 mg)—comparable to the small-scale experiments. For continuous flow, optimization was required due to the poor solubility of K_3_PO_4_ in DMF. Replacement with KOMe (25% in MeOH) and addition of 18-crown-6 ether prevented base precipitation, enabling smooth operation.

**Figure 5:**
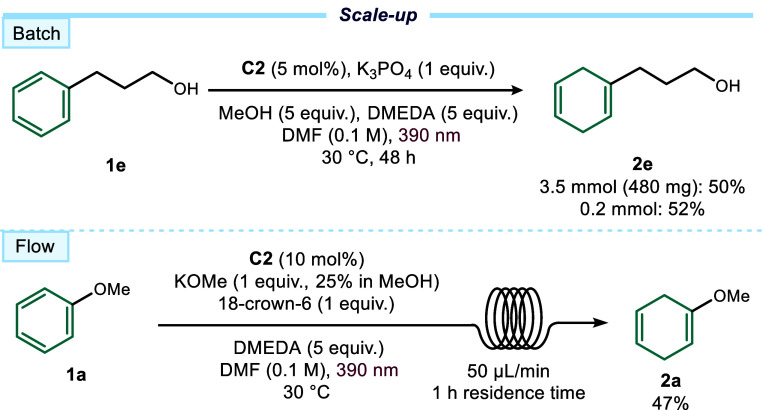
Scale-up experiments performed in batch and continuous flow. Yield of **2e** refers to the isolated product, while yield of **2a** was determined by ^1^H NMR analysis of the crude mixture using 1,3,5-triisopropylbenzene as the internal standard.

The reaction was performed in a custom 3D-printed flow unit equipped with a 6 m PFA coil (0.8 mm internal diameter) housed within the same photoreactor setup used for all other reactions in this work^13^ (see Section E5 in the Supporting Information for full details of the experimental setup). Using 10 mol% catalyst loading and a flow rate of 50 μL/min (residence time = 1 h), the reduced anisole product **2a** was isolated in 47% yield.

Together, these studies highlight the synthetic utility and operational versatility of the light-driven organocatalytic Birch reduction: beyond enabling late-stage functionalization of drug-like molecules, it can be directly coupled to spirocyclization chemistry and translated to gram-scale preparation under both batch and flow conditions.

### Mechanistic Studies

Based on photophysical characterization and control experiments (Figure 2), the catalytic cycle depicted in Figure 6a is proposed for the photoinduced Birch reduction of electron-rich arenes. Upon irradiation with 390 nm light, the deprotonated photocatalyst **C2** ^**–**^ reaches its highly reducing excited state **C2** ^**–**^ *. This excited species triggers an SET to arene **1**, generating arene radical anion **II** and sulfur-centered radical **I**. Stern–Volmer quenching studies with anisole **1a** confirmed this step (Figure 6b), and control experiments verified that the quenching is dynamic rather than static (see Figure S22 in the Supporting Information for details). The sulfur radical **I** then undergoes dimerization to form catalyst **C2** S–S dimer **III**. The formation of this intermediate is consonant with the electrochemical behavior of catalyst **C2**, as discussed in Figure 2f.

**Figure 6:**
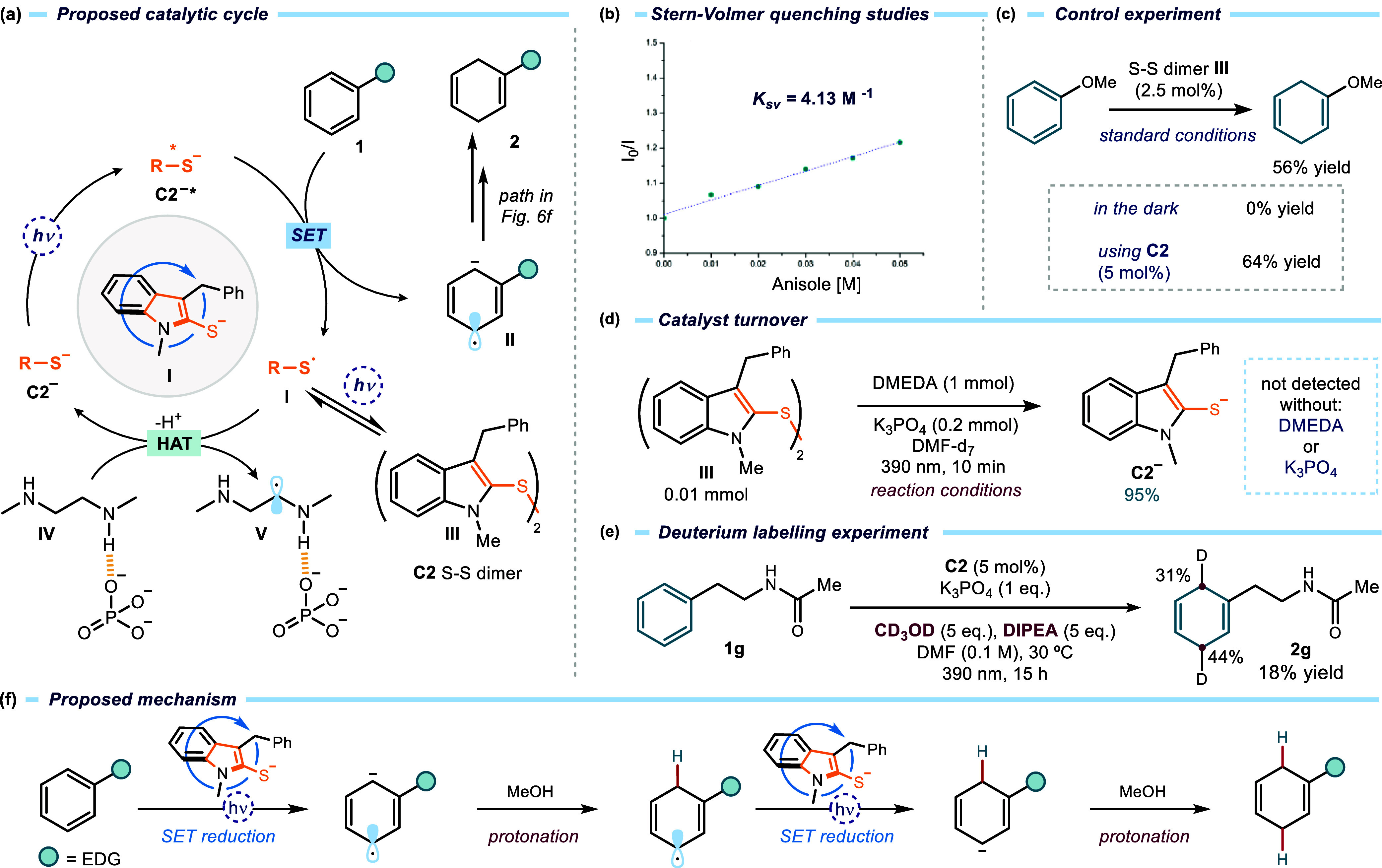
(a) Proposed catalytic cycle of the photoinduced Birch reduction of electron-rich arenes. (b) Stern–Volmer quenching studies of the excited anion **C2** ^**–**^ * in DMF (formed in situ treating **C2** with K_3_PO_4_) using anisole **1a** as a quencher. (c) Control experiments using the S–S dimer **III** as a catalyst. (d) Catalyst turnover studies by irradiating the S–S dimer **III** under the reaction conditions with and without DMEDA and K_3_PO_4_. (e) Deuterium labeling experiments using methanol-*d*_*4*_ and DIPEA (to avoid H/D exchange with the amine). (f) Proposed mechanism following two sequential SET/protonation steps.

To verify if the S–S dimer **III** could be catalytically competent, we used an authentic sample of **III** (2.5 mol%) to catalyze the model reaction. The reduced anisole product **2a** was obtained in a comparable yield as with catalyst **C2** (Figure 6c). Importantly, no reaction was observed when the same experiment was performed in the dark. These results are informative of the reaction mechanism since they imply that the S–S dimer **III** is a photoactive species which exists in a light-driven equilibrium with the progenitor sulfur radical **I**. This dimerization manifold confers a longer lifetime to **I**,^19^ thus facilitating catalyst turnover.

The turnover of oxidized catalyst **I** is proposed to occur via hydrogen-atom transfer (HAT) from DMEDA·K_3_PO_4_ adduct **IV**. Direct HAT from the α-C–H bonds of aliphatic amines (∼88–92 kcal/mol)^20^ is unlikely under standard conditions due to their high bond dissociation energy compared to thiophenols (∼75–80 kcal/mol).^21^ However, ^1^H NMR studies revealed that K_3_PO_4_ forms hydrogen-bonding interactions with DMEDA (intermediate **IV**, Figure 6a; see section F6 in the Supporting Information for details). Such interactions are known to activate α-C–H bonds in alcohols toward HAT by increasing electron density at the heteroatom,^22^ and we propose that a similar polarization effect facilitates HAT from the α-C–H of DMEDA to the sulfur radical **I**.^23^ The polarity-matched interaction between the electrophilic sulfur radical **I** and the nucleophilic α-amino C–H bond^24^ further favors this HAT kinetically. Experimental support for the proposed turnover step was obtained by irradiating S–S dimer **III** with 390 nm LEDs under standard conditions (in the presence of DMEDA and K_3_PO_4_). **III** was fully consumed, and 1.9 equiv of anion **C2** ^**–**^ was generated (Figure 6d), demonstrating that the dimer serves as a competent reservoir for catalyst **C**. This observation confirms that photolysis of S–S dimer **III** regenerates the sulfur radical **I**, which undergoes HAT from the α-C–H bond of DMEDA·K_3_PO_4_ to give the corresponding thiol (not shown); subsequent deprotonation by the base restores the active anion **C2** ^**–**^. Crucially, no signals corresponding to **C2** ^**–**^ were detected when the same experiment was repeated in the absence of either DMEDA or K_3_PO_4_ (Figure 6d and section F7 in the Supporting Information for details), establishing that both additives are essential for efficient catalyst turnover.

The role of MeOH as a proton donor was then investigated via deuterium-labeling experiments using methanol-*d*_*4*_ (Figure 6e). With DMEDA, no deuterium incorporation in product **2g** was observed, likely due to fast H/D exchange between methanol-*d*_*4*_ and the free N–H protons of DMEDA. However, when DIPEA was employed instead of DMEDA, 31–44% deuterium incorporation was observed in the 1,4-dihydro product **2g** (Figure 6e). Although DIPEA is less reactive than DMEDA as a hydrogen donor (see Figure 2c, entry 6), it prevents rapid H/D exchange with MeOH-*d*_*4*_, allowing the incorporation of deuterium into product **2g** and thus confirming the protonation step in the catalytic cycle.

Overall, the photochemically mediated reduction is proposed to proceed via the “classic” Birch-type mechanism, involving two sequential SET reduction/protonation steps (Figure 6f).^25^

## Conclusions

In summary, we have developed an organocatalytic photochemical platform for Birch reduction that operates under mild conditions. This system—based on a newly optimized indoline-2-thione anion catalyst—extends photocatalytic Birch chemistry to electron-rich arenes, heteroarenes, and densely functionalized drug-like molecules. Beyond its broad substrate scope, the method shows remarkable functional group tolerance including free alcohols, unprotected amines, amides, carbamates, Boc groups, and acetals. Furthermore, our protocol enables a Birch reduction-spirocyclization sequence, providing a new entry to medicinally relevant spirocycles and illustrating how mild Birch conditions unlock transformations inaccessible to classical protocols. Preliminary scalability experiments in batch and continuous flow underscore the practicality and potential for industrial translation. This study exemplifies how academic–industrial collaborations can accelerate the development of enabling technologies. By joining forces with colleagues at *Johnson & Johnson*, we validated the method in the context of pharmaceutically relevant molecules, highlighting its promise as a tool for late-stage diversification and drug discovery.

## Supplementary Material



## Notes

Details of experimental procedures and full characterization data and copies of NMR spectra (PDF)
