# Supplementary material for: Light-Driven Organocatalytic Birch Reduction for Late-Stage Drug Modification and sp3‑Rich Spirocycle Synthesis
Source: J Am Chem Soc. 2026 Jan 27;148(5):5202–10. doi: 10.1021/jacs.5c17373 (PMC12903859; doi:10.1021/jacs.5c17373)

Supplementary Information for

Light-Driven Organocatalytic Birch Reduction for Late-Stage Drug Modification and sp^3-Rich Spirocycle Synthesis

Florian Schiel,[†] Luca di Martile,[‡] Roberta Coccia,[‡] Andrea Palone,[‡] Magdalena Medrzycka,[‡] Laura Kqiku,[†] Paulo Neves,[§] Nunzio Matera,[‡] Antonio Misale,[±]* and Paolo Melchiorre[‡]*

[‡]University of Bologna, Department of Industrial Chemistry '*Toso Montanari*', via Piero Gobetti 85, 40129 Bologna, Italy

[†]*ICIQ*, Institute of Chemical Research of Catalonia, Av. Països Catalans 16, Tarragona, Spain

[§]*Johnson & Johnson*, In-Silico Discovery (ISD), 2740-244 Lisbon, Portugal

[±]*Johnson & Johnson*, Global Discovery Chemistry, Calle Rio Jarama 75, Toledo, Spain

*Correspondence to: amisale@ITS.JNJ.com & p.melchiorre@unibo.it

Table of Contents

A. General Information 3

B. Unsuccessful and Moderately Reactive Substrates 4

C. Synthesis of Catalysts **C** 5

D. Optimization Studies 10

E. Experimental Procedures 12

E.1. General Procedure for the Birch Reductions of Benzene Derivatives 12

E.2. General procedure for the Birch Reduction of Heteroarenes 23

E.3. Procedure for the telescoped Birch reduction/spirocyclization sequence 26

E.4. Procedure for the scale up in batch 30

E.5. Procedure for the flow-scale up 31

F. Mechanistic Studies 32

F.1. Deprotonation Experiments 32

F.2. Catalyst Stability Studies 34

F.2.1. **C1** and **C2** catalysts stability studies under aerobic conditions 34

F.2.2. Catalysts stability studies under the reaction conditions 35

F.3. Photophysical Studies 38

F.3.1. Absorption measurements 39

F.3.2. Emission Studies 40

F.3.3. Stern-Volmer Quenching Studies 41

F.3.4. Lifetime Measurements 42

F.4. Electrochemical Studies 44

F.4.1. Cyclic voltammetry measurements 45

F.4.2. Conversion of the potential from Ag/AgCl to SCE 46

F.5. Evaluation of the Excited-State Potential of the Deprotonated Catalyst **C2**$^-$ 46

F.6. Evidence of Hydrogen Bonding Interaction Between DMEDA and K_3PO_4 47

F.7. Turnover studies with **C2** S−S dimer 47

F.8. Proposed Mechanism for **C3** 48

F.9. Deuterium Labelling Experiments 51

G. References 52

A. General Information

The NMR spectra were recorded at 400, 500 or 600 MHz for ^{1}H and 101, 126 or 151 MHz for ^{13}C. The chemical shift (δ) for ^{1}H and ^{13}C are given in ppm relative to residual signals of the solvents ($CHCl_3$ at 7.26 ppm for ^{1}H NMR and 77.06 ppm for ^{13}C NMR). Coupling constants are given in Hertz. The following abbreviations are used to indicate the multiplicity: s, singlet; d, doublet; q, quartet; m, multiplet; br, broad signal.

High resolution mass spectra (HRMS) were obtained from the Mass Facility unit on a Waters Xevo Q Tof spectrometer with electrospray ionization (ESI). UV-vis measurements were carried out on a Cary 3500 Multicell UV-vis spectrophotometer. Cyclic voltammetry studies were carried out on a Autolab PGSTAT204 potentiostat offering compliance voltage up to ± 20 V (available at the counter electrode), ± 10 V scan range and ±0.4A current range. Fluorescence measurements were carried out on an Agilent Cary Eclipse fluorescence spectrometer equipped with a high voltage PMT detector and continuum Xe light source. The enantiomeric excess (ee) of the products was determined using Agilent 1100 or 1200 series HPLC instruments equipped with chiral stationary phase columns, and a UV detector operating at 210 nm. X-ray data were acquired on a Bruker APEX-2 diffractometer.

Yields refer to isolated materials of >95% purity as determined by ^{1}H NMR analysis, unless mentioned otherwise. When specified, yields were determined by ^{1}H NMR analysis of the crude mixture using an internal standard, typically in cases where the product was too volatile for isolation or difficult to separate from the starting material.

General Procedures. All reactions were set up under an argon atmosphere using standard Schlenk technique. Synthesis grade solvents were used as purchased and anhydrous solvents were taken from commercially available septa-sealed bottles. Chromatographic purification of products was accomplished using forced-flow chromatography (FC) on silica gel (230-400 mesh). For thin layer chromatography (TLC) analysis throughout this work, Merck pre-coated TLC plates (silica gel 60 GF_{254}, 0.25 mm) were employed, using UV light as the visualizing agent and an acidic mixture of vanillin or basic aqueous potassium permanganate ($KMnO_4$) stain solutions, and heat as developing agents. Organic solutions were concentrated under reduced pressure on a Büchi rotatory evaporator.

Materials. Commercial grade reagents and solvents were purchased at the highest quality from commercial suppliers and used as received, unless otherwise stated.

B. Unsuccessful and Moderately Reactive Substrates

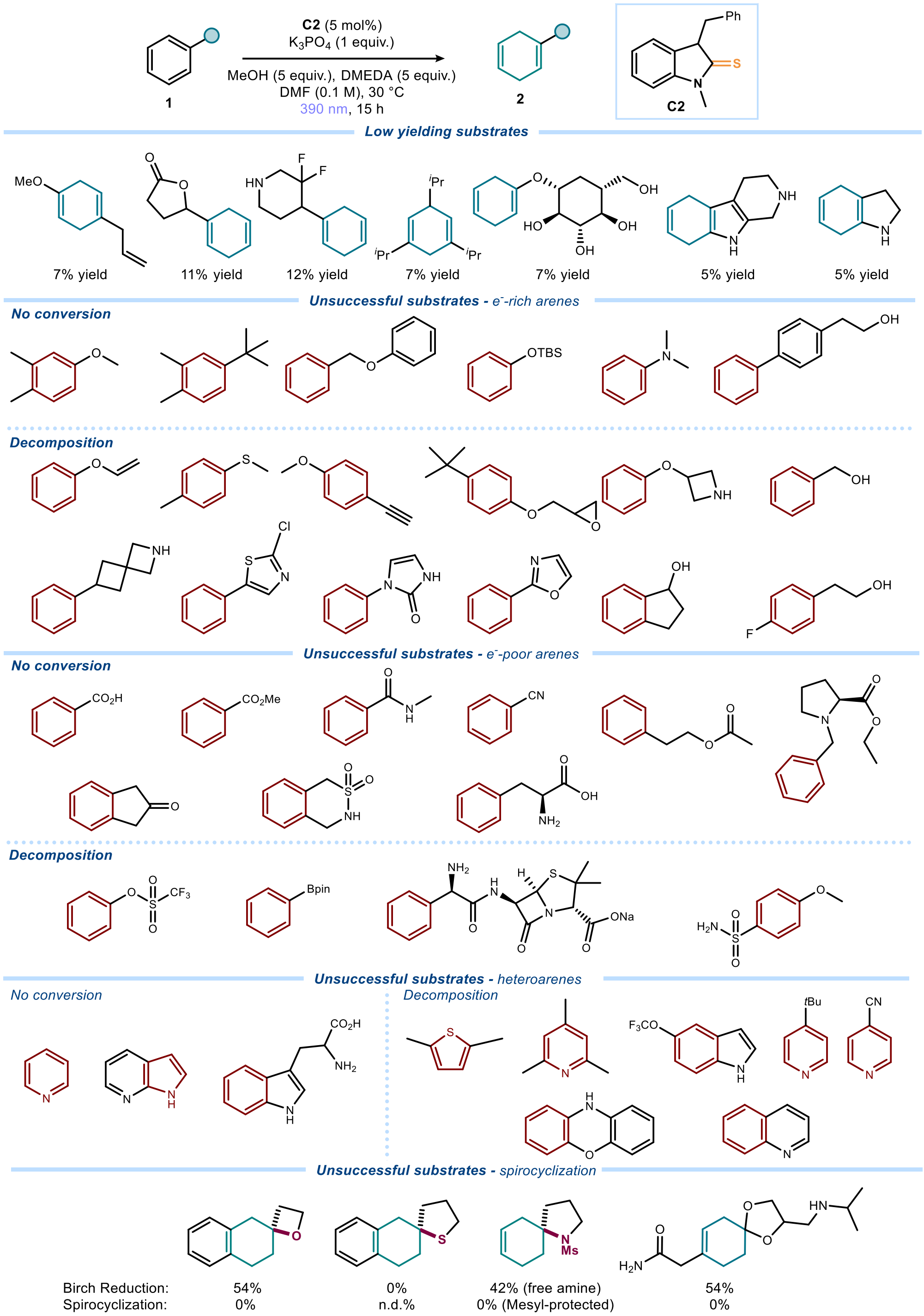

Figure S1. Unsuccessful and moderately reactive substrates. Reactions were carried out on a 0.2 mmol scale under the optimized conditions reported in the main manuscript.

C. Synthesis of Catalysts C

Synthesis of catalyst **C1:**

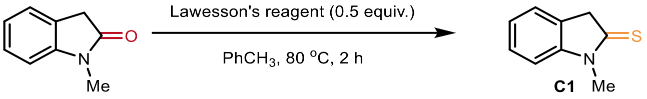

Catalyst **C1** was prepared according to our previously reported procedure.[1] A flask was charged with 1-methylindolin-2-one (736 mg, 5 mmol, 1 equiv.), the Lawesson's reagent (0.55 g, 1.36 mmol, 0.55 equiv.) and toluene (12.5 mL, 0.4 M). The mixture was purged with argon and then stirred at 80 ºC for 1 h. The solution was concentrated in vacuo and the crude mixture was purified by flash column chromatography on silica gel (hexane/EtOAc 10:1) to afford catalyst **C1** as a yellow solid (620.5 mg, 76% yield).

^{1}H NMR (400 MHz, $CDCl_3$) δ 7.37 – 7.28 (m, 2H), 7.16 (td, *J* = 7.5, 1.0 Hz, 1H), 6.97 (d, *J* = 7.9 Hz, 1H), 4.11 (s, 2H), 3.63 (s, 3H).

^{13}C NMR (101 MHz, $CDCl_3$) δ 201.3, 146.8, 129.3, 128.1, 124.5, 124.1, 109.7, 49.2, 31.4.

Characterization data matching reported literature data.[1]

Synthesis of catalysts **C3:**

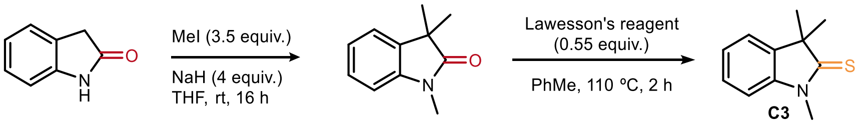

Oxindole (2 g, 15 mmol, 1 equiv.) was dissolved in THF (48 mL) and NaH (2.4 g, 60 mmol, 4 equiv., 60% dispersion in oil) was added. The solution was cooled to 0 °C and MeI (3.27 mL, 52.6 mmol, 3.5 equiv.) was added. The reaction was stirred at ambient temperature overnight and then quenched with water. The product was extracted with EtOAc, dried with $MgSO_4$ and concentrated in vacuo. The crude product was purified by flash column chromatography on silica gel (hexane/EtOAc 20:1) to afford the intermediate as colourless oil (1.43 g, 8.16 mmol, 54% yield). A flask was charged with 1,3,3-trimethylindolin-2-one (1.43 g, 8.16 mmol, 1 equiv.), the Lawesson's reagent (1.65 g, 4.08 mmol, 0.5 equiv.) and toluene (31 mL, 0.5 M). The mixture was purged with argon and then stirred at 110 ºC for 2 h. The solution was concentrated in vacuo and the crude mixture was purified by flash column chromatography on silica gel (EtOAc/hexane 10:1) to afford catalyst **C3** as a yellow oil (1.2 g, 78% yield).

^{1}H NMR (500 MHz, $CDCl_3$) δ 7.36 – 7.29 (m, 2H), 7.19 (td, *J* = 7.5, 0.9 Hz, 1H), 7.05 (d, *J* = 7.7 Hz, 1H), 3.66 (s, 3H), 1.44 (s, 6H).

^{13}C NMR (101 MHz, $CDCl_3$) δ 212.2, 144.1, 140.6, 127.9, 124.4, 122.9, 109.7, 55.1, 31.6, 28.2.

Characterization data matching reported literature data.[2]

Synthesis of catalysts **C2-C7:** (General procedure **A**)

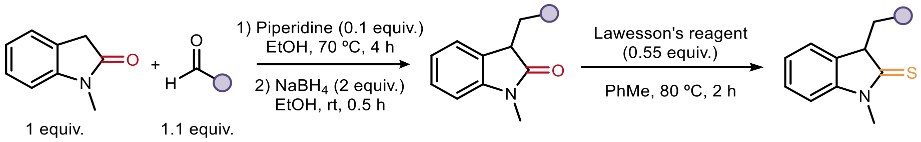

1-Methylindolin-2-one (1 equiv.), aldehyde (1.1 equiv.) and piperidine (0.1 equiv.) were dissolved in ethanol (0.3 M), then shortly purged with argon and stirred for 4 h at 70 ºC. After the solution was cooled to ambient temperature, $NaBH_4$ (2 equiv.) was added and stirred for 30 minutes. The reaction was quenched with water, extracted with EtOAc, dried with $MgSO_4$ and the solvent was removed under reduced pressure. The crude mixture was purified by flash column chromatography on silica gel (hexane/EtOAc 10:1). A flask was charged with the purified intermediate, the Lawesson's reagent (0.55 equiv.) and toluene (0.4 M). The mixture was purged with argon and then stirred at 80 ºC for 1 h. The solution was concentrated in vacuo and the crude mixture was purified by flash column chromatography on silica gel to afford the target catalyst **C**.

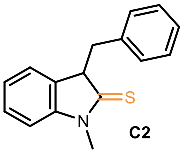

C2

3-Benzyl-1-methylindoline-2-thione (C2)

Synthesized according to the general procedure **A** using 1-methylindolin-2-one (2.2 g, 15 mmol, 1 equiv.) and benzaldehyde (1.67 mL, 16.5 mmol, 1.1 equiv.). The crude mixture was purified by flash column chromatography on silica gel (hexane/EtOAc 30:1) to afford catalyst **C2** as an orange solid (3.32 g, 87% yield over all 3 steps).

^{1}H NMR (500 MHz, $CDCl_3$) δ 7.29 – 7.21 (m, 4H), 7.20 – 7.17 (m, 2H), 7.00 (td, J = 7.5, 1.0 Hz, 1H), 6.93 (d, J = 7.9 Hz, 1H), 6.69 (dd, J = 7.4, 0.9 Hz, 1H), 4.09 (dd, J = 9.7, 4.6 Hz, 1H), 3.82 (dd, J = 13.6, 4.6 Hz, 1H), 3.60 (s, 3H), 2.86 (dd, J = 13.6, 9.7 Hz, 1H).

^{13}C NMR (126 MHz, $CDCl_3$) δ 204.9, 145.7, 138.1, 133.2, 129.7, 128.4, 128.2, 126.8, 124.8, 123.9, 109.5, 58.9, 41.0, 31.5.

HRMS (ESI$^+$) $C_{16}H_{16}NS$ [M+H]$^+$: found 254.1002, required 254.0998.

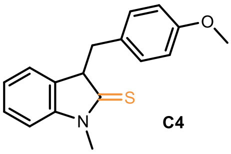

C4

3-(4-Methoxybenzyl)-1-Methylindoline-2-thione (C4)

Synthesized according to the general procedure **A** using 1-methylindolin-2-one (147 mg, 1 mmol, 1 equiv.) and *p*-anisaldehyde (134 μL, 1.1 mmol, 1.1 equiv.). The crude mixture was purified by flash column chromatography on silica gel (hexane/EtOAc 20:1) to afford catalyst **C4** as an orange solid (150 mg, 53% yield over all 3 steps).

^{1}H NMR (500 MHz, CDCl$_3$) δ 7.29 – 7.25 (m, 1H), 7.10 – 7.06 (m, 2H), 7.02 (td, *J* = 7.6, 1.0 Hz, 1H), 6.92 (d, *J* = 7.9 Hz, 1H), 6.79 – 6.74 (m, 3H), 4.05 (dd, *J* = 9.4, 4.6 Hz, 1H), 3.78 (s, 3H), 3.74 (dd, *J* = 13.7, 4.6 Hz, 1H), 3.58 (s, 3H), 2.85 (dd, *J* = 13.7, 9.4 Hz, 1H).

^{13}C NMR (126 MHz, CDCl$_3$) δ 204.9, 158.5, 145.8, 133.3, 130.7, 129.9, 128.1, 124.8, 123.9, 113.7, 109.5, 59.2, 55.3, 40.1, 31.5.

HRMS (ESI$^+$) $C_{17}H_{17}NOSNa$ [M+Na]$^+$: found 306.0933, required 306.0923.

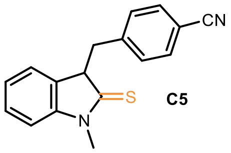
C5

4-((1-Methyl-2-thioxoindolin-3-yl)methyl)-Benzonitrile (C5)

Synthesized according to the general procedure **A** using 1-methylindolin-2-one (147 mg, 1 mmol, 1 equiv.) and 4-cyanobenzaldehyde (144 mg, 1.1 mmol, 1.1 equiv.). The crude mixture was purified by flash column chromatography on silica gel (hexane/EtOAc 20:1) to afford catalyst **C5** as a yellow solid (261 mg, 94% yield over all 3 steps).

^{1}H NMR (500 MHz, CDCl$_3$) δ 7.51 – 7.45 (m, 2H), 7.30 (t, *J* = 7.7 Hz, 1H), 7.26 – 7.21 (m, 2H), 7.07 (td, *J* = 7.5, 1.0 Hz, 1H), 6.92 (d, *J* = 7.9 Hz, 1H), 6.87 (d, *J* = 7.4 Hz, 1H), 4.12 (dd, *J* = 8.3, 4.7 Hz, 1H), 3.75 (dd, *J* = 13.7, 4.8 Hz, 1H), 3.55 (s, 3H), 3.18 (dd, *J* = 13.6, 8.2 Hz, 1H).

^{13}C NMR (126 MHz, CDCl$_3$) δ 203.9, 145.8, 143.3, 132.3, 132.0, 130.5, 128.7, 124.3, 124.2, 119.0, 110.8, 109.8, 58.3, 40.4, 31.5.

HRMS (ESI$^+$) $C_{17}H_{14}N_2SNa$ [M+Na]$^+$: found 301.0767, required 301.0770.

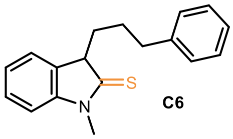
C6

1-Methyl-3-(3-phenylpropyl)indoline-2-thione (C6)

Synthesized according to the general procedure **A** using 1-methylindolin-2-one (147 mg, 1 mmol, 1 equiv.) and 3-phenylpropionaldehyde (146 µL, 1.1 mmol, 1.1 equiv.). The crude mixture was purified by flash column chromatography on silica gel (hexane/EtOAc 20:1) to afford catalyst **C6** as a yellow solid (123 mg, 44% yield over all 3 steps).

^{1}H NMR (500 MHz, CDCl$_3$) δ 7.35 (t, *J* = 7.8 Hz, 1H), 7.30 (d, *J* = 7.6 Hz, 1H), 7.28 – 7.24 (m, 2H), 7.21 – 7.16 (m, 2H), 7.15 – 7.11 (m, 2H), 7.00 (d, *J* = 7.9 Hz, 1H), 3.88 (t, *J* = 5.7 Hz, 1H), 3.65 (s, 3H), 2.69 – 2.56 (m, 2H), 2.37 – 2.26 (m, 1H), 2.26 – 2.16 (m, 1H), 1.70 – 1.58 (m, 1H), 1.44 – 1.34 (m, 1H).

^{13}C NMR (126 MHz, CDCl$_3$) δ 205.9, 146.0, 142.1, 133.7, 128.5, 128.4, 128.2, 125.9, 124.3, 123.9, 109.6, 57.6, 36.1, 33.8, 31.5, 27.0.

HRMS (ESI$^+$) $C_{18}H_{20}NS$ [M+H]$^+$: found 282.1318, required 282.1311.

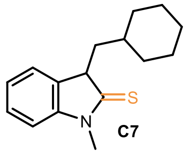

3-(Cyclohexylmethyl)-1-Methylindoline-2-thione (C7)

Synthesized according to the general procedure **A** using 1-methylindolin-2-one (147 mg, 1 mmol, 1 equiv.) and cyclohexanecarboxaldehyde (133 μL, 1.1 mmol, 1.1 equiv.). The crude mixture was purified by flash column chromatography on silica gel (hexane/EtOAc 20:1) to afford catalyst **C7** as a yellow solid (165 mg, 64% yield over all 3 steps).

^{1}H NMR (500 MHz, CDCl$_3$) δ 7.37 – 7.30 (m, 2H), 7.17 (td, *J* = 7.5, 1.0 Hz, 1H), 7.01 (d, *J* = 7.9 Hz, 1H), 3.85 (dd, *J* = 9.0, 4.9 Hz, 1H), 3.64 (s, 3H), 2.19 (ddd, *J* = 13.9, 9.2, 4.8 Hz, 1H), 1.93 – 1.85 (m, 1H), 1.80 – 1.57 (m, 6H), 1.33 – 1.14 (m, 3H), 1.11 – 0.97 (m, 2H).

^{13}C NMR (126 MHz, CDCl$_3$) δ 207.2, 145.7, 134.6, 128.0, 124.3, 124.1, 109.7, 55.4, 42.8, 35.1, 34.3, 32.5, 31.6, 26.6, 26.4, 26.3.

HRMS (ESI$^+$) $C_{16}H_{22}NS$ [M+H]$^+$: found 260.1473, required 260.1467.

Synthesis of an authentic sample of the **C2 S-S Dimer:**

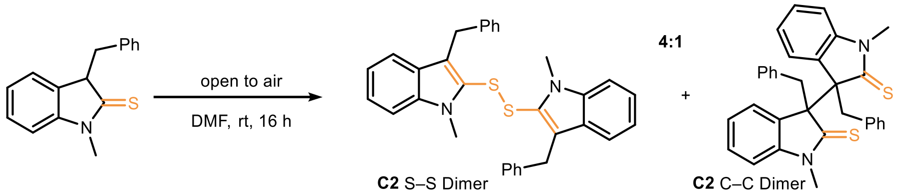

In a 50 ml flask, 3-benzyl-1-methylindoline-2-thione (50 mg, 0.2 mmol, 1 equiv.) was dissolved in DMF (3 mL) and the solution was stirred at ambient temperature overnight (open to air). The product was extracted with EtOAc, dried with $MgSO_4$ and concentrated in vacuo. The crude product was purified by flash column chromatography on silica gel (hexane/EtOAc 5:1) to afford the **C2** S−S dimer as yellow solid (35 mg, 70% yield). A minor amount of the **C2** C−C dimer was also isolated as a yellow solid (9 mg, 18% yield).

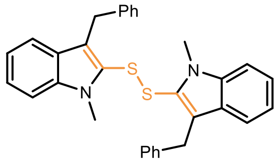

C2 S–S Dimer

1,2-Bis(3-benzyl-1-methyl-1H-indol-2-yl)disulfane (C2 S−S Dimer)

^{1}H NMR (500 MHz, CDCl$_3$) δ 7.42 (d, *J* = 8.0 Hz, 2H), 7.33 – 7.28 (m, 4H), 7.13 – 7.00 (m, 12H), 3.69 (br s, 4H), 3.63 (s, 6H).

^{13}C NMR (126 MHz, CDCl$_3$) δ 141.0, 138.8, 128.5, 128.3, 127.6, 126.7, 125.8, 124.4, 124.3, 120.5, 110.0, 31.1, 30.1.

HRMS (ESI$^+$) $C_{32}H_{29}N_2S_2$ [M+H]$^+$: found 505.1763, required 505.1767.

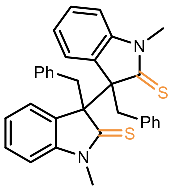

C2 C–C Dimer

3,3'-dibenzyl-1,1'-dimethyl-[3,3'-biindoline]-2,2'-dithione (C2 C−C Dimer)

^{1}H NMR (500 MHz, $CDCl_3$) δ 7.30 (td, *J* = 7.7, 1.3 Hz, 2H), 7.28 – 7.26 (m, 2H), 7.15 (td, *J* = 7.5, 0.9 Hz, 2H), 7.12 – 7.03 (m, 6H), 6.88 – 6.84 (m, 4H), 6.79 (d, *J* = 7.9 Hz, 2H), 3.42 – 3.36 (m, 8H), 3.07 (d, *J* = 12.7 Hz, 2H).

^{13}C NMR (126 MHz, $CDCl_3$) δ 208.29, 144.55, 134.23, 133.43, 130.23, 129.68, 126.92, 125.23, 124.42, 109.47, 84.92, 49.02, 31.27.

Synthesis of an authentic sample of the **C3 S-S Dimer:**

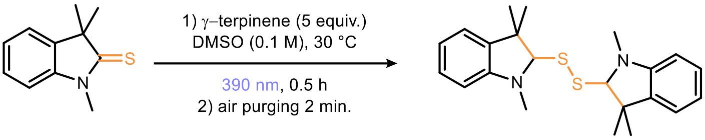

C3 S–S Dimer

Catalyst **C3** (20 mg, 0.1 mmol, 1 equiv.) was added to a 7 mL glass vial containing a stirring bar. The vial was sealed with a screw-top cap with septum and then evacuated three times for 2 minutes and backfilled with argon. Oxygen-free DMSO (1 mL, 0.1 M) was added, and the solution was stirred until the catalyst was completely dissolved. Then, γ-terpinene (84 µL, 0.5 mmol, 5 equiv.) was added via syringe. The vial was placed in the photoreactor (Figure S5) and stirred (600 rpm) at 30 °C (reactor temperature) under 390 nm irradiation (2 × PR160L-390 nm, 100% light intensity) for 0.5 hours. Then the reaction mixture was then purged with air for 2 minutes. The product was extracted with diethyl ether and water, dried over $MgSO_4$, and concentrated in vacuo, providing the **C3** S-S dimer as a colourless liquid (18 mg, 94%).

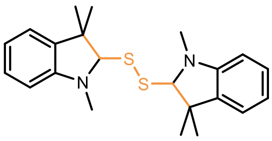

C3 S–S Dimer

1,2-bis(1,3,3-trimethylindolin-2-yl)disulfane (C3 S−S Dimer)

^{1}H NMR (500 MHz, $CDCl_3$) δ 7.14 (tt, J = 7.6, 1.1 Hz, 2H), 7.06 (dd, J = 7.2, 1.5 Hz, 2H), 6.79 (td, J = 7.4, 1.0 Hz, 2H), 6.54 (dd, J = 7.8, 5.1 Hz, 2H), 4.28 (s, 1H), 4.25 (s, 1H), 3.02 (s, 3H), 2.92 (s, 3H), 1.52 (s, 3H), 1.44 (s, 3H), 1.40 (s, 3H), 1.37 (s, 3H).

^{13}C NMR (126 MHz, $CDCl_3$) δ 149.3, 127.8, 121.6, 121.4, 118.9, 118.9, 107.5, 87.7, 86.7, 45.6, 45.5, 33.6, 33.4, 28.2, 26.7, 26.6, 25.0, 24.7.

HRMS (ESI^{+}) $C_{22}H_{28}N_2S_2$ $[M+H]^{+}$: found 385.1699, required 385.1697.

D. Optimization Studies

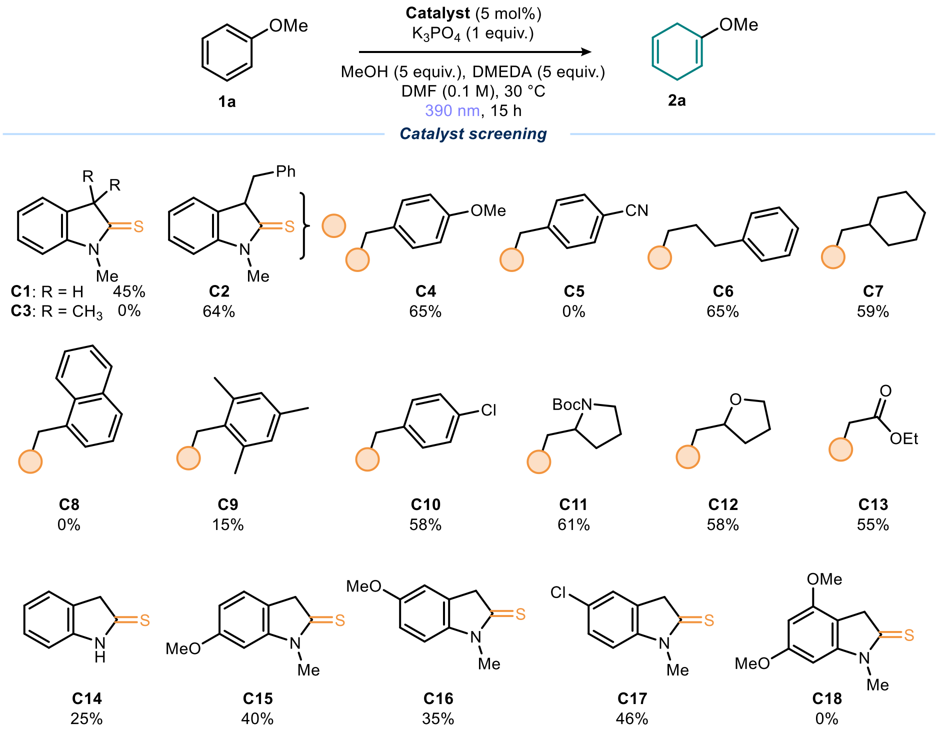

Figure S2. Evaluating the reactivity of different catalyst structures.

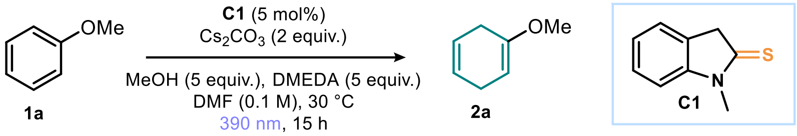

Reaction optimization

entry	deviation	yield [%] **2a**	entry	deviation	yield [%] **2a**
1	none	45	6	2.5 mol% **C1**	35
2	^{i}PrOH	44	7	10 mol% **C1**	45
3	^{t}BuOH	30	8	10 equiv. MeOH	31
4	HFIP	0	9	10 equiv. DMEDA	44
5	Ethylene glycol	39	10	+ 100 μL H_2O	0

Figure S3. Initial reaction optimization using **C1** as catalyst.

a)

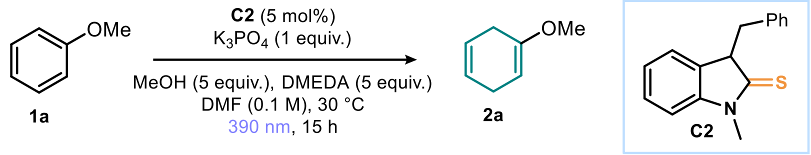

base screening

entry	deviation	yield [%] **2a**	entry	deviation	yield [%] **2a**
1	none	64	7	K_2HPO_4	0
2	Cs_2CO_3	57	8	TMEDA	21
3	KOMe	66	9	EDA	29
4	KOH	62	10	piperazine	33
5	K_2CO_3	<5	11	DABCO	26
6	NaOMe	0	12	DIPEA	24

b)

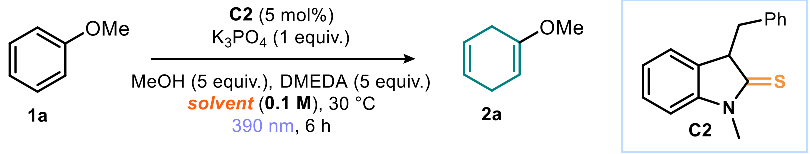

entry	solvent	yield [%] **2a**	entry	solvent	yield [%] **2a**
1	DMF	31	7	THF	0
2	DMF (0.05 M)	23	8	1,4-Dioxane	0
3	DMF (0.2 M)	traces	9	MeOH	0
4	NMP	14	10	MTBE	0
5	DMSO	0	11	Hexane	0
6	ACN	0	12	EtOAc	0

Figure S4. Further optimization of reaction conditions using catalyst **C2**: **a**) screening of various bases and amines; b) evaluation of different solvents and reaction concentrations. TMEDA = tetramethylethylenediamine; EDA = ethylenediamine.

E. Experimental Procedures

E.1. General Procedure for the Birch Reductions of Benzene Derivatives

General procedure **B**:

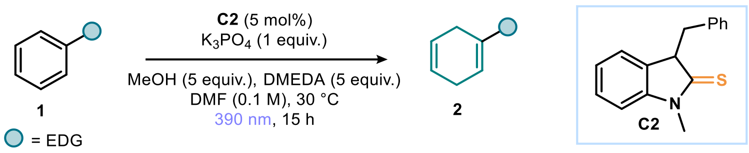

Catalyst **C2** (2.6 mg, 0.01 mmol, 0.05 equiv.), the arene substrate **1** (*if solid*, 0.2 mmol, 1 equiv.) and K_3PO_4 as powder (42 mg, 0.2 mmol, 1 equiv.) were added sequentially to a 7 mL glass vial containing a stirring bar. The vial was sealed with a screw-top cap with septum and then three times vacuumed for 2 minutes and backfilled with argon. Oxygen-free DMF (2 mL, 0.1 M) was added and the solution was stirred until the catalyst was completely dissolved. Then, MeOH (41 µL, 1.0 mmol, 5 equiv.), the arene (*if liquid*, 0.2 mmol, 1 equiv.), and DMEDA (104 µL, 1.0 mmol, 5 equiv.) were added via syringe. The vial was placed in the photoreactor (Figure S5) and stirred (600 rpm) at 30 ºC (reactor temperature) under 390 nm irradiation (2x PR160L-390 nm, 100% light intensity) for 15 hours.

After the reaction was completed, the mixture was filtered and then the solvent was removed under reduced pressure (40 °C, < 15 mbar). The crude residue was purified by column chromatography to afford the corresponding product **2** with the reported yields.

For NMR analyses of products **2a-2d** and **2g**: mesitylene or CH_2Br_2 was added as the internal standard (0.3 mmol, 1 equiv.) to the crude reaction mixture, followed by the addition of H_2O. The solution was extracted twice with 0.7 mL of $CDCl_3$, dried with $MgSO_4$ and then analysed *via* ^{1}H NMR.

Set-up for small scale reactions

The reactions were performed under illumination by two *Kessil* lamps (PR160L-390 nm, 100% intensity) using a 3D-printed, temperature controlled photoreactor reported in the literature.[3] It was equipped with vial holders accommodating either 4 or 6 positions, allowing parallel experiments. The reactor temperature was regulated using two thermoelectric coolers (Peltier elements) integrated into the reactor walls. The heat produced by the thermoelectric coolers was dissipated through an external water cycle that was not actively cooled. (Figure S5).

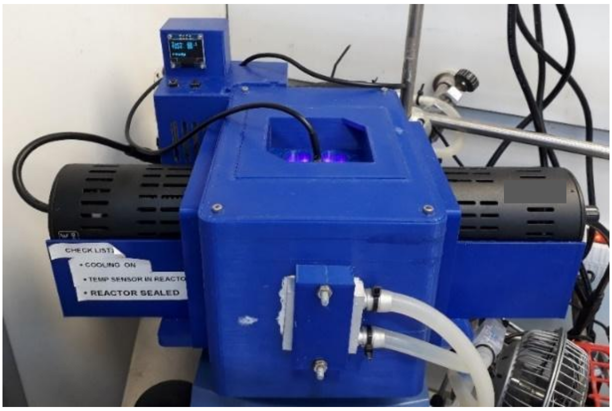

Figure S5. Reaction set-up for small-scale reactions using a 3D-printed, temperature controlled photoreactor,[3] illuminated at 390 nm with two Kessil PR160L lamps.

Troubleshooting and Practical Notes:

- Best results were obtained using powdered K_3PO_4
- Pre-stirring of the catalyst (1 minute, to ensure deprotonation) is suggested to avoid reproducibility issues.
- Substrates bearing *amine groups*: when used as HCl salts, reactivity is suppressed; the corresponding free amines, however, undergo effective reduction.
- The reaction is not moisture-sensitive but it is oxygen-sensitive. Normal Schlenk technique is sufficient to remove oxygen. Oxygen free DMF was used from a septum-sealed bottle but the other reagents were used without degassing (for the small-scale reactions).
- Products **2** & **4** are stable for short handling under air but undergo slow oxidation back to the arene; long-term storage requires an inert atmosphere.
- For selected compounds (as noted in specific cases below), additional purification was performed by column chromatography on 2 w/w $AgNO_3$-impregnated silica to separate product from residual starting material and obtain clean NMR spectra.[4] To prepare the impregnated silica, $AgNO_3$ was dissolved in MeCN, silica was added to the solution, and the solvent was removed under reduced pressure. The resulting material was used directly for chromatography. $AgNO_3$-impregnated silica had to be stored under exclusion of light, and staining solutions were required when using TLC plates.

Characterization of Products

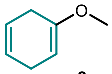
2a

1-Methoxycyclohexa-1,4-diene (2a)

Synthesized according to the general procedure **B** using anisole (33 µL, 0.3 mmol). After completion of the reaction, mesitylene was added as the internal standard (41 µL, 0.3 mmol, 1 equiv.) to the reaction mixture. The yield of **2a** (64% yield) was determined via ^{1}H NMR analysis of the crude mixture.

Characterization data matching reported literature data.[5]

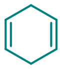
2b

Cyclohexa-1,4-diene (2b)

Synthesized according to the general procedure **B** using benzene (27 µL, 0.3 mmol). After completion of the reaction, mesitylene was added as the internal standard (41 µL, 0.3 mmol, 1 equiv.) to the reaction mixture. The yield of **2b** (53% yield) was determined via ^{1}H NMR analysis of the crude mixture.

Characterization data matching reported literature data.[6]

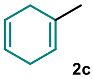
2c

1-Methylcyclohexa-1,4-diene (2c)

Synthesized according to the general procedure **B** using toluene (32 µL, 0.3 mmol). After completion of the reaction, mesitylene was added as the internal standard (41 µL, 0.3 mmol, 1 equiv.) to the reaction mixture. The yield of **2c** (52% yield) was determined via ^{1}H NMR analysis of the crude mixture.

Characterization data matching reported literature data.[7]

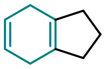
2d

2,3,4,7-Tetrahydro-1*H*-ndene (2d)

Synthesized according to the general procedure **B** using 2,3-dihydro-1H-indene (37 µL, 0.3 mmol). After completion of the reaction, mesitylene was added as the internal standard (41 µL, 0.3 mmol, 1 equiv.) to the reaction mixture. The yield of **2d** (58% yield) was determined via ^{1}H NMR analysis of the crude mixture.

Characterization data matching reported literature data.[8]

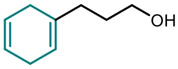

2e

3-(cyclohexa-1,4-dien-1-yl)-Propan-1-ol (2e)

Synthesized according to the general procedure **B** using 3-phenylpropan-1-ol (27 mg, 0.2 mmol). The crude mixture was purified by flash column chromatography using silica gel impregnated with 2% w/w $AgNO_3$ (hexane/AcOEt, 2:1) to afford **2e** as a yellow liquid (14.4 mg, 52% yield).

^{1}H NMR (600 MHz, CDCl$_3$) δ 5.73 – 5.65 (m, 2H), 5.46 – 5.42 (m, 1H), 3.63 (t, *J* = 6.5 Hz, 2H), 2.71 – 2.64 (m, 2H), 2.61 – 2.56 (m, 2H), 2.05 – 2.00 (m, 3H), 1.71 – 1.65 (m, 2H).

^{13}C NMR (151 MHz, CDCl$_3$) δ 134.6, 124.4, 124.3, 118.8, 62.8, 33.8, 30.3, 29.0, 26.7.

HRMS (ESI$^+$) $C_9H_{14}O$ [M]$^+$: found 138.1045, required 138.1039.

Characterization data matching reported literature data.[8]

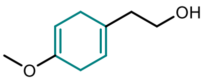

2f

2-(4-methoxycyclohexa-1,4-dien-1-yl)-Ethan-1-ol (2f)

Synthesized according to the general procedure **B** using 2-(4-methoxyphenyl)ethan-1-ol (30.4 mg, 0.2 mmol). The crude mixture was purified by flash column chromatography on silica gel (hexane/EtOAc 3:1 to 3:2) to afford **2f** as a colourless liquid (18.5 mg, 60% yield).

Note: Aqueous work up results in the formation of the correspond ketone (instead of the methoxy group).

^{1}H NMR (600 MHz, CDCl$_3$) δ 5.53 – 5.48 (m, 1H), 4.65 – 4.60 (m, 1H), 3.70 (t, *J* = 6.2 Hz, 2H), 3.55 (s, 3H), 2.80 – 2.72 (m, 4H), 2.28 (t, *J* = 6.3 Hz, 2H).

^{13}C NMR (151 MHz, CDCl$_3$) δ 153.0, 132.0, 120.6, 90.4, 60.4, 54.1, 39.9, 29.3, 29.3.

Characterization data matching reported literature data.[8]

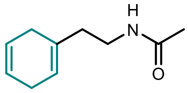

2g

***N*-(2-(cyclohexa-1,4-dien-1-yl)ethyl)-Acetamide (2g)**

Synthesized according to the general procedure **B** using *N*-(2-phenylethyl)acetamide (49.0 mg, 0.3 mmol, 1 equiv.). The crude mixture was purified by flash column chromatography on silica gel loaded with 2% $AgNO_3$ (EtOAc/DCM 2:1) to afford **2g** as a white solid (29.4 mg, 57% yield).

^{1}H NMR (500 MHz, CDCl$_3$) δ 5.75 – 5.67 (m, 2H), 5.52 – 5.47 (m, 1H), 5.44 (br s, 1H), 3.35 (td, *J* = 6.8, 5.5 Hz, 2H), 2.75 – 2.67 (m, 2H), 2.63 – 2.56 (m, 2H), 2.20 – 2.14 (m, 2H), 1.96 (s, 3H).

^{13}C NMR (126 MHz, CDCl$_3$) δ 170.1, 132.1, 124.3, 124.1, 121.0, 37.1, 37.0, 28.7, 26.9, 23.5.

Characterization data matching reported literature data.[9]

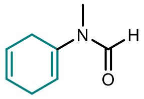

2h

***N*-(cyclohexa-1,4-dien-1-yl)-*N*-Methylformamide (2h)**

Synthesized according to the general procedure **B** using *N*-methyl-*N*-phenylformamide (27 mg, 0.2 mmol). The crude mixture was purified by flash column chromatography using silica gel impregnated with 2% w/w $AgNO_3$ (hexane/EtOAc, 3:1) to afford **2h** as a colourless liquid (12 mg, 44% yield).

^{1}**H NMR (600 MHz, CDCl$_3$)** δ 8.30 (s, 1H), 5.77 – 5.69 (m, 2H), 5.46 – 5.42 (m, 1H), 3.01 (d, *J* = 0.6 Hz, 3H), 2.91 – 2.80 (m, 4H).

^{13}**C NMR (151 MHz, CDCl$_3$)** δ 161.2, 135.3, 124.0, 122. 7, 114.6, 29.2, 27.5, 26.7.

HRMS (ESI$^+$) $C_8H_{12}NO$ [M+H]$^+$: found 138.0919, required 138.0913.

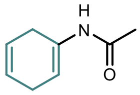

2i

***N*-(cyclohexa-1,4-dien-1-yl)acetamide (2i)**

Synthesized according to the general procedure **B** using N-phenylacetamide (27.0 mg, 0.2 mmol). The crude mixture was purified by flash column chromatography on silica gel (DCM/MeOH 98:2) to afford **2i** as a 1:1 mixture with respect to the starting material (22.0 mg, 50% purity, 40 % yield). The yield of the isolated product is in agreement to the yield determined via ^{1}H NMR analysis of the crude mixture using an internal standard (37% yield).

^{1}**H NMR (600 MHz, CDCl$_3$)** δ 6.63 (bs, 1H), 6.07 – 6.02 (m, 1H), 5.72 – 5.61 (m, 2H), 2.86 – 2.74 (m, 4H), 2.03 (s, 3H).

^{13}**C NMR (151 MHz, CDCl$_3$)** δ 169.0, 130.5, 124.6, 122.9, 110.7, 28.8, 26.3, 24.8.

HRMS (ESI$^+$) $C_8H_{11}NONa$ [M+Na]$^+$: found 160.0735, required 160.0733.

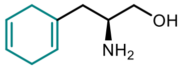

2j

(*S*)-2-Amino-3-(cyclohex-1-en-1-yl)propan-1-ol (2j)

Synthesized according to the general procedure **B** using (S)-2-amino-3-phenylpropan-1-ol (30.2 mg, 0.2 mmol). The crude mixture was purified by flash column chromatography on silica gel (DCM/MeOH 97:3) to afford **2j** as a 2:1 mixture with respect to the starting material (26.3 mg, 66% purity, 57 % yield). The yield of the isolated product is in agreement to the yield determined via ^{1}H NMR analysis of the crude mixture using an internal standard (55% yield).

^{1}**H NMR (600 MHz, CDCl$_3$)** δ 5.71 – 5.66 (m, 2H), 5.52 – 5.46 (m, 1H), 3.57 (dd, *J* = 10.7, 3.8 Hz, 1H), 3.34 – 3.29 (m, 1H), 3.04 – 2.97 (m, 1H), 2.72 – 2.51 (m, 4H), 2.09 – 2.04 (m, 1H), 1.91 (dd, *J* = 13.5, 9.1 Hz, 1H).

^{13}**C NMR (151 MHz, CDCl$_3$)** δ 131.7, 124.3, 124.1, 121.8, 66.8, 49.9, 42.6, 29.0, 26.9.

HRMS (ESI$^+$) $C_9H_{16}NO$ [M+H]$^+$: found 154.1198, required 154.1126.

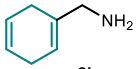

2k

Cyclohexa-1,4-dien-1-ylmethanamine (2k)

Synthesized according to the general procedure **B** using phenylmethanamine (21.4 mg, 0.2 mmol). After completion of the reaction, dibromomethane was added as the internal standard (13.9 µL, 0.2 mmol, 1 equiv.) to the reaction mixture. The yield of **2k** (52% yield) was determined via ^{1}H NMR analysis of the crude mixture.

Characterization data matching reported literature data.[10]

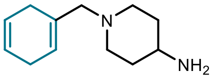

2l

1-(Cyclohexa-1,4-dien-1-ylmethyl)-Piperidin-4-amine (2l)

Synthesized according to the general procedure **B** using 1-benzylpiperidin-4-amine (38.1 mg, 0.2 mmol). The crude mixture was purified by flash column chromatography on silica gel (DCM:MeOH:NH_4OH 85:14:1) to afford **2l** as a 1:1.5 mixture with respect to the starting material (38 mg, 40% purity, 39% yield). The Yield of the isolated product is in agreement to the yield determined via ^{1}H NMR analysis of the crude mixture using an internal standard (42 % yield).

^{1}H NMR (600 MHz, $CDCl_3$) δ 5.77 – 5.60 (m, 2H), 5.59 – 5.54 (m, 1H), 2.81 (s, 2H), 2.80 – 2.75 (m, 2H), 2.73 – 2.57 (m, 5+3H), 1.87 (t, *J* = 11.6 Hz, 2H), 1.78 (d, *J* = 12.3 Hz, 2+2H), 1.42 – 1.33 (m, 2+2H).

^{13}C NMR (151 MHz, $CDCl_3$) δ 138.8, 133.1, 129.5, 128.5, 128.4, 127.3, 125.0, 124.7, 124.2, 122.0, 65.9, 63.4, 52.9, 52.8, 49.3, 49.1, 36.3, 36.3, 28.4, 27.0.

HRMS (ESI$^+$) $C_{12}H_{21}N_2$ [M+H]$^+$: found 193.1704, required 193.1699.

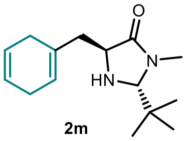

2m

(*2R,5S*)-2-(*tert*-Butyl)-5-(cyclohexa-1,4-dien-1-ylmethyl)-3-methylimidazolidin-4-one (2m)

Synthesized according to the general procedure **B** using (*2R,5S*)-5-benzyl-2-(*tert*-butyl)-3-methylimidazolidin-4-one (45.4 µL, 49.3 mg, 0.3 mmol, 1 equiv.). The crude mixture was purified by flash column chromatography on silica gel (hexane/EtOAc 2:1) to afford **2m** as a white/yellow solid (24 mg, 49% yield). The diastereomeric ratio (> 20:1) was determined by ^{1}H NMR analysis of the crude mixture and the purified product.

^{1}H NMR (500 MHz, $CDCl_3$) δ 5.94 (s, 1H), 5.89 – 5.80 (m, 2H), 4.25 (d, *J* = 1.5 Hz, 1H), 3.89 (dd, *J* = 10.1, 3.8 Hz, 1H), 3.32 (s, 1H), 2.96 (s, 3H), 2.90 – 2.72 (m, 3H), 2.61 (d, *J* = 14.5 Hz, 1H), 2.58 – 2.45 (m, 1H), 2.15 (dd, *J* = 14.6, 10.0 Hz, 1H), 1.04 (s, 9H).

^{13}C NMR (126 MHz, $CDCl_3$) δ 174.8, 133.2, 123.2, 122.9, 121.1, 83.9, 56.6, 41.3, 37.0, 31.5, 28.5, 27.5, 25.6.

HRMS (ESI$^+$) $C_{15}H_{25}N_2O$ [M+H]$^+$: found 249.1958, required 249.1961.

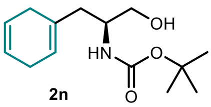

2n

***tert*-Butyl-(*S*)-(1-(cyclohexa-1,4-dien-1-yl)-3-hydroxypropan-2-yl)-carbamate (2n)**

Synthesized according to the general procedure **B** using *tert*-butyl (*S*)-(1-hydroxy-3-phenylpropan-2-yl)carbamate (50.3 mg, 0.2 mmol). The crude mixture was purified by flash column chromatography on silica gel (hexane/EtOAc 9:1 to 8:2) to afford

2n as a white solid (27.9 mg, 55% yield). The enantiomeric excess (> 99%) was determined by HPLC analysis on a chiral Daicel Chiralcel OJ-H column (eluent: n-hexane/i-PrOH 98:2; flow rate1 mL/min, λ = 210 nm, Scheme S1).

^{1}H NMR (600 MHz, CDCl$_3$) δ 5.73 – 5.66 (m, 2H), 5.53 – 5.48 (m, 1H), 4.62 (br s, 1H), 3.77 (br s, 1H), 3.70 – 3.65 (m, 1H), 3.60 – 3.54 (m, 1H), 2.85 – 2.45 (m, 5H), 2.19 (dd, *J* = 14.1, 6.4 Hz, 1H), 2.10 (dd, *J* = 14.0, 8.6 Hz, 1H), 1.44 (s, 9H).

^{13}C NMR (151 MHz, CDCl$_3$) δ 156.8, 131.4, 124.2, 124.1, 122.3, 80.0, 66.4, 50.7, 39.8, 28.8, 28.5, 26.9.

HRMS (MALDI-TOF$^+$) $C_{14}H_{23}NO_3Na$ [M+Na]$^+$: found 276.1576, required 276.1570.

a) HPLC trace of the racemic mixture of **1n** and **2n**

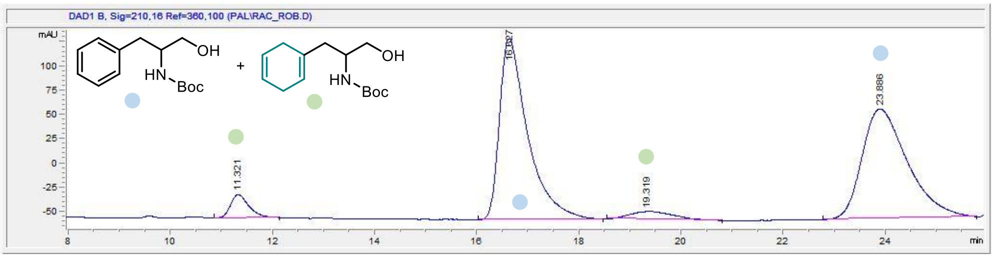

#	Time	Area	Height	Width	Area%	Symmetry
1	11.321	603.5	24.1	0.3702	3.955	0.68
2	16.627	7171.2	186.6	0.5624	46.992	0.478
3	19.319	493.1	8.1	0.7199	3.231	0.604
4	23.886	6992.6	113	0.8377	45.822	0.608

b) HPLC trace of the isolated product using enantiopure starting material **1n**

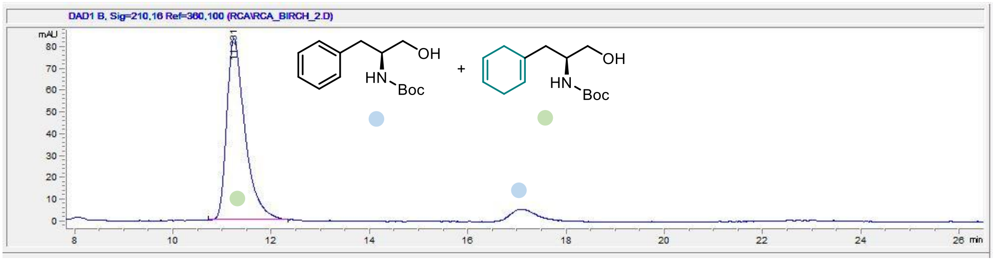

#	Time	Area	Height	Width	Area%	Symmetry
1	11.231	2156.6	83.3	0.39	100.000	0.591

Scheme S1. HPLC traces of a) the racemic mixture of **1n** and **2n** and b) the isolated product **2n**, containing traces of remaining starting material **1n**.

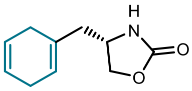

2o

(*S*)-4-(Cyclohexa-1,4-dien-1-ylmethyl)-oxazolidin-2-one (2o)

Synthesized according to the general procedure **B** using (*S*)-4-benzyl-2-oxazolidinone (53.2 mg, 0.3 mmol) The crude mixture was purified by flash column chromatography on silica gel (hexanes/EtOAc 2:1) to afford **2o** as a white solid (29 mg, 54%yield).

^{1}H NMR (500 MHz, CDCl$_3$) δ 5.75 – 5.65 (m, 2H), 5.56 – 5.50 (m, 1H), 5.34 (br s, 1H), 4.49 (t, J = 8.2 Hz, 1H), 4.05 (dd, J = 8.4, 5.6 Hz, 1H), 4.00 (tt, J = 8.1, 5.9 Hz, 1H), 2.75 – 2.66 (m, 2H), 2.67 – 2.49 (m, 2H), 2.23 (qd, J = 14.0, 6.9 Hz, 2H).

^{13}C NMR (126 MHz, CDCl$_3$) δ 159.3, 130.0, 124.4, 123.6, 122.9, 70.1, 50.3, 43.5, 29.2, 26.8.

HRMS (ESI$^+$) $C_{10}H_{13}NO_2Na$ [M+Na]$^+$: found 202.0844, required 202.0838.

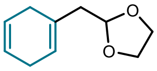

2p

2-(Cyclohexa-1,4-dien-1-ylmethyl)-1,3-dioxolane (2p)

Synthesized according to the general procedure **B** using 2-benzyl-1,3-dioxolane (32.8 mg, 0.2 mmol). The crude mixture was purified by flash column chromatography on silica gel (hexane/EtOAc 99:1 to 97:3) to afford **2p** as a yellowish oil (14.3 mg, 43% yield).

^{1}H NMR (600 MHz, CDCl$_3$) δ 5.73 – 5.65 (m, 2H), 5.59 – 5.55 (m, 1H), 4.95 (t, J = 5.1 Hz, 1H), 4.03 – 3.94 (m, 2H), 3.90 – 3.81 (m, 2H), 2.72 – 2.62 (m, 4H), 2.33 (d, J = 5.1 Hz, 2H).

^{13}C NMR (151 MHz, CDCl$_3$) δ 130.4, 124.3, 124.1, 122.0, 103.8, 65.0, 42.3, 29.8, 26.9.

HRMS (ESI$^+$) $C_{10}H_{14}O_2Na$ [M+Na]$^+$: found 189.0891, required 189.0886.

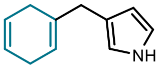

2q

3-(Cyclohexa-1,4-dien-1-ylmethyl)-1H-pyrrole (2q)

Synthesized according to the general procedure **B** using 3-benzyl-1H-pyrrole (31.5 mg, 0.2 mmol). The crude mixture was purified by flash column chromatography on silica gel (hexane/EtOAc 98:2) to afford **2q** as a colourless oil (6 mg, 19 % yield). The yield of **2q** determined via ^{1}H NMR analysis of the crude mixture was 22% using CH_2Br_2 as an internal standard.

^{1}H NMR (600 MHz, CDCl$_3$) δ 8.03 (s, 1H), 6.73 (q, J = 2.5 Hz, 1H), 6.59 (q, J = 2.3 Hz, 1H), 6.08 (q, J = 2.7 Hz, 1H), 5.73 – 5.66 (m, 2H), 5.50 – 5.47 (m, 1H), 3.15 (s, 2H), 2.73 – 2.65 (m, 2H), 2.65 – 2.54 (m, 2H).

^{13}C NMR (151 MHz, CDCl$_3$) δ 135.5, 124.7, 124.2, 121.7, 118.8, 117.8, 115.9, 109.4, 35.5, 29.1, 27.0.

HRMS (TOF MS LD+) $C_{11}H_{14}N$ [M+H]$^+$: found 160.1125, required 160.1121.

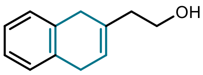

2r

2-(1,4-Dihydronaphthalen-2-yl)-ethan-1-ol (2r)

Synthesized according to the general procedure **B** using 2-(naphthalen-2-yl)ethan-1-ol (51.7 mg, 0.3 mmol). The crude mixture was purified by flash column chromatography on silica gel (hexane/EtOAc 95:5) to afford **2r** as a colourless liquid (28 mg, 54 % yield).

^{1}H NMR (600 MHz, CDCl$_3$) δ 7.19 – 7.10 (m, 4H), 5.79 – 5.70 (m, 1H), 3.85 – 3.68 (m, 3H), 3.50 – 3.39 (m, 2H), 3.36 (t, J = 1.4 Hz, 2H), 2.40 (t, J = 0.7 Hz, 2H).

^{13}C NMR (151 MHz, CDCl$_3$) δ 134.0, 133.9, 132.1, 128.2, 128.1, 125.9, 125.9, 121.7, 60.1, 40.2, 32.8, 30.4.

HRMS (TOF MS LD+) $C_{13}H_{17}O$ [M+H]$^+$: found 175.1123, required 175.1117.

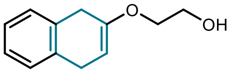

2s

2-((1,4-Dihydronaphthalen-2-yl)oxy)-ethan-1-ol (2s)

Synthesized according to the general procedure **B** using 2-(naphthalen-2-yloxy)ethanol (18.8 mg, 0.1 mmol). The crude mixture was purified by flash column chromatography on silica gel (hexane/EtOAc 95:5 to 85:15) to afford **2s** as a white solid (15.6 mg, 82% yield).

^{1}H NMR (600 MHz, CDCl$_3$) δ 7.19 – 7.11 (m, 4H), 4.86 (t, *J* = 3.6 Hz, 1H), 3.91 – 3.88 (m, 4H), 3.52 – 3.48 (m, 2H), 3.48 – 3.44 (m, 2H), 2.03 – 1.95 (m, 1H).

^{13}C NMR (151 MHz, CDCl$_3$) δ 152.3, 134.1, 133.5, 128.3, 128.0, 126.0, 125.9, 91.9, 67.8, 61.5, 32.2, 29.5.

HRMS (ESI$^+$) $C_{12}H_{15}O_2$ [M+H]$^+$: found 191.1028, required 191.1067.

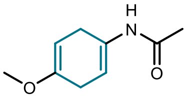

2t

***N*-(4-Methoxycyclohexa-1,4-dien-1-yl)-acetamide (2t)**

Synthesized according to the general procedure **B** using *N*-(4-methoxyphenyl)acetamide (33.0 mg, 0.2 mmol). The crude mixture was purified by flash column chromatography on silica gel (hexane/EtOAc 6:4 to 4:6) and then by reverse phase preparative HPLC on a Synergi 4u Hydro-RP 80A Prep Column (10 mm x 250 mm, 130 Å, 4 µm) (30% to 70% CH_3CN in H_2O) to afford **2t** as a white solid (6.3 mg, 19% yield). The yield of **2t** determined via ^{1}H NMR analysis of the crude mixture was 26% using CH_2Br_2 as an internal standard.

^{1}H NMR (600 MHz, CDCl$_3$) δ 6.33 (s, 1H), 6.10 – 6.05 (m, 1H), 4.56 (t, *J* = 3.6 Hz, 1H), 3.54 (s, 3H), 2.93 (td, *J* = 7.4, 3.1 Hz, 2H), 2.83 (td, *J* = 7.5, 3.7 Hz, 2H), 2.04 (s, 3H).

^{13}C NMR (151 MHz, CDCl$_3$) δ 168.5, 153.0, 130.5, 109.0, 89.1, 54.2, 29.0, 28.4, 24.7.

HRMS (ESI$^+$) $C_9H_{13}NO_2Na$ [M+Na]$^+$: found 190.0844, required 190.0838.

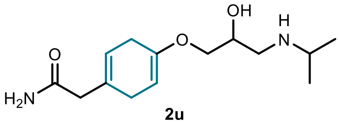

2u

2-(4-(2-Hydroxy-3-(isopropylamino)propoxy)cyclohexa-1,4-dien-1-yl)-acetamide (2u)

Synthesized according to the general procedure **B** using *Atenolol* (50.5 mg, 0.2 mmol). The crude mixture was subjected to removal of volatiles, and

the resulting residue was washed with DCM and hexane, to afford **2u** as pale-yellow solid (27.5 mg, 54% yield).

Note: Aqueous work up results in the formation of the correspond ketone (instead of the ether group).

^{1}H NMR (600 MHz, DMSO-*d*$_6$) δ 7.26 (s, 1H), 6.80 (s, 1H), 5.46 – 5.41 (m, 1H), 4.96 (br s, 1H), 4.61 (t, *J* = 2.9 Hz, 1H), 3.76 (m, 1H), 3.62 (dd, *J* = 9.9, 5.0 Hz, 1H), 3.56 (dd, *J* = 9.9, 6.0 Hz, 1H), 2.82 – 2.75 (m, 1H), 2.74 (s, 2H), 2.66 (m, 5H), 2.55 – 2.51 (m, 2H), 0.99 (dd, *J* = 6.4, 1.6 Hz, 6H).

^{13}C NMR (151 MHz, DMSO-*d*$_6$) δ 172.0, 151.2, 130.6, 120.2, 91.0, 69.2, 67.7, 49.7, 48.5, 43.4, 29.1, 28.9, 22.3.

HRMS (ESI$^+$) $C_{14}H_{24}N_2O_3Na$ [M+Na]$^+$: found 291.1685, required 291.1679.

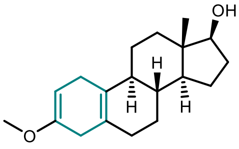

2v

(*8R,9S,13S,14S*)-3-Methoxy-13-methyl-4,6,7,8,9,11,12,13,14,15,16,17-dodecahydro-1*H*-cyclopenta[a]phenanthren-17-ol (2v)

Synthesized according to the general procedure **B** using (*8R,9S,13S,14S*)-3-methoxy-13-methyl-7,8,9,11,12,13,14,15,16,17-decahydro-6*H*-cyclopenta[a]phenanthren-17-ol (28.6 mg, 0.1 mmol). The crude mixture was purified by flash column chromatography on silica gel (hexane/EtOAc 8:2 to 6:1) to afford **2v** as a white solid (4.0 mg, 14% yield).

^{1}H NMR (600 MHz, $CDCl_3$) δ 4.67 – 4.62 (m, 1H), 3.72 – 3.64 (m, 1H), 3.55 (s, 3H), 2.91 – 2.84 (m, 1H), 2.71 – 2.57 (m, 2H), 2.57 – 2.49 (m, 1H), 2.13 – 2.03 (m, 2H), 1.91 – 1.81 (m, 3H), 1.73 – 1.68 (m, 1H), 1.66 – 1.59 (m, 2H), 1.49 – 1.08 (m, 8H), 0.76 (s, 3H).

^{13}C NMR (151 MHz, $CDCl_3$) δ 152.8, 128.0, 125.1, 90.8, 82.1, 54.0, 50.1, 45.6, 43.5, 39.1, 37.1, 34.3, 30.8, 30.6, 28.5, 26.9, 25.5, 23.2, 11.4.

Characterization data matching reported literature data.[11]

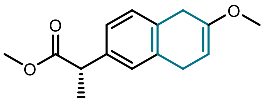

2w

Methyl (*S*)-2-(6-methoxy-5,8-dihydronaphthalen-2-yl)-propanoate (2w)

Synthesized according to the general procedure **B** using methyl (*S*)-2-(6-methoxynaphthalen-2-yl)propanoate (48.9 mg, 0.2 mmol). The crude mixture was purified by flash column chromatography on silica gel (hexane/EtOAc 20:1) to afford **2w** as a white solid (30 mg, 60% yield).

^{1}H NMR (400 MHz, $CDCl_3$) δ 7.09 (dd, *J* = 3.4, 1.7 Hz, 3H), 4.81 (tt, *J* = 3.8, 1.2 Hz, 1H), 3.71 – 3.67 (m, 1H), 3.66 (s, 3H), 3.61 (s, 3H), 3.49 (q, *J* = 4.4 Hz, 2H), 3.40 (t, *J* = 4.8 Hz, 2H), 1.49 (d, *J* = 7.2 Hz, 3H).

^{13}C NMR (101 MHz, $CDCl_3$) δ 175.3, 153.4, 138.4, 134.7, 132.8, 128.8, 127.1, 125.1, 90.7, 54.3, 52.2, 45.2, 32.0, 29.6, 18.8.

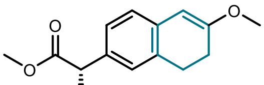

2w'

Methyl (*S*)-2-(6-methoxy-7,8-dihydronaphthalen-2-yl)propanoate (2w')

Synthesized according to general procedure **B** using methyl (S)-2-(6-methoxynaphthalen-2-yl)propanoate (48.9 mg, 0.2 mmol). The yield of **2w'** (22%) was determined by ^{1}H NMR analysis of the crude mixture. Pure **2w'** was obtained after isomerization of **2w** under air, followed by purification via flash column chromatography on silica gel (hexane/EtOAc 20:1), affording **2w**' as a white solid.

^{1}H NMR (400 MHz, $CDCl_3$) δ 7.05 – 6.98 (m, 2H), 6.90 (d, J = 7.7 Hz, 1H), 5.52 (s, 1H), 3.69 (s, 3H), 3.68 – 3.62 (m, 4H), 2.87 (t, J = 8.2 Hz, 2H), 2.39 (t, J = 8.1 Hz, 2H), 1.47 (d, J = 7.1 Hz, 3H).

^{13}C NMR (151 MHz, $CDCl_3$) δ 175.7, 160.7, 136.9, 134.9, 132.7, 126.5, 125.9, 125.2, 96.2, 55.2, 52.4, 45.4, 29.0, 27.6, 19.0.

HRMS (MALDI-TOF^{+}) $C_{15}H_{18}O_3Na$ [M+H]$^{+}$: found 269.1154, required 269.1148.

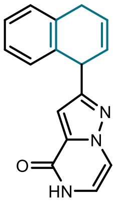

2x

2-(1,4-dihydronaphthalen-1-yl)pyrazolo[1,5-a]pyrazin-4(5H)-one (2x)

Synthesized according to the general procedure **B** using 2-(naphthalen-1-yl)pyrazolo[1,5-*a*]pyrazin-4(5*H*)-one (52.3 mg, 0.2 mmol). The crude mixture was purified by flash column chromatography on silica gel (DCM/MeOH 95:5) to afford **2x** as a mixture with the starting material **1x** and traces of the over-reduced product in a 1:2.2:0.2 ration (25.0 mg, 30% purity, 14% yield). The yield of the isolated product is in agreement to the yield determined via ^{1}H NMR analysis of the crude mixture using an internal standard (20% yield).

^{1}H NMR (600 MHz, $CDCl_3$) δ 10.60 – 10.42 (m, 1H), 7.48 – 7.46 (m, 1H), 7.22 – 7.12 (m, 4H), 6.74 (s, 1H), 6.61 (m, 1H), 6.15 – 6.09 (m, 1H), 6.09 – 6.04 (m, 1H), 4.96 (q, J = 4.1 Hz, 1H), 3.60 – 3.53 (m, 1H), 3.50 – 3.43 (m, 1H).

^{13}C NMR (151 MHz, $CDCl_3$) δ 158.7, 157.5, 135.7, 134.2, 133.7, 129.2, 128.6, 127.3, 126.7, 126.5, 125.6, 114.0, 111.7, 103.9, 39.4, 29.8.

HRMS (ESI^{+}) $C_{16}H_{14}N_3O$ [M+H]$^{+}$: found 264.1135, required 264.1131.

E.2. General procedure for the Birch Reduction of Heteroarenes

General procedure **C**:

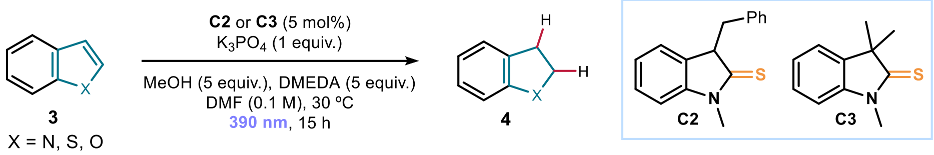

The catalyst **C2** (2.6 mg, 0.01 mmol, 0.05 equiv.) or **C3** (1.9 mg, 0.01 mmol, 0.05 equiv., for **4a** and **4b**), arenes (*if solid*, 0.2 mmol, 1 equiv.) and K_3PO_4 as powder (42 mg, 0.2 mmol, 1 equiv.) were added sequentially to a 7 mL glass vial containing a stirring bar. The vial was sealed with a screw-top cap with septum and then three times vacuumed for 2 minutes and backfilled with argon. Oxygen free DMF (2 mL, 0.1 M) was added and the solution was stirred until the catalyst was completely dissolved. Then MeOH (41 µL, 1.0 mmol, 5 equiv.), arenes (*if liquid*, 0.2 mmol, 1 equiv.) and DMEDA (104 µL, 1.0 mmol, 5 equiv.) were added via syringe. The vial was placed in the photoreactor and stirred (600 rpm) at 30 ºC (reactor temperature) under 390 nm irradiation (2x PR160L-390 nm, 100% light intensity) for 15 hours.

After the reaction was completed, the mixture was filtered and then the solvent was removed under reduced pressure (40 °C, < 15 mbar). The crude residue was purified by column chromatography to afford the corresponding product with the reported yields.

For NMR analyses of products **4a-4c**: Dibromomethane was added as the internal standard (0.3 mmol, 1 equiv.) to the crude reaction mixture, followed by the addition of H_2O. The solution was extracted twice with 0.7 mL of $CDCl_3$, dried with $MgSO_4$ and then analysed *via* ^{1}H NMR.

Characterization of Products

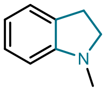

4a

1-Methylindoline (4a)

Synthesized according to the general procedure **C** using 1-methylindole (37 µL, 0.3 mmol). After completion of the reaction, dibromomethane was added as the internal standard (21 µL, 0.3 mmol, 1 equiv.) to the reaction mixture. The yield of **4a** (74% yield) was determined via ^{1}H NMR analysis of the crude mixture.

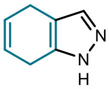

4b

4,7-Dihydro-1*H*-indazole (4b)

Synthesized according to the general procedure **C** using 1*H*-indazole (23.6 mg, 0.2 mmol). The crude mixture was purified by flash column chromatography on silica gel (hexane/EtOAc 98:2 to 7:3) to afford **4b** as a colourless liquid (8.4 mg, 35% yield).

The yield of the isolated product is in agreement to the yield determined via ^{1}H NMR analysis of the crude mixture using an internal standard (44% yield).

^{1}H NMR (500 MHz, $CDCl_3$) δ 7.38 (s, 1H), 5.97 – 5.80 (m, 2H), 3.38 – 3.32 (m, 2H), 3.28 – 3.22 (m, 2H).

^{13}C NMR (126 MHz, $CDCl_3$) δ 141.3, 131.4, 125.1, 123.1, 112.6, 23.2, 22.5.

HRMS (ESI$^+$) $C_7H_9N_2$ [M+H]$^+$: found 121.0766, required 121.0760.

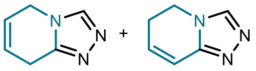

4c + 4c'

5,8-Dihydro-[1,2,4]triazolo[4,3-a]pyridine and 5,6-dihydro-[1,2,4]triazolo[4,3-a]pyridine (4c and 4c')

Synthesized according to the general procedure **C** using [1,2,4]triazolo [4,3-a]pyridine (23.8 mg, 0.2 mmol). The crude mixture was purified by flash column chromatography on silica gel (DCM/MeOH 98:2) to afford the mixture **4c/4c'** in a 1.2:1 ratio as a white solid (11 mg, 45% yield). The yield of the isolated product is in agreement to the yield determined via ^{1}H NMR analysis of the crude mixture using an internal standard (51% yield).

4c: ^{1}H NMR (600 MHz, $CDCl_3$) δ 8.17 (s, 1H), 6.07 (dtt, *J* = 10.3, 3.6, 2.3 Hz, 1H), 5.91 (dtt, *J* = 10.5, 3.0, 2.3 Hz, 1H), 4.66 (tdd, *J* = 5.0, 3.0, 2.3 Hz, 2H), 3.63 (tdd, *J* = 4.9, 3.6, 2.3 Hz, 2H).

4c': ^{1}H NMR (600 MHz, $CDCl_3$) δ 8.05 (s, 1H), 6.75 (dtd, *J* = 10.1, 2.0, 0.7 Hz, 1H), 6.27 (dt, *J* = 10.1, 4.4 Hz, 1H), 4.15 (t, *J* = 7.5 Hz, 2H), 2.64 (tdd, *J* = 7.5, 4.4, 2.0 Hz, 2H).

4c + 4c': ^{13}C NMR (151 MHz, $CDCl_3$) δ 149.7, 148.6, 141.8, 141.7, 130.8, 123.1, 119.4, 117.3, 43.5, 41.3, 23.9, 22.9.

HRMS (ESI$^+$) $C_6H_8N_3$ [M+H]$^+$: found 122.0718, required 122.0713.

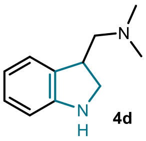

4d

1-(indolin-3-yl)-*N*,*N*-Dimethylmethanamine (4d)

Synthesized according to the general procedure **C** using 1-(1*H*-indol-3-yl)-*N*,*N*-dimethylmethanamine (52.3 mg, 0.3 mmol). The crude mixture was purified by flash column chromatography on silica gel (DCM/MeOH/Et_3N 98:1:1) to afford **4d** as a colourless liquid (11 mg, 20% yield). The yield of the isolated product is in agreement to the yield determined via ^{1}H NMR analysis of the crude mixture using an internal standard (26% yield).

^{1}H NMR (600 MHz, $CDCl_3$) δ 7.13 (d, *J* = 7.3 Hz, 1H), 7.03 (d, *J* = 7.6 Hz, 1H), 6.71 (td, *J* = 7.4, 1.0 Hz, 1H), 6.64 (d, *J* = 7.8 Hz, 1H), 3.69 (t, *J* = 8.7 Hz, 1H), 3.47 (d, *J* = 5.7 Hz, 1H), 3.39 (dd, *J* = 8.8, 8.7 Hz, 1H), 2.54 (d, *J* = 2.2 Hz, 1H), 2.53 (s, 1H), 2.30 (s, 6H).

^{13}C NMR (151 MHz, $CDCl_3$) δ 151.6, 131.4, 127.8, 124.2, 118.7, 109.7, 64.1, 52.4, 46.0, 40.5.

HRMS (ESI$^+$) $C_{11}H_{17}N_2$ [M+H]$^+$: found 177.1392, required 177.1386.

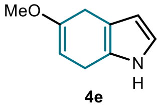

4e

4,5,6,7-Tetrahydro-1H-indole-5-carbonitrile (4e)

Synthesized according to the general procedure **C** using 5-methoxy-1H-indole (29.4 mg, 0.2 mmol). The crude mixture was purified by flash column chromatography on silica gel (hexane/EtOAc 98:2) to afford **4e** as a colourless liquid (4.5 mg, 15% yield). The yield of the isolated product is in agreement to the yield determined via ^{1}H NMR analysis of the crude mixture using an internal standard (22% yield).

^{1}H NMR (600 MHz, CDCl$_3$) δ 7.80 (s, 1H), 6.71 (t, J = 2.7 Hz, 1H), 6.03 (t, J = 2.6 Hz, 1H), 4.84 – 4.71 (m, 1H), 3.61 (s, 3H), 3.38 – 3.31 (m, 2H), 3.27 (t, J = 5.9 Hz, 2H).

^{13}C NMR (151 MHz, CDCl$_3$) δ 154.9, 124.1, 116.8, 113.7, 106.6, 89.8, 54.4, 27.3, 22.8.

HRMS (ESI$^+$) $C_9H_{12}NO$ [M+H]$^+$: found 150.0917, required 150.0913.

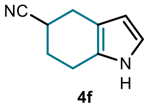

4f

4,7-Dihydro-3H-spiro[isobenzofuran-1,4'-piperidine] (4f)

Synthesized according to the general procedure **C** using 5-cyanoindole (28.4 mg, 0.2 mmol). The crude mixture was purified by flash column chromatography on silica gel (hexane/EtOAc 98:2) to afford **4f** as a colourless liquid (5.5 mg, 19% yield). The yield of the isolated product is in agreement to the yield determined via ^{1}H NMR analysis of the crude mixture using an internal standard (24% yield)

^{1}H NMR (600 MHz, CDCl$_3$) δ 7.82 (s, 1H), 6.66 (t, J = 2.7 Hz, 1H), 5.99 (t, J = 2.7 Hz, 1H), 3.00 – 2.90 (m, 2H), 2.89 – 2.73 (m, 2H), 2.73 – 2.64 (m, 1H), 2.26 – 2.14 (m, 1H), 2.14 – 2.06 (m, 1H).

^{13}C NMR (151 MHz, CDCl$_3$) δ 125.0, 122.6, 117.0, 113.4, 107.4, 27.0, 26.6, 26.1, 21.0.

HRMS (ESI$^+$) $C_9H_{11}N_2$ [M+H]$^+$: found 147.0921, required 147.0917.

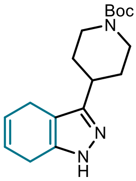

4g

tert-butyl 4-(4,7-dihydro-1H-indazol-3-yl)piperidine-1-carboxylate (4g)

Synthesized according to the general procedure **C** using tert-butyl 4-(1*H*-indazol-3-yl)piperidine-1-carboxylate (60.3 mg, 0.2 mmol). The crude mixture was purified by flash column chromatography on silica gel loaded with 2% $AgNO_3$ (DCM/MeOH 95:5) to afford **4g** as a dark solid (33.3 mg, 55% yield).

^{1}H NMR (600 MHz, CDCl$_3$) δ 5.91 – 5.83 (m, 2H), 4.19 (s, 2H), 3.32 – 3.27 (m, 2H), 3.21 – 3.17 (m, 2H), 2.85 – 2.66 (m, 3H), 1.90 – 1.81 (m, 2H), 1.74 – 1.65 (m, 2H), 1.46 (s, 9H).

^{13}C NMR (151 MHz, CDCl$_3$) δ 155.0, 124.8, 122.8, 112.5, 112.2, 109.4, 79.6, 44.6-43.8, 35.3, 30.9, 28.6, 23.2, 22.9.

HRMS (ESI$^+$) $C_{17}H_{25}N_3O_2Na$ [M+Na]$^+$: found 326.1842, required 326.1839.

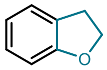

4h

2,3-Dihydrobenzofuran (4h)

Synthesized according to the general procedure **C** using 2,3-benzofuran (33 µL, 0.3 mmol). After completion of the reaction, dibromomethane was added as the internal standard (21 µL, 0.3 mmol, 1 equiv.) to the reaction mixture. The yield of **4h** (20% yield using **C2**, 40% yield using **C3**) was determined via ^{1}H NMR analysis of the crude mixture.

Characterization data matching reported literature data.**Error! Bookmark not defined.**

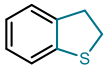

4i

2,3-Dihydrobenzo[b]thiophene (4i)

Synthesized according to the general procedure **C** using thianaphthene (40 mg, 0.3 mmol). After completion of the reaction, dibromomethane was added as the internal standard (21 µL, 0.3 mmol, 1 equiv.) to the reaction mixture. The yield of **4i** (50% yield using **C2**, 80% yield using **C3**) was determined via ^{1}H NMR analysis of the crude mixture.

Characterization data matching reported literature data.**Error! Bookmark not defined.**

E.3. Procedure for the telescoped Birch reduction/spirocyclization sequence

<u>General procedure **D**:</u>

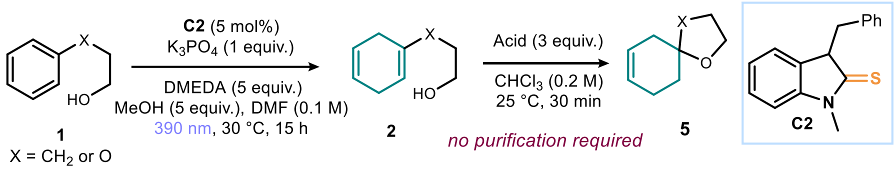

The arene reduction was performed according to general procedure **B**. After completion of the reaction, dibromomethane was added as the internal standard (1 equiv.) to the reaction mixture. The yield of **2** was determined via ^{1}H NMR analysis of the crude mixture. Afterwards the reaction mixture was filtered through a syringe filter (Acrodisc®) to remove solids and then the solvent and excess DMEDA were removed under reduced pressure (40 °C, < 15 mbar). In a 4 mL vial equipped with a magnetic stirring bar, the residue containing crude 1,4-dihydro product **2** was dissolved in $CHCl_3$ (under air). The vial was sealed with a screw-cap fitted with a septum, and the acid (3 equiv.) was added via syringe—trifluoroacetic acid (TFA) was used for acetal-derived products while triflic acid (TfOH) for cyclic ether-derived products. The reaction mixture was stirred at ambient temperature for 30 minutes.

After completion of the reaction, dibromomethane was added again as the internal standard (1 equiv.) to the reaction mixture. The yield of **5** was determined via ^{1}H NMR analysis of the

crude mixture based on the yield of 1,4-dihydro product **2**. In some cases, the crude residue was then purified by column chromatography to afford the corresponding product **5**.

<u>General procedure **E**:</u>

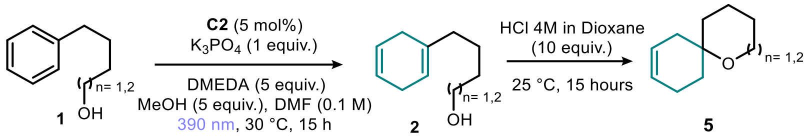

The arene reduction was performed according to general procedure **B**. After completion of the reaction, the crude mixture was purified by flash column chromatography on silica gel impregnated with 2% $AgNO_3$ (hexane/EtOAc 5:1) to afford **2.** The birch reduction product (0.05 mmol, 1 equiv.) was then transferred in a 4 mL vial equipped with a magnetic stirring bar. The vial was sealed with a screw-cap fitted with a septum, and the HCl 4M in dioxane (125 µL, 0.5 mmol, 10 equiv.) was added via syringe. The reaction mixture was stirred at ambient temperature for 15 hours.

After completion of the reaction, dibromomethane was added as the internal standard (1 equiv.) to the reaction mixture. The yield of **5** was determined via ^{1}H NMR analysis.

The reaction procedure had to be adapted due to the very high volatility of product **5ac** and **5ad**.

Characterization of Products

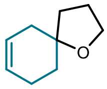

5e

1-Oxaspiro[4.5]dec-7-ene (5e)

Synthesized according to the general procedure **D** using 3-phenylpropan-1-ol (27 mg, 0.2 mmol) for the Birch reduction and TfOH (53 uL, 3 equiv.) in $CHCl_3$ (1 mL, 0.2 M) for the cyclization. The yields were determined by adding CH_2Br_2 (14.9 µL, 0.2 mmol, 1 equiv.) as internal standard to the reaction mixtures (49% for **2e**, 74% for **5e**, 36% over both steps).

Characterization data matching reported literature data.[12]

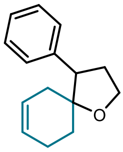

5z

3,4-Dihydro-1*H*-spiro[naphthalene-2,2'-[1,3]dioxolane] (5z)

Synthesized according to the general procedure **D** using 3,3-diphenyl-1-propanol (63.7 mg, 0.3 mmol) for the Birch reduction and TfOH (80 uL, 3 equiv.) in $CHCl_3$ (1.5 mL, 0.2 M) for the cyclization at 70 °C for 16 h. The yield of **2z** (38%) was determined by ^{1}H NMR analysis of the reaction crude after the Birch reduction. The crude mixture after the cyclization was purified by flash column

chromatography on silica gel (hexane) to afford **5z** in 43% yield, in a diastereomeric mixture (1:0.5) as a colourless oil (10.3 mg, 16% yield over both steps).

Diastereomeric mixture (1:0.5):

^{1}H NMR (600 MHz, CDCl$_3$) δ 7.33 – 7.27 (m, 2H d_1 + 1H d_2), 7.26 – 7.16 (m, 3H d_1 + 1.5H d_2), 5.72 – 5.53 (m, 2H d_1), 5.46 (ddtd, *J* = 10.0, 4.5, 2.7, 1.6 Hz, 1H d_2), 4.16 (td, *J* = 8.8, 4.5 Hz, 1H d_1), 4.13 – 4.07 (m, 0.5H d_2), 4.02 – 3.95 (m, 1H d_1 + 0.5H d_2) , 3.11 – 3.05 (m, 1H d_1 + 0.5H d_2), 2.46 (dtd, *J* = 12.8, 8.3, 4.5 Hz, 1H d_1), 2.39 – 2.25 (m, 2H d_1 + 1.5H d_2), 2. 15 – 2.05 (m, 2H d_1), 1.92 – 1.86 (m, 0.5H d_2), 1.85 – 1.78 (m, 1H d_1 + 0.5H d_2), 1.77 (m, 0.5H d_2), 1.74 – 1.68 (m, 1H d_1), 1.68 – 1.62 (m, 1H d_1), 1.61 – 1.58 (m, 0.5H d_2), 0.99 – 0.93 (m, 0.5H d_2).

^{13}C NMR (151 MHz, CDCl$_3$) δ 141.9, 140.3, 128.8, 128.6, 128.4, 128.4, 127.3, 126.9, 126.7, 126.7, 126.5, 124.9, 124.8, 83.0, 82.3, 65.4, 65.4, 53.9, 53.9, 36.5, 32.8, 32.7, 32.1, 32.1, 29.9, 28.3, 23.6, 22.7.

HRMS (TOF MS LD+) $C_{15}H_{19}O$ [M+H]$^+$: found 215.1432, required 215.1430.

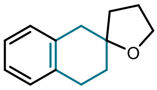

5aa

3',4,4',5-Tetrahydro-1'H,3H-spiro[furan-2,2'-naphthalene] (5aa)

Synthesized according to the general procedure **D** using 3-(naphthalen-2-yl)propan-1-ol (55.9 mg, 0.3 mmol) for the Birch reduction and TfOH (80 µL, 3 equiv.) in $CHCl_3$ (1.5 mL, 0.2 M) for the cyclization. The yield of **2aa** (50%) was determined by ^{1}H NMR analysis of the reaction crude after the Birch reduction. The crude mixture after the cyclization was purified by flash column chromatography on silica gel (hexane) to afford **5aa** as a colourless oil (22.6 mg, 40% yield over both steps).

^{1}H NMR (600 MHz, CDCl$_3$) δ 7.14 – 7.01 (m, 4H), 3.97 – 3.86 (m, 2H), 3.07 – 2.97 (m, 1H), 2.93 – 2.76 (m, 3H), 2.07 – 1.97 (m, 2H), 1.97 – 1.90 (m, 1H), 1.78 (t, *J* = 7.4 Hz, 3H).

^{13}C NMR (151 MHz, CDCl$_3$) δ 135.9, 135.5, 129.5, 128.8, 125.9, 125.8, 81.1, 67.2, 41.2, 36.0, 33.6, 27.6, 25.8.

HRMS (TOF MS LD+) $C_{13}H_{16}OK$[M+K]$^+$: found 227.0838, required 227.0833.

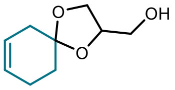

5y

(1,4-dioxaspiro[4.5]dec-7-en-2-yl)-Methanol (5y)

Synthesized according to the general procedure **D** using 3-Phenoxypropane-1,2-diol (50.5 mg, 0.3 mmol) for the Birch reduction and TFA (23 µL, 3 equiv.) in $CHCl_3$ (1.5 mL, 0.2 M) for the cyclization. The yield of **2y** (37%) was determined by ^{1}H NMR analysis of the reaction crude after the Birch reduction. The crude mixture after the cyclization was purified by flash column chromatography on silica gel (hexane/EtOAc 95:5) to afford **5y** in 62% yield, in a diastereomeric mixture (1:1) as a colourless oil (13.6 mg, 22% yield over both steps).

Diastereomeric mixture (1:1):

^{1}H NMR (600 MHz, CDCl$_3$) δ 5.75 – 5.67 (m, 2H, d_1+d_2), 5.64 – 5.58 (m, 2H, d_1+d_2), 4.33 – 4.23 (m, 2H, d_1+d_2), 4.08 (td, *J* = 8.2, 6.5 Hz, 2H, d_1+d_2), 3.85 (ddd, *J* = 8.2, 6.5, 4.5 Hz, 2H, d_1+d_2), 3.77 (dd, *J* = 11.7, 3.7 Hz, 2H, d_1+d_2), 3.61 (ddd, *J* = 11.8, 5.0, 0.6 Hz, 2H, d_1+d_2), 2.39 – 2.18 (m, 8H, d_1+d_2), 1.86 – 1.69 (m, 4H, d_1+d_2).

^{13}C NMR (151 MHz, CDCl$_3$) δ 126.4, 126.4, 124.0, 124.04 108.8, 108.7, 76.0, 75.9, 65.4, 65.4, 62.9, 62.8, 36.5, 35.4, 32.1, 30.5, 24.5, 24.4.

HRMS (TOF MS LD-) $C_9H_{13}O_3$ [M-]: found 169.0865, required 169.0870.

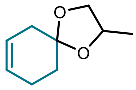

5ab

2-Methyl-1,4-dioxaspiro[4.5]dec-7-ene (5ab)

Synthesized according to the general procedure **D** using 1-phenoxy-2-propanol (45.7 mg, 0.3 mmol) for the Birch reduction and TFA (23 µL, 3 equiv.) in CHCl$_3$ (1.5 mL, 0.2 M) for the cyclization. The yield of **2ab** (56%) was determined by ^{1}H NMR analysis of the reaction crude after the Birch reduction. The crude mixture after the cyclization was purified by flash column chromatography on silica gel (hexane) to afford **5ab** in 57% yield, in a diastereomeric mixture (1:1) as a colourless oil (14.9 mg, 32% yield over both steps).

Diastereomeric mixture (1:1): **^{1}H NMR (600 MHz, CDCl$_3$)** δ 5.75 – 5.66 (m, 2H), 5.64 – 5.56 (m, 1H), 4.33 – 4.22 (m, 1H), 4.09 (ddd, *J* = 9.0, 7.9, 5.7 Hz, 1H), 3.48 (td, *J* = 7.8, 4.1 Hz, 1H), 2.35 – 2.20 (m, 4H), 1.82 – 1.73 (m, 2H), 1.30 (dd, *J* = 6.1, 2.0 Hz, 3H).

^{13}C NMR (151 MHz, CDCl$_3$) δ 126.5, 126.4, 124.3, 124.2, 108.1, 108.1, 72.0, 71.8, 70.6, 70.6, 36.0, 32.4, 31.1, 24.6, 24.4, 18.6, 18.5.

HRMS (TOF MS LD+) $C_9H_{14}O_2Na[M+Na]^+$: found 177.0891, required 177.0886.

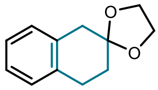

5s

3,4-Dihydro-1*H*-spiro[naphthalene-2,2'-[1,3]dioxolane] (5s)

Synthesized according to the general procedure **D** using 2-(naphthalen-2-yloxy)-ethanol (56.5 mg, 0.3 mmol) for the Birch reduction and TFA (23 µL, 3 equiv.) in CHCl$_3$ (1.5 mL, 0.2 M) for the cyclization. The yield of **2s** (68%) was determined by ^{1}H NMR analysis of the reaction crude after the Birch reduction. The crude mixture after the cyclization was purified by flash column chromatography on silica gel (hexane) to afford **5s** in 54% yield, as a colourless oil (24.7 mg, 37% yield over both steps).

^{1}H NMR (600 MHz, CDCl$_3$) δ 7.15 – 7.02 (m, 4H), 4.08 – 4.00 (m, 4H), 3.01 – 2.96 (m, 4H), 1.99 – 1.92 (m, 2H).

^{13}C NMR (151 MHz, CDCl$_3$) δ 135.2, 134.3, 129.2, 128.4, 126.0, 125.8, 108.2, 64.5, 39.0, 31.7, 29.6, 28.0.

HRMS (TOF MS LD+) $C_{12}H_{15}O_2[M+H]^+$: found 191.1065, required 191.1067.

1-Oxaspiro[5.5]undec-8-ene (5ac)

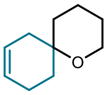
5ac

Synthesized according to the general procedure **E** using 4-phenylbutan-1-ol (30 mg, 0.2 mmol) for the Birch reduction, affording the product **2ac** (12.2 mg, 40%). For the cyclization (7.6 mg, 0.05 mmol, 1 equiv.) of **2ac** have been used. The yield of **5ac** (60%) was determined by ^{1}H NMR analysis of the reaction crude after cyclization reaction. Two proton peaks are missing due to overlap with residual solvent peak, which could not be completely removed due to the high volatility of **5ac**.

^{1}H NMR (400 MHz, $CDCl_3$) δ 5.66 -5.62 (m, 1H), 5.50 – 5.24 (m, 1H), 2.43 – 2.39 (m, 1H), 2.28 – 2.23 (m, 2H), 2.09 -2.03 (m, 1H), 1.93 – 1.91 (m, 2H), 1.77 – 1.65 (m, 2H), 1.62 – 1.54 (m, 2H), 1.44 – 1.36 (m, 2H).

^{13}C NMR (151 MHz, $CDCl_3$) δ 126.0, 123.2, 72.1, 62.4, 43.7, 39.7, 35.4, 23.1, 20.1, 18.9.

HRMS (TOF MS LD+) $C_{10}H_{16}O[M+H]^+$: found 153.1278, required 153.1201.

7-Oxaspiro[5.6]dodec-2-ene (5ad)

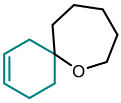
5ad

Synthesized according to the general procedure **E** using 5-phenylpentan-1-ol (33. 2 mg, 0.2 mmol) for the Birch reduction, affording the product **2ad** (15.0 mg, 45%). For the cyclization (8.3 mg, 0.05 mmol, 1 equiv.), of **2ad** have been used. The yield of **5ad** (32%) was determined by ^{1}H NMR analysis of the reaction crude after cyclization reaction. Two proton peaks are missing due to overlap with residual solvent peak, which could not be completely removed due to the high volatility of **5ad**.

^{1}H NMR (400 MHz, $CDCl_3$) δ 5.73 – 5.67 (m, 1H), 5.57 – 5.50 (m, 1H), 2.51 – 2.40 (m, 1H), 2.34 – 2.25 (m, 2H), 2.09 – 1.99 (m, 1H), 1.78 – 1.67 (m, 2H), 1.60 – 1.49 (m, 4H), 1.38 – 1.27 (m, 3H)

^{13}C NMR (151 MHz, $CDCl_3$) δ 126.1, 123.4, 72.4, 62.7, 44.0, 39.8, 35.5, 25.8, 23.6, 23.2, 18.9.

HRMS (TOF MS LD+) $C_{11}H_{18}O[M+H]^+$: found 167.1435, required 167.1358.

E.4. Procedure for the scale up in batch

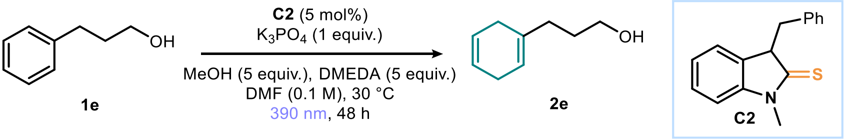

Catalyst **C2** (44.3 mg, 0.175 mmol, 0.05 equiv.) and K_3PO_4 as powder (743 mg, 3.5 mmol, 1 equiv.) were added sequentially to a 100 mL flask containing a stirring bar. The flask was

sealed with a septum and then three times vacuumed for 3 minutes and backfilled with argon. Oxygen-free DMF (35 mL, 0.1 M) was added and the solution was stirred until the catalyst was completely dissolved. Then, MeOH (710 µL, 17.5 mmol, 5 equiv.), 3-phenylpropan-1-ol 1 e (477 mg, 3.5 mmol, 1 equiv.) and DMEDA (1.89 mL, 17.5 mmol, 5 equiv.) were added via syringe. The flask was placed in the photoreactor (Figure S6) and stirred (600 rpm) at 30 ºC (reactor temperature) under 390 nm irradiation (2x PR160L-390 nm, 100% light intensity) for 48 hours. After the reaction was completed, the mixture was filtered and then the solvent was removed under reduced pressure (40 °C, < 15 mbar). The crude residue was purified by flash column chromatography using silica gel impregnated with 2% w/w $AgNO_3$ (hexane/AcOEt, 2:1) to afford **2e** as a yellow liquid (242 mg, 50% yield).

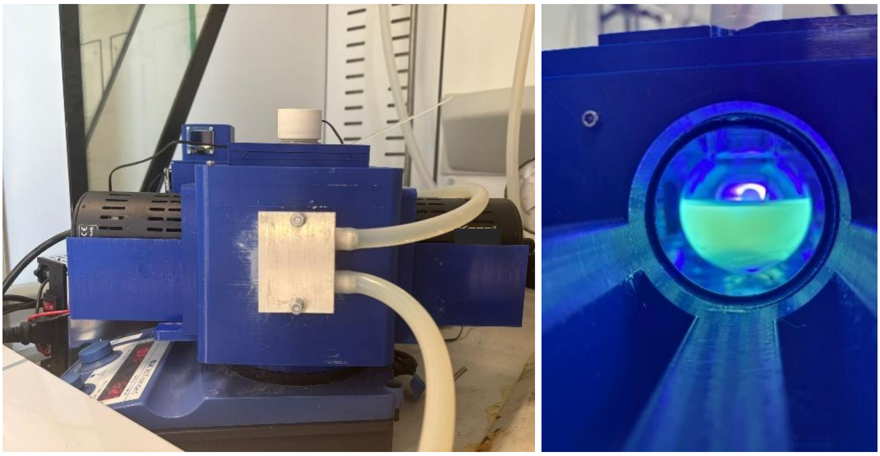

Figure S6. *(left)* Batch-scale reaction setup using a 3D-printed, temperature controlled photoreactor[3], illuminated at 390 nm with two Kessil lamps. *(right)* View of the reaction flask in the reactor

E.5. Procedure for the flow-scale up

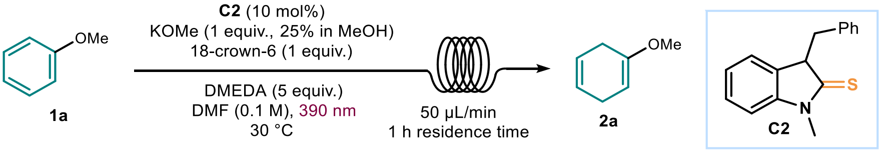

18-crown-6 (1 equiv.) was added to a flask containing a stirring bar and dried under vacuum and heat. Then catalyst **C2** (0.1 equiv.) was added and the flask was sealed with a septum and then three times vacuumed for 10 minutes and backfilled with argon. KOMe (25% solution in MeOH, 1 equiv.) and oxygen-free DMF (0.1 M) were added, and the mixture was stirred until complete dissolution of the catalyst was observed. Then, anisole (1 equiv.), DMEDA (5 equiv.), and 1,3,5-triisopropylbenzene as the internal standard (0.167 equiv.) were added via

syringe. The solution was taken up into a syringe and connected to the 3D-printed flow unit equipped with a 6 m PFA coil (0.8 mm internal diameter, Figure S7). The reaction solution was pumped using a syringe pump (Legato® 100 from KD Scientific) at a flow rate of 50 µl/min through the photoreactor at 30 ºC (reactor temperature) under 390 nm irradiation (2x PR160L-390 nm, 100% light intensity). The yield of **2a** (47% yield) was determined via ^{1}H NMR analysis of the crude mixture.

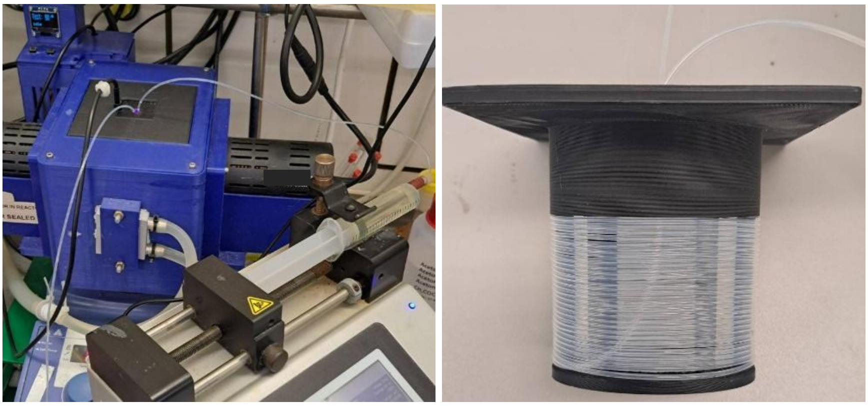

Figure S7. *(left)* Continuous flow reaction setup using a temperature-controlled 3D-printed photoreactor[3] under illumination at 390 nm by two Kessil lamps. *(right)* 3D-printed flow unit using a PFA coil (ID 0.8 mm, length 6 m).

F. Mechanistic Studies

F.1. Deprotonation Experiments

To a 7 mL glass vial were added catalyst **C2** (0.2 mmol, 1 equiv.) and K_3PO_4 (0.4 mmol, 2 equiv.). The vial was sealed with a screw-cap septum, evacuated and backfilled with argon three times, and DMF-d_7 was added via syringe. The mixture was stirred at ambient temperature for 5 min, then transferred under argon into an NMR tube via syringe, and the ^{1}H NMR spectrum was recorded (Figure S8). Complete and rapid deprotonation of **C2** was observed.

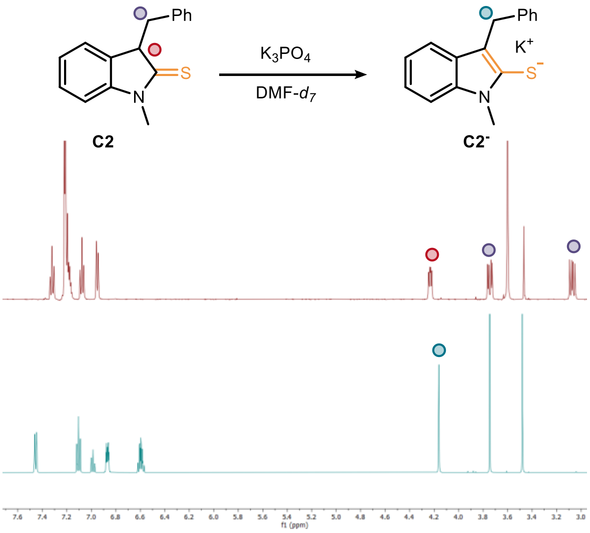

Figure S8. ^{1}H NMR analysis of catalyst **C2** before (brown spectrum) and after treatment with K_3PO_4 (blue) in DMF-*d*$_7$.

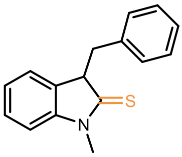

C2

3-Benzyl-1-methylindoline-2-thione (C2) in DMF-*d*$_7$

^{1}H NMR (500 MHz, DMf-*d*$_7$) δ 7.32 (t, *J* = 7.7 Hz, 1H), 7.28 – 7.15 (m, 6H), 7.07 (t, 1H), 6.95 (d, *J* = 7.4 Hz, 1H), 4.23 (dd, *J* = 8.2, 5.0 Hz, 1H), 3.74 (dd, *J* = 13.7, 5.0 Hz, 1H), 3.60 (s, 3H), 3.07 (dd, *J* = 13.6, 8.2 Hz, 1H).

^{13}C NMR (126 MHz, DMF-*d*$_7$) δ 145.72, 136.86, 130.42, 128.93, 127.52, 124.53, 116.91, 114.89, 113.02, 107.81, 105.92, 32.91.

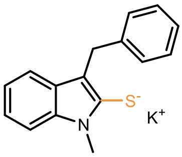

C2$^-$

Potassium 3-benzyl-1-methyl-1*H*-indole-2-thiolate (C2$^-$) in DMF-*d*$_7$

^{1}H NMR (500 MHz, DMF-*d*$_7$) δ 7.45 (d, 2H), 7.10 (t, *J* = 7.6 Hz, 2Hf), 6.99 (t, 1H), 6.91 – 6.79 (m, 2H), 6.67 – 6.52 (m, 2H), 4.16 (s, 2H), 3.75 (s, 3H).

^{13}C NMR (126 MHz, DMSO-*d*$_6$) δ 157.45, 145.72, 136.86, 130.42, 128.93, 127.52, 124.53, 116.91, 114.89, 113.02, 107.81, 105.92, 32.91.

F.2. Catalyst Stability Studies

F.2.1. C1 and C2 catalysts stability studies under aerobic conditions

The chemical stability of the deprotonated catalyst **C2**$^-$ was evaluated in comparison to the previously reported catalyst **C1**$^-$ under conditions of moisture and air exposure. Solutions of each catalyst (0.1 mmol, 1 equiv.) with potassium phosphate tribasic (0.2 mmol, 2 equiv., to ensure deprotonation) were prepared in DMF-d_7 (0.5 mL) in separate NMR tubes. The solutions were bubbled with air for 15 min, and ^{1}H NMR spectra were recorded. The same samples were then irradiated at 390 nm for 30 min in the NMR tubes, followed by additional ^{1}H NMR analysis.

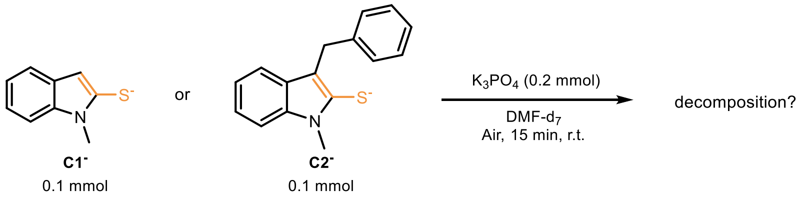

Stability test for C1$^-$: Exposure to air led to clear signs of degradation and upon subsequent irradiation further unidentified degradation products were formed (Figure S9).

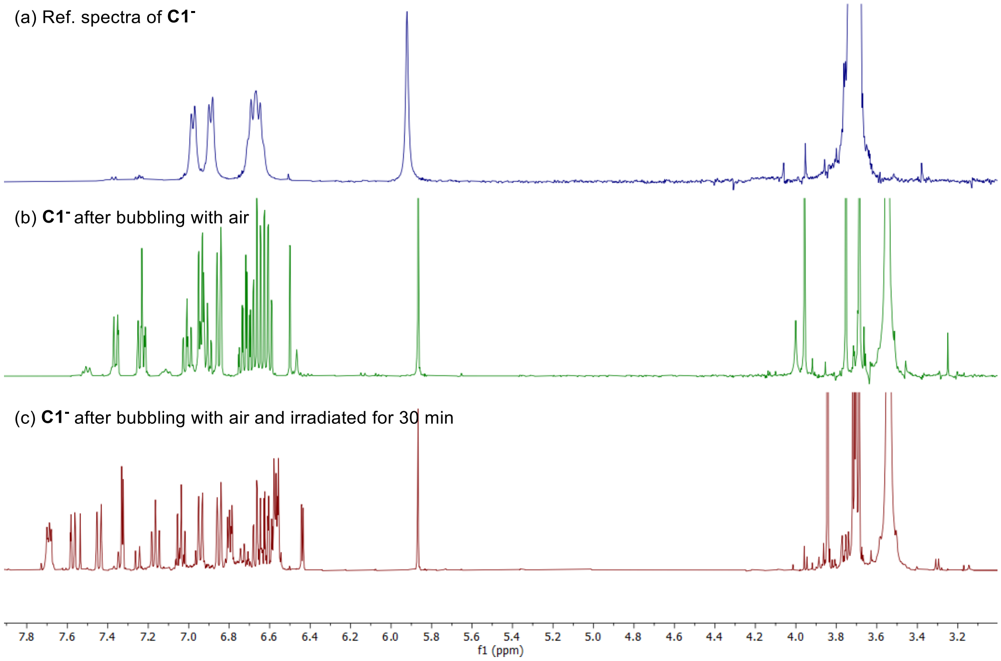

Figure S9. Stability of catalyst **C1**$^-$ under aerobic conditions, before and after irradiation in DMF-d_7.

Stability test for C2$^-$**:** In contrast, **C2**$^-$ exhibited no significant degradation upon exposure to air (Figure S10). More decompostion occured after irradiation at 390 nm for 30 min but a

siginificant amount of catalyst **C2**$^-$ remained, indicating significantly enhanced chemical stability and robustness toward oxygen.

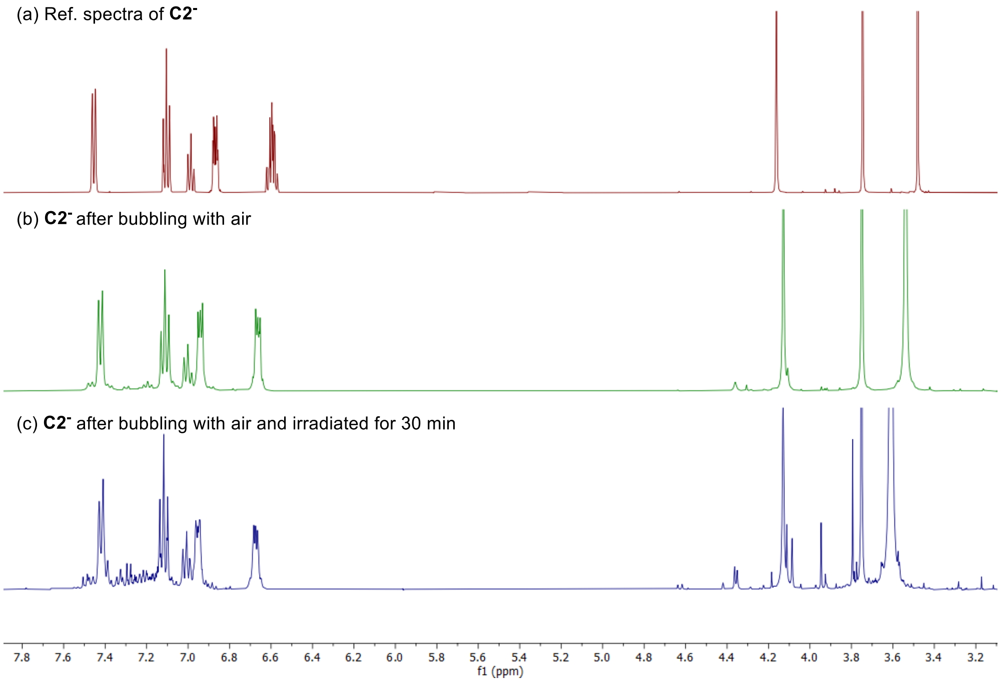

Figure S10. Stability of catalyst **C2**$^-$ under aerobic conditions, before and after irradiation in DMF-d_7.

F.2.2. Catalysts stability studies under the reaction conditions

C2 stability evaluation under catalytic conditions

The stability of catalyst **C2** during the reaction was monitored over time by NMR and HPLC–MS for the model Birch reduction of anisole **1a** (Figure S11). The reaction was carried out according to the general procedure on a 0.1 mmol scale. Separate reaction mixtures were prepared for each data point, followed by the addition of dibromomethane as an internal standard. Each aliquot was analyzed by NMR after dilution with DMSO-d_6 to determine the amount of recovered catalyst.

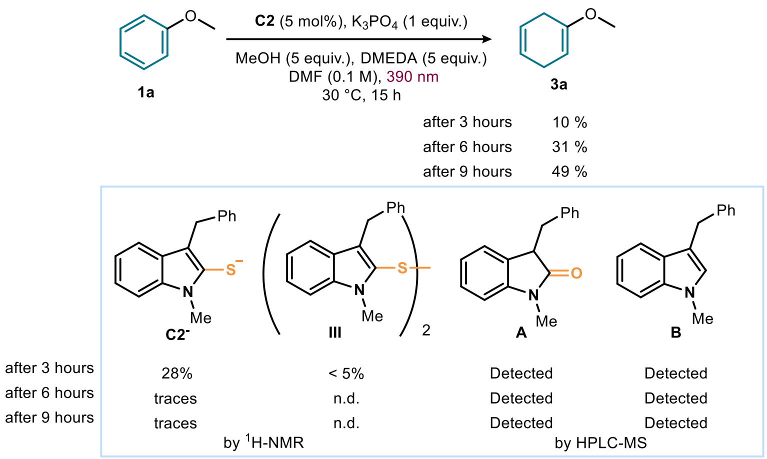

Figure S11. Degradation profiles of catalysts **C2** during the Birch reduction of **1a**.

It was found that deprotonated active species **C2**⁻ is present in 28% after 3 h, together with <5% of the S–S dimer. **C2**⁻ remains detectable also after 9 h. Degradation products **A** and **B** were identified by HPLC–MS using independently synthesized standards (Figure S12), and *neither promotes the reduction of anisole*, which excludes them as catalytically relevant intermediates.

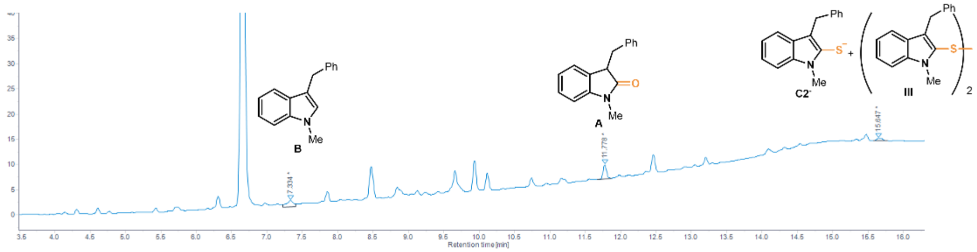

Figure S12. HPLC–MS trace of the crude reaction mixture at 6 h for the Birch reduction of anisole **1a** with **C2**.

C3 stability evaluation under catalytic conditions

A similar degradation monitoring was carried out using catalyst **C3** for the reduction of benzofuran (**3h**, Figure S13). **C3** remains largely intact (56% after 3 h; 20% after 9 h).

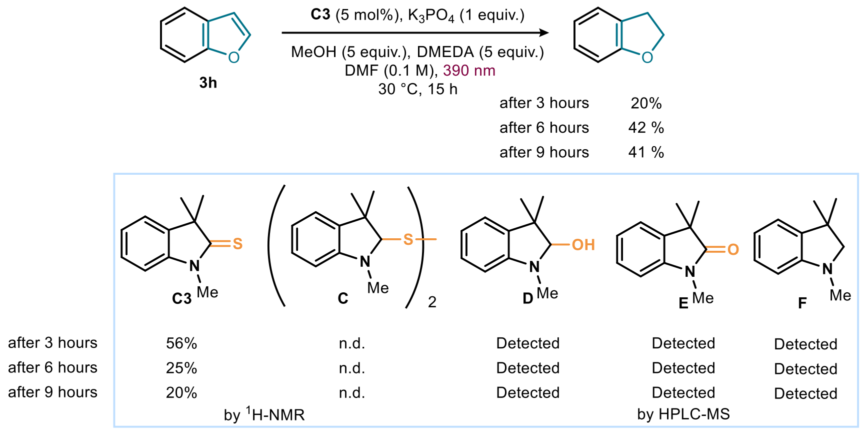

Figure S13. Degradation profiles of catalysts **C3** during the Birch reduction of **3h**.

Three degradation products (**D, E, F**) were identified by HPLC–MS based on authentic samples. Importantly, **D** and **E** do not catalyze the photoreduction of benzofuran under our conditions (Figure S14). Moreover, no S–S dimer analogous to that observed for **C2** was detected for **C3**.

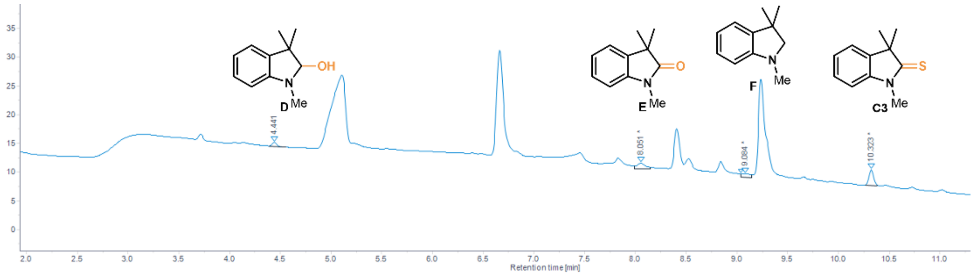

Figure S14. HPLC–MS trace of the crude reaction mixture at 6 h for the Birch reduction of benzofuran **3h** with **C3**.

F.3. Photophysical Studies

Sample Preparation:

- Neutral catalysts **C2**

A 25 mL glass vial containing catalyst **C2** (0.01 mmol) was sealed with a septum, vacuumed and backfilled with argon for 3 times, then degassed DMF (5 mL, HPLC grade) was added *via* syringe ($2\cdot10^{-3}$ M solution). 40 µL of the stock solution were taken and diluted further with DMF (4 mL) to obtain a $2\cdot10^{-5}$ M solution (20 µL were taken for a $1\cdot10^{-5}$ M solution). 3 mL of the solution was transferred into an argon filled quartz cuvette (10 x 10 mm light path) equipped with a septum.

- Deprotonated catalysts **C2**$^{-}$

A 25 mL glass vial containing K_3PO_4 (130 mg) was sealed with a septum, vacuumed and backfilled with argon for 3 times, then degassed DMF (16 mL, HPLC grade) was added *via* syringe. Addition of 160 µL of a freshly prepared neutral catalyst **C2** solution ($2\cdot10^{-3}$ M) gave a $2\cdot10^{-5}$ M solution (80 µL were taken for a $1\cdot10^{-5}$ M solution). The vial was sealed with Parafilm and stirred at ambient temperature for 0.5 hour. This solution was first filtered through an HPLC grade 25 mm syringe-filter into another 25 mL glass vial (preventively vacuumed and backfilled with argon for 3 times). Then, 3 mL of the filtered solution were transferred into an argon filled quartz cuvette (10 x 10 mm light path) equipped with a septum.

- Catalyst **C3**

A 25 mL glass vial containing catalyst **C3** (0.01 mmol) was sealed with a septum, vacuumed and backfilled with argon for 3 times, then degassed DMF (5 mL, HPLC grade) was added *via* syringe ($2\cdot10^{-3}$ M solution). 200 µL of the stock solution were taken and diluted further with DMF (4 mL) to obtain a $1\cdot10^{-4}$ M solution. 3 mL of the solution were transferred into an argon filled quartz cuvette (10 x 10 mm light path) equipped with a septum.

- Catalyst **C3 +** DMEDA + K_3PO_4 solution

*The stoichiometry of DMEDA and K_3PO_4 with respect to **C3** was chosen from the optimized reaction conditions for the photo-Birch (5 mol% of catalyst, 100 mol% of K_3PO_4 and 500 mol% of DMEDA).*

A 25 mL glass vial containing catalyst **C3** (0.01 mmol) and K_3PO_4 (0.2 mmol) was sealed with a septum, vacuumed and backfilled with argon for 3 times, then degassed DMF (5 mL, HPLC grade) and DMEDA (1.0 mmol) were added *via* syringe ($2\cdot10^{-3}$ M solution of **C3**). This solution was first filtered through an HPLC grade 25 mm syringe-filter into another 25 mL glass vial (preventively vacuumed and backfilled with argon for 3 times). Then, 200 µL of the stock solution were taken and diluted further with DMF (4 mL) to obtain a $1\cdot10^{-4}$ M solution of **C3**. 3 mL of the solution were transferred into an argon filled quartz cuvette (10 x 10 mm light path) equipped with a septum.

F.3.1. Absorption measurements

F.3.1.1. Absorption spectra of catalyst C2

UV-Vis measurements were carried out on an Agilent Cary 60 UV-Vis spectrophotometer equipped with two silicon diode detectors, double beam optics and Xenon pulse light. The absorption spectra of the neutral catalyst **C2** and the deprotonated catalyst **C2**$^-$ was recorded (Figure S15).

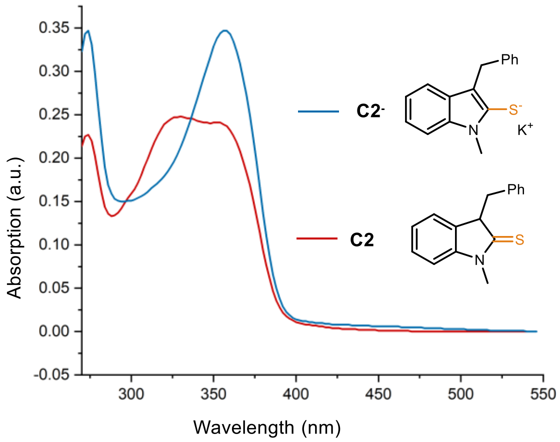

Figure S15. Absorption spectra recorded for catalyst **C2** in DMF without (red line) and with (blue line) K_3PO_4 in a 2 ·10^{-5} M solution.

F.3.1.2 Absorption spectra of catalyst C3

UV-Vis measurements were carried out on an Agilent Cary 60 UV-Vis spectrophotometer equipped with two silicon diode detectors, double beam optics and Xenon pulse light. The absorption spectra of the catalyst **C3** and the solution containing catalyst **C3** in the presence of DMEDA and K_3PO_4 were recorded (Figure S16). No major differences in the absorption spectra of the two solutions were identified.

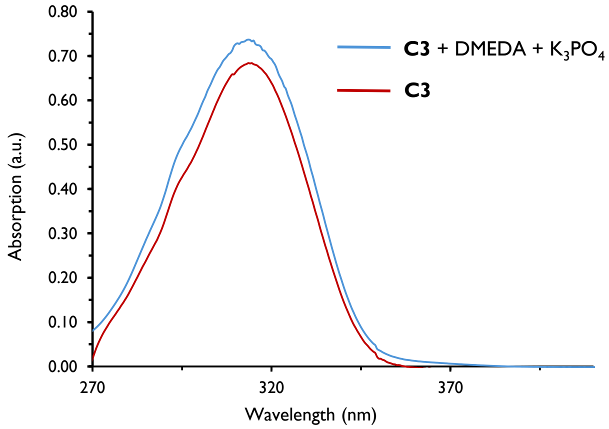

Figure S16. Absorption spectra recorded for catalyst **C3** in DMF without (red line) and with (blue line) DMEDA and K_3PO_4 in a 1 ·10^{-4} M solution.

F.3.2. Emission Studies

F.3.2.1. Emission spectra of Deprotonated C2

Fluorescence measurements were carried out on an Agilent Cary Eclipse fluorescence spectrometer equipped with a high voltage PMT detector and continuum Xe light source. The emission spectrum of the deprotonated catalyst **C2**$^-$ (formed in situ upon addition of K_3PO_4 in degassed DMF according to the standard procedure) was recorded from 370 nm to 600 nm after excitation with a 355 nm wavelength (Figure S17).

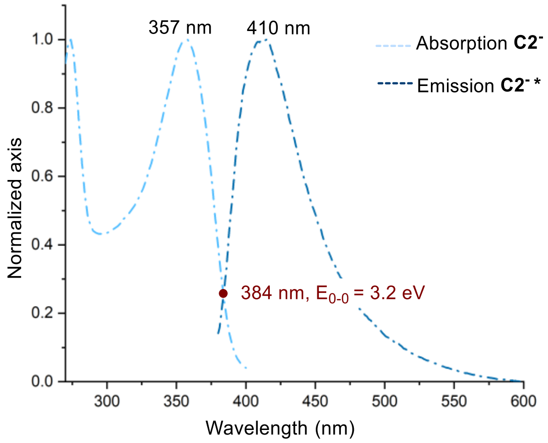

Figure S17. Normalized absorption and emission spectra of the active catalyst **C2**$^-$ (formed upon deprotonation of **C2**) in DMF in a $2 \cdot 10^{-5}$ M solution.

F.3.2.2. Emission Spectra of Catalyst C3

Fluorescence measurements were carried out on an Agilent Cary Eclipse fluorescence spectrometer equipped with a high voltage PMT detector and continuum Xe light source. The emission spectra of the catalyst **C3** was recorded from 306 nm to 500 nm after excitation with a 296 nm wavelength (Figure S18).

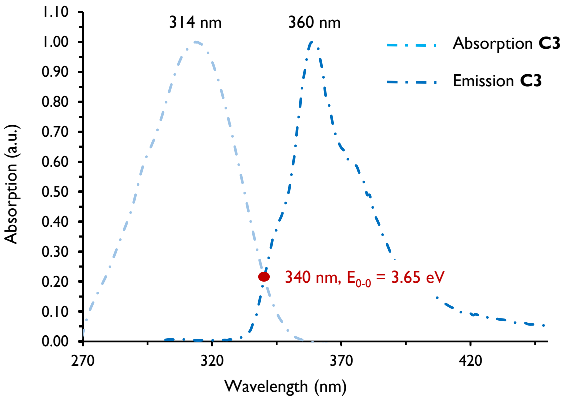

Figure S18. Normalized absorption and emission spectra of the catalyst **C3** in DMF in a $1 \cdot 10^{-4}$ M solution.

F.3.3. Stern-Volmer Quenching Studies

F.3.3.1. Stern-Volmer Quenching Studies of Deprotonated Catalyst C2

Fluorescence measurements were carried out on an Agilent Cary Eclipse fluorescence spectrometer equipped with a high voltage PMT detector and continuum Xe light source. A 1.0 M solution of anisole **1a** (the quencher substrate) in degassed DMF was prepared and 20 μL of this stock solution were added to the solution of the active catalyst **C2**$^{-}$, prepared according to the standard procedure. The addition of the anisole solution (the quencher) was repeated for five times (Figure S19). After each addition, the solution was mixed and the emission spectra of the excited catalyst was acquired from 380 nm to 600 nm (the excitation wavelength was fixed at 370 nm). A solvent blank was subtracted from all the measurements.

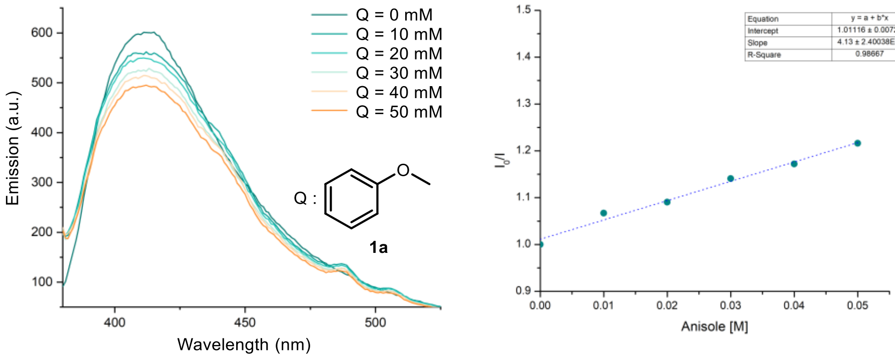

Figure 19. Stern-Volmer quenching studies of the deprotonated catalyst **C2** with anisole **1a**.

The Stern-Volmer plot shows a linear correlation between the amounts of anisole **1a** and the ratio I_0/I, following the relationship: $I_0/I = 1 + K_{SV}[Q]$ (Q = Quencher).

For **1a** as quencher, a Stern-Volmer quenching constant of 4.13 M^{-1} was calculated.

F.3.3.2. Stern-Volmer Quenching Studies of Catalyst C3

Fluorescence measurements were carried out on an Agilent Cary Eclipse fluorescence spectrometer equipped with a high voltage PMT detector and continuum Xe light source. A 1.0 M solution of DMEDA + 0.2 M K_3PO_4 (filtered before addition) was used as the quencher solution. The solution was prepared in degassed DMF. 30 μL of this stock solution were added to the solution of the catalyst **C3**, prepared according to the standard procedure ($1\cdot10^{-4}$ M). The addition of this solution was repeated for five times (Figure S20). After each addition, the solution was quickly mixed, and the emission spectra of the excited catalyst was acquired from 306 nm to 500 nm (the excitation wavelength was fixed at 296 nm). A solvent blank was subtracted from all the measurements.

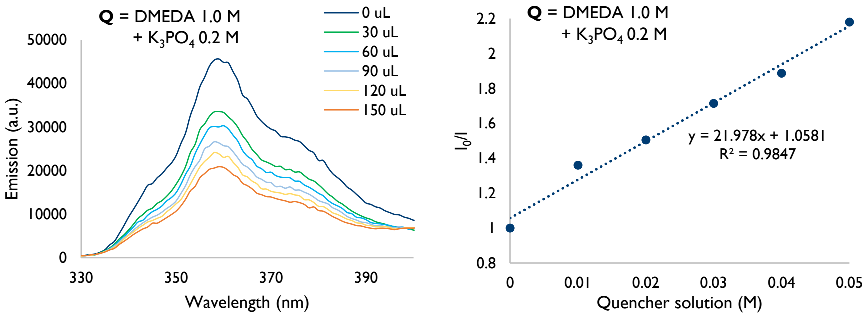

Figure S20. Stern-Volmer quenching studies of the catalyst **C3** with a solution of 1.0 M DMEDA and 0.2 M K_3PO_4 in degassed DMF.

The Stern-Volmer plot shows a linear correlation between the amounts of the quencher solution and the ratio I_0/I, following the relationship: $I_0/I = 1 + K_{SV}[Q]$ (Q = Quencher).

For the solution containing both DMEDA and K_3PO_4, a Stern-Volmer quenching constant of 21.98 M^{-1} was calculated.

F.3.4. Lifetime Measurements

F.3.4.1. Lifetime Measurement of Deprotonated Catalyst C2

The emission lifetime (τ) in the nanoseconds range was determined via single photon counting technique (TCSPC) with Edinburgh FLSP920 spectrometer using pulsed picosecond LED (EPLED 365, FWHM < 800ps) as the excitation source. The detector was the R928P PMT. The goodness of fit was assessed by minimizing the reduced χ^2 function and by visual inspection of the weighted residuals. The lifetime was determined to be 0.8 ns (Figure S21).

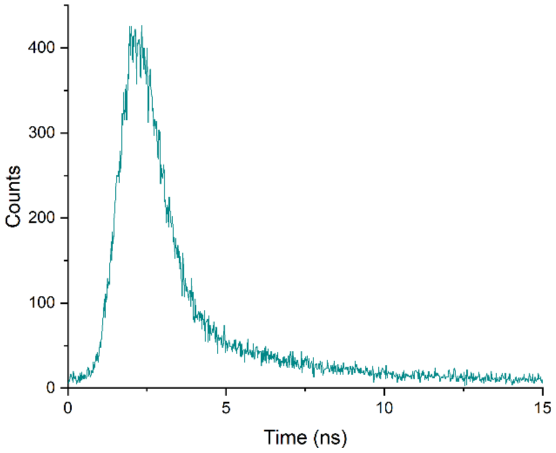

Figure S21. Excited state decay of **C2**⁻ in DMF.

Table 1. Tables of important photophysical parameters

	Abs λ_{max} / nm	Em λ_{max} / nm	τ S_1 / ns	$\Delta\lambda_{abs-em}$ / nm	S_1 / kcal/mol (eV)	ε390 / $M^{-1}cm^{-1}$
**$C2^-$**	357	410	0.8	53	73.8 (3.2)	2175

Quenching studies were conducted to determine whether static or dynamic quenching occurred during the reaction by measuring the catalyst lifetime after several additions of the quencher (anisole). From the plot, it can be determined that we are in a dynamic quenching regime because the catalyst lifetime decreases as the quencher concentration increases (Figure S22).

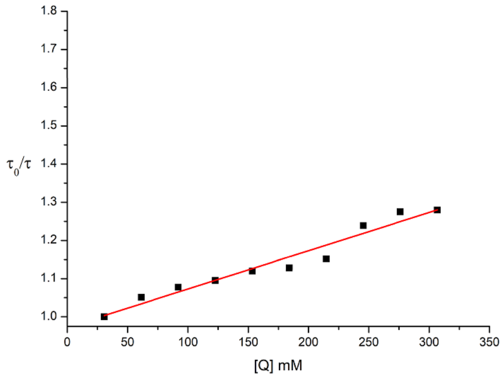

Figure S22. Dynamic quenching studies of **$C2^-$** using anisole as quencher.

F.3.4.2. Lifetime Measurement of Catalyst C3

The emission lifetime (τ) in the nanoseconds range was determined via single photon counting technique (TCSPC) with Edinburgh FLSP920 spectrometer using pulsed picosecond LED (EPLED 295, FWHM < 800ps) as the excitation source. The detector was the R928P PMT. The goodness of fit was assessed by minimizing the reduced χ^2 function and by visual inspection of the weighted residuals. The lifetime of **C3** was determined to be 7.44 ns (Figure S23). When the same measurement was repeated on the solution containing **C3**, DMEDA and K_3PO_4 (prepared according to the general procedure) the fitted lifetime dropped to 2.28 ns, further corroborating the hypothesis that DMEDA actively quenches the excited state of **C3**.

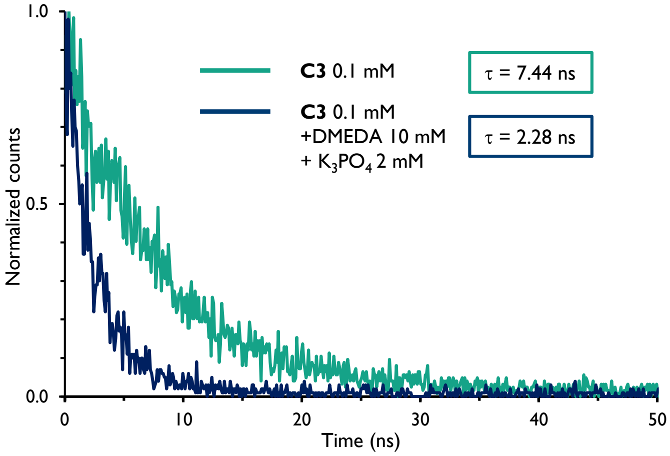

Figure S23. Excited state decay of **C3** (1 x 10^{-4} M) in DMF (green line); Excited state decay of **C3** (1 x 10^{-4} M) in the presence of DMEDA (10 mM) and K_3PO_4 (2 mM) in DMF (dark blue line).

F.4. Electrochemical Studies

Cyclic voltammetry (CV) measurements were carried out on a Autolab PGSTAT204potentiostat with a glassy carbon disk electrode (diameter: 3 mm) as working electrode. A silver wire coated with AgCl immersed in a 3.0 M aqueous solution of KCl and separated from the analyte by a fritted glass disk was employed as the reference electrode and a Pt wire counter-electrode completed the electrochemical setup. The scan rate was 100 mV/s unless otherwise stated. The substrates were measured at concentration of 0.01 M in DMF with $TBAPF_6$ (0.1 M) as electrolyte. The preparation of the catalysts solutions was carried out as described in the photophysics studies section (at a concentration of 0.01 M).

Potentials are quoted with the following notation: E_p^C (E_{Red}) refers to the cathodic peak potential, E_p^A (E_{Ox}) refers to the anodic peak potential.

F.4.1. Cyclic voltammetry measurements

F.4.1.1. Cyclic voltammetry of deprotonated C2 and C2 dimer

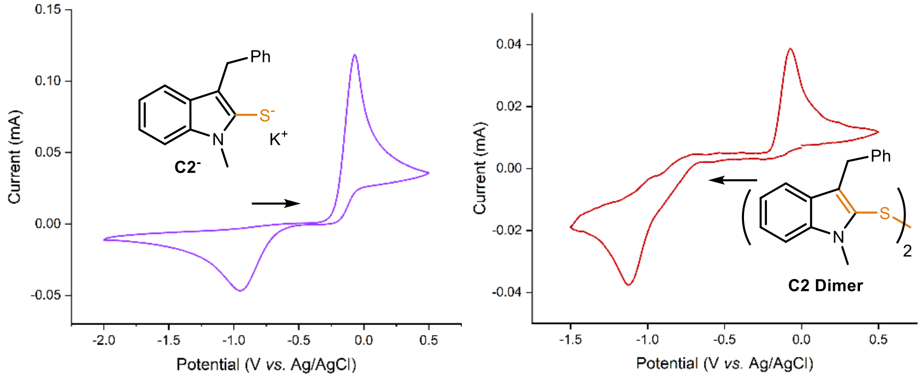

Figure S24. (*left*) CV of the deprotonated catalyst **C2**⁻ in DMF starting with the oxidation, irreversible oxidation E_p^A = -0.07 V, and irreversible reduction E_p^C = -0.95 V *vs.* Ag/AgCl. (*right*) CV of the Dimer of catalyst **C2** in DMF starting with the reduction, irreversible reduction E_p^C = -1.12 V, and irreversible oxidation E_p^A = -0.07 V *vs.* Ag/AgCl.

F.4.1.2. Cyclic voltammetry of C3 and benzofuran

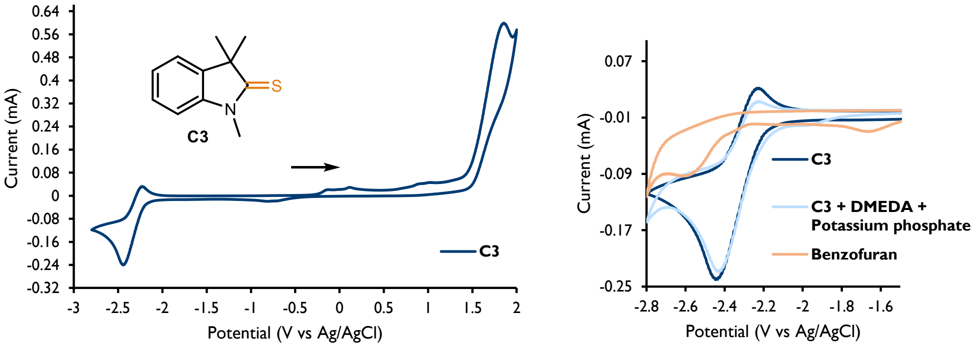

Figure S25. (*left*) CV of the catalyst **C3** in DMF (10 mM), irreversible reduction E_p^C = -2.44 V, *vs.* Ag/AgCl. (*right*) Zoom on CV comparison of **C3** (10 mM) in DMF (dark blue line); **C3** (10 mM) + DMEDA (1.0 M) and K_3PO_4 (0.2 M) in DMF (light blue line); Benzofuran (10 mM; light orange line) in DMF with a irreversible reduction E_p^C = -2.59 V, *vs.* Ag/AgCl.

F.4.2. Conversion of the potential from Ag/AgCl to SCE

The conversion of the redox potential from Ag/AgCl to SCE was done according to the literature by measuring the redox potential of ferrocene as reference in CH_3CN.[13]

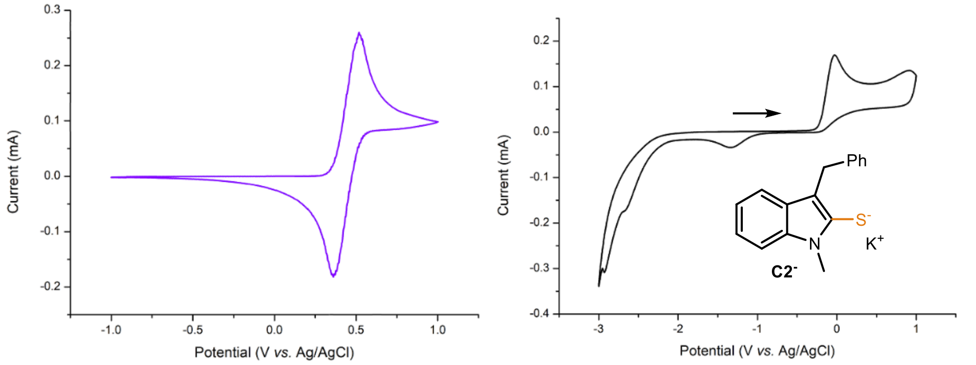

Figure S26. (*left*) CV of ferrocene in CH_3CN, reversible reduction and oxidation $E_{1/2}$ = 0.435 V. (right) CV of catalyst **C2**⁻ in CH_3CN, irreversible oxidation $E_p{}^A$ = -0.027 V.

With the reference CV, the redox potential vs. SCE in CH_3CN was calculated using the following equations:

$$Ep^{A}\text{ (Ag/AgCl to Fc/Fc}^{+}\text{)} = -0.027 - 0.435 = -0.462 \text{ V } vs. \text{ Fc/Fc}^{+} \text{ in ACN}$$

$$Ep^{A}\text{ (Ag/AgCl to SCE)} = (-0.462) + 0.38 = -0.082 \text{ V } vs. \text{ SCE in ACN}$$

F.5. Evaluation of the Excited-State Potential of the Deprotonated Catalyst C2⁻

Using the data collected from the CV studies (Figure S26) and from the absorption and emission spectra (Figure S17) of the deprotonated catalyst **C2** (anion **C2**⁻) , we could estimate the redox potential of the excited state with the following Equation:[14]

$$E(\mathbf{Pc^{\bullet}/Pc^{-*}}) = E(\mathbf{Pc^{\bullet}/Pc^{-}}) - E_{0\text{-}0}(\mathbf{Pc^{-*}})/(\mathbf{Pc^{-}})$$

Since the electrochemical oxidation of **C2**⁻ is irreversible (Figure S24), the irreversible peak potential E_p anode was used for $E(Pc^{\bullet}/Pc^{-})$. The oxidation potential was calculated to be -0.07 V *vs.* SCE (in DMF). $E_{0\text{-}0}(Pc^{-*})/(Pc^{-})$ was determined spectroscopically from the intersection of the normalized absorbance and emission spectra to have a value of 3.31 eV.

The redox potential of excited-state **C2**⁻:

$$E(\mathbf{Pc^{\bullet}/Pc^{-*}}) = (-0.07) - 3.23 = \mathbf{\mathit{-\ 3.30\ V\ vs.\ Ag/AgCl\ in\ DMF}}$$

$$E(\mathbf{Pc^{\bullet}/Pc^{-*}}) = (-0.027) - 3.23 = \mathbf{\mathit{-\ 3.26\ V\ vs.\ Ag/AgCl\ in\ ACN}}$$

$$E(\mathbf{Pc^{\bullet}/Pc^{-*}}) = (-0.082) - 3.23 = \mathbf{\mathit{-\ 3.31\ V\ vs.\ SCE\ in\ ACN}}$$

F.6. Evidence of Hydrogen Bonding Interaction Between DMEDA and K_3PO_4

To a 7 mL glass vial, DMEDA (0.1 mmol, 11 µL, 1 equiv.) and K_3PO_4 (0.1 mmol, 21 mg, 1 equiv.) and DMSO-d_6 (1 mL) were added. The solution was stirred at ambient temperature for 15 min and then the ^{1}H NMR was recorded (Figure S27). Upon the addition of K_3PO_4, the symmetric peak signals of DMEDA split into two signals, likely due to hydrogen bonding to one N−H, leading to the desymmetrization of the molecule.

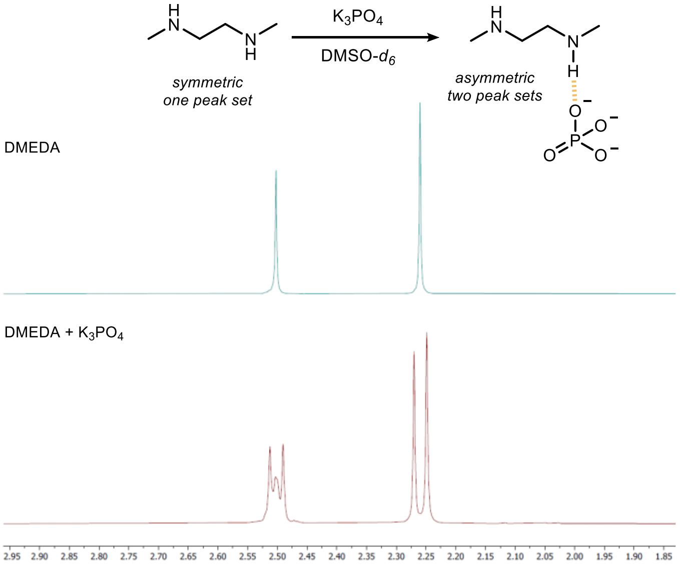

Figure S27. Hydrogen bonding interaction between DMEDA and K_3PO_4.

F.7. Turnover studies with C2 S−S dimer

To evaluate whether which reagent is responsible for the turnover of the catalyst, the S−S dimer **III** (0.01 mmol) of catalyst **C2** was irradiated in DMF (0.5 mL) in the presence and absence of DMEDA (1 mmol) and K_3PO_4 (0.2 mmol; 390 nm LEDs, 10 min, 30 °C in the photoreactor, Figure S28). The reactions were conducted according to the general procedure **B**; after 10 minutes, an internal standard was added and the sample transferred into an NMR tube.

Under the reaction conditions (with DMEDA and K_3PO_4), the deprotonated species **C2**$^-$ was detected with a 95% yield by ^{1}H NMR. Whereas in the absence of DMEDA, **C2**$^-$ was not observed and in the absence of K_3PO_4 no peaks corresponding to the neutral catalyst **C2** were detected. This confirms that both DMEDA and K_3PO_4 are required for catalyst turnover.

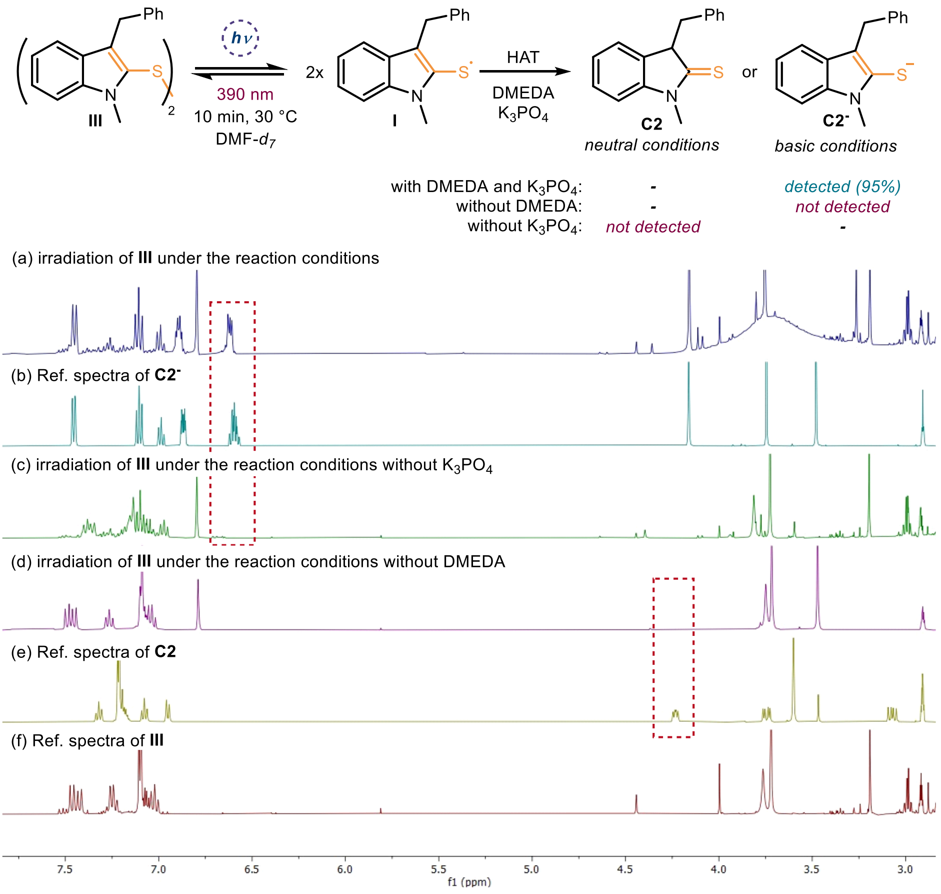

Figure S28. Turnover studies by irradiation of **III** under the reaction conditions, with and without base and DMEDA, measured in DMF-d_7.

F.8. Proposed Mechanism for C3

The combined photophysical, electrochemical, and reactivity studies presented above support the conclusion that **C2** and **C3** follow fundamentally different activation mechanisms under the reaction conditions. As established in Sections F.2–F.7, **C2** is readily deprotonated by K_3PO_4 to generate the thiolate **C2**$^-$, whose spectroscopic properties and electrochemical profile are fully consistent with the strongly reducing SET manifold discussed in the main manuscript (see Figure 6a).

In contrast, **C3** cannot be deprotonated, yet it remains an efficient photocatalyst for the reduction of heteroarenes such as benzofuran. The photophysical analysis clarifies the origin of this behaviour. The UV–Vis spectra of **C3**, recorded alone or in the presence of DMEDA and K_3PO_4, showed no detectable spectral shifts, ruling out aggregate or exciplex formation (Figure S16). Stern–Volmer quenching studies with DMEDA revealed a clean linear response (K_{SV} = 21.98 M^{-1}; Figure S20), consistent with productive excited-state quenching (via either

HAT or SET pathways). Cyclic voltammetry established the reduction potential of the **C3/C3**$^{\bullet-}$ couple as –2.44 V vs Ag/AgCl in DMF (Figure S25). Taken together, these data support the mechanistic picture in Figure 29a: the excited state of **C3** is quenched by DMEDA, generating the radical anion **C3**$^{\bullet-}$, which is sufficiently reducing to engage in SET to benzofuran (E^{p}_{red} = -2.59 V vs Ag/AgCl).

Further support for the intermediacy of **C3**$^{\bullet-}$ comes from a radical-trapping experiment with 3-butenylbenzene, which affords the C–C adduct **C3-I** (Figure S29b).

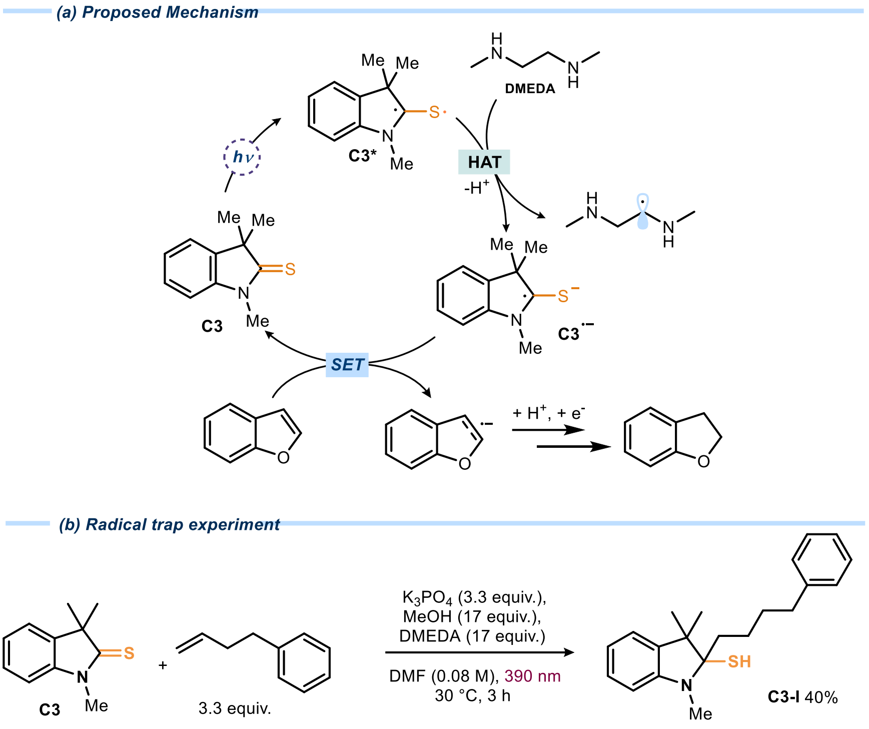

Figure S29. (a) Proposed mechanistic hypothesis for **C3** and (b) Evidence supporting the formation of a **C3** radical anion intermediate.

Overall, the mechanistic analysis demonstrates that **C3** operates through a distinct light-driven, HAT-initiated manifold that produces the radical anion **C3**$^{\bullet-}$ as the active photoreductant**.** This pathway accounts for the selective reactivity of **C3** towards heteroarenes and explains its divergence from **C2**.

Radical-trapping experiment for **C3**

To a 7 mL glass vial were added catalyst **C3** (46 mg, 0.24 mmol, 1 equiv.) and K_3PO_4 (170 mg, 0.8 mmol, 3.3 equiv.). The vial was sealed with a screw-cap septum, evacuated and backfilled with argon three times, and DMF was added via syringe.

Then MeOH (160 µL, 4.0 mmol, 17 equiv.), 3-butenylbenzene (120 µL, 4.0 mmol, 17 equiv.) and DMEDA (430 µL, 4.0 mmol, 17 equiv.) were added via syringe. The vial was placed in the photoreactor and stirred (600 rpm) at 30 ºC (reactor temperature) under 390 nm irradiation (2x PR160L-390 nm, 100% light intensity) for 3 hours. After the reaction was completed, the mixture was filtered and then the solvent was removed under reduced pressure (40 °C, < 15 mbar). The yield of **C3-I** (40 %) was determined *via* ^{1}H NMR analysis using CH_2Br_2 as the internal standard. The crude residue was purified by flash column chromatography using silica gel (hexane/AcOEt, 20:1) to afford **C3-I** as a yellow liquid in mixture with **C3**.

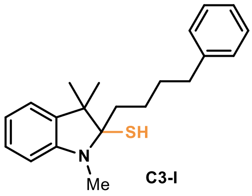

1,3,3-trimethyl-2-(4-phenylbutyl)indoline-2-thiol

^{1}H NMR (600 MHz, $CDCl_3$) δ 7.30 – 7.27 (m, 2H), 7.20 – 7.17 (m, 3H), 7.10 – 7.06 (m, 1H), 7.01 – 6.97 (m, 1H), 6.72 (td, J = 7.4, 1.0 Hz, 1H), 6.50 – 6.47 (m, 1H), 2.71 (s, 3H), 2.66 (t, J = 7.4 Hz, 2H), 1.72 – 1.67 (m, 2H), 1.53 – 1.45 (m, 2H), 1.36 – 1.26 (m, 5H), 1.06 (s, 3H).

^{13}C NMR (151 MHz, $CDCl_3$) δ 151.7, 142.5, 139.5, 128.4, 128.3, 127.3, 125.7, 121.4, 118.4, 107.8, 43.0, 35.8, 32.2, 28.6, 27.3, 23.3.

HRMS (TOF MS LD+) $C_{21}H_{27}NS$ $[M+1]^+$: found 326.1942, required 326.1864.

F.8.1 Evaluation of other Potential Pathways

Because sulfur extrusion is involved in the formation of some degradation products of both catalysts (**A** and **B** for **C2**, **D**, **E** and **F** for **C3**, see Figure S11 & S13), we considered whether polysulfide anions might form in situ. Chiba and co-workers[15] have shown these species to act as competent photoreductants. We therefore replaced **C2** and **C3** with K_2S_x under otherwise identical conditions (Figure 30). No reduction of anisole was observed, whereas benzofuran was reduced in 15% yield, much lower than the yield obtained with **C3**. This indicates that polysulfides cannot account for the behaviour of **C2** and may contribute only marginally to the reactivity observed with **C3**.

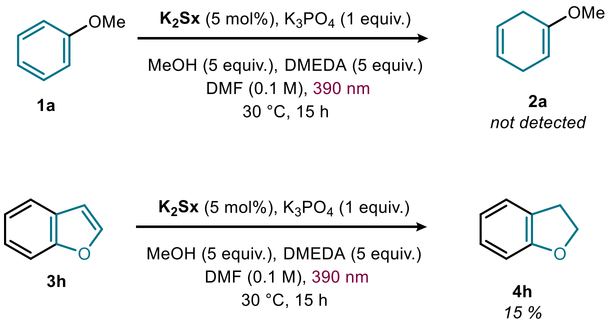

Figure 30. Evaluation of polysulfide anions (K_2S_x) as alternative photoreductants.

G.9. Deuterium Labelling Experiments

The reduction of **1g** was carried out using the general procedure **B** with CD_3OD (instead of MeOH) and DIPEA (instead of DMEDA, Figure S31). After the reaction was finished, the solvent was removed under reduced pressure, the crude product was filtered through silica and washed with EtOAc. The ^1H NMR spectrum was then measured in $CDCl_3$. The deuterium incorporation (red and purple dot) was determined by using the alkene peak (blue dot) as reference.

When using DMEDA, no deuterium incorporation was detected in product **2g** under the standard conditions, presumably because fast proton/deuterium exchange between the free N–H and CD_3OD.

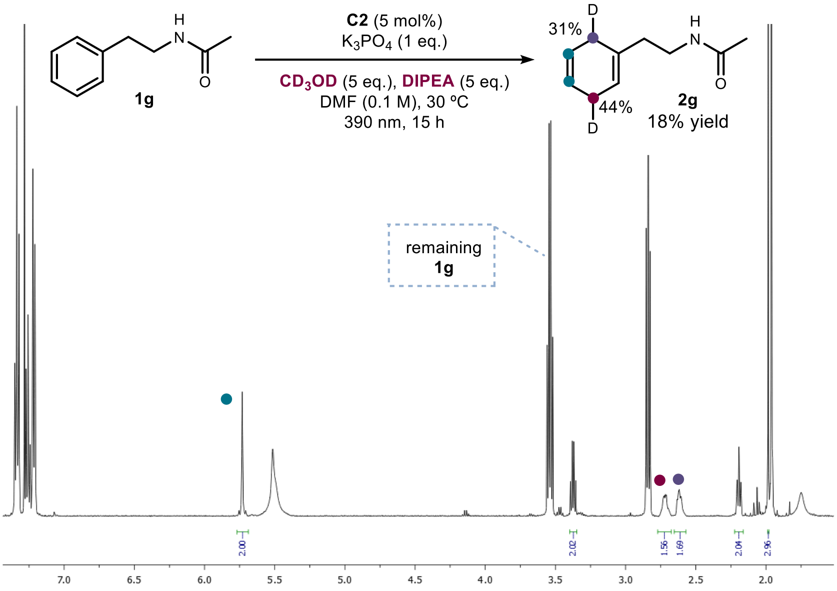

Figure S31. ^1H NMR in $CDCl_3$ of the 1,4-dihydro product **2g** using CD_3OD as deuterium source.

G. References

[1] Wu, S.; Schiel, F.; Melchiorre, P. A General Light-Driven Organocatalytic Platform for the Activation of Inert Substrates. *Angew. Chem. Int. Ed.* **2023**, *62*, e202306364.

[2] Wang, C.; Liu; L. "NHC-catalyzed oxindole synthesis via single electron transfer" *Org. Chem. Front.* **2021**, *8*, 1454–1460.

[3] Schiel, F.; Peinsipp, C.; Kornigg, S.; Böse, D. A 3D-Printed Open Access Photoreactor Designed for Versatile Applications in Photoredox- and Photoelectrochemical Synthesis. *ChemPhotoChem* **2021**, *5*, 431−437.

[4] Williams, C. M.; Mander, L. N. Chromatography with silver nitrate. *Tetrahedron* **2001**, *57*, 425–447.

[5] O'Byrne, A.; Murray, C.; Keegan, D.; Palacio, C.; Evans, P.; Morgan, B. S. The Thio-Adduct Facilitated, Enzymatic Kinetic Resolution of 4-hydroxycyclopentenone and 4-hydroxycyclohexenone. *Org. Biomol. Chem.* **2010**, *8*, 539–545.

[6] Abraham, R. J.; Canton, M.; Griffiths, L. Proton chemical shifts in NMR: Part 17. Chemical shifts in alkenes and anisotropic and steric effects of the double bond. *Magn. Reson. Chem.* **2001**, *39*, 421–431.

[7] O'Mahony, M. J.; More O'Ferrall, R. A.; Boyd, D. R.; Lam, C. M.; O'Donoghue, A. C. "Substituent effects on the dehydration of arene hydrates in aqueous solution" *J. Phys. Org. Chem.* **2013**, *26*, 989–996.

[8] Gao, Y.; Kubota, K.; Ito, H. Mechanochemical Approach for Air-Tolerant and Extremely Fast Lithium-Based Birch Reductions in Minutes. *Angew. Chem. Int. Ed.* **2023**, *62*, e202217723.

[9] Kondo, K.; Kubota, K.; Ito, H. Mechanochemistry enabling highly efficient Birch reduction using sodium lumps and *D*-(+)-glucose. *Chem. Sci.* **2024**, *15*, 4452–4457.

[10] Burrows, J.; Kamo, S.; Koide, K. Scalable Birch reduction with lithium and ethylenediamine in tetrahydrofuran. *Science* **2021**, *374*, 741–746.

[11] Peters, B. K.; Rodriguez, K. X.; Reisberg, S. H.; Beil, S. B.; Hickey, D. P.; Kawamata, Y.; Collins, M.; Starr, J.; Chen, L.; Udyavara, S.; Klunder, K.; Gorey, T. J.; Anderson, S. L.; Neurock, M.; Minteer, S. D.; Baran, P. S. Scalable and Safe Synthetic Organic Electroreduction Inspired by Li-Ion Battery Chemistry. *Science* **2019**, *363*, 838−845.

[12] McElroy, T.; Thomas, J. B.; Brine, G. A.; Navarro, H. A.; Deschamps, J.; Carroll F. I. A Practical Synthesis of the Kappa Opioid Receptor Selective Agonist (+)-5R,7S,8S-N-Methyl-N-[7-(1-pyrrolidinyl)-1-oxospiro[4,5]dec-8-yl]benzeneacetamide (U69,593). *Synlett* **2008**, *6*, 943–947.

[13] Pavlishchuk V. V.; Addison A. W. *Inorganica Chim. Acta* **2000**, *298*, 97–102.

[14] Buzzetti, L.; Crisenza, G. E. M.; Melchiorre, P. Mechanistic Studies in Photocatalysis. *Angew. Chem. Int. Ed*. **2019**, *58*, 3730–3747.

[15] Li, H.; Tang, X.; Pang J. H.; Wu, X.; Yeow, E. K. L.; Wu J., Chiba, S. Polysulfide Anions as Visible Light Photoredox Catalysts for Aryl Cross-Couplings. *J. Am. Chem. Soc.* **2021**, 143, 1, 481–487.

H. NMR Spectra

^{1}H NMR (500 MHz, $CDCl_3$)

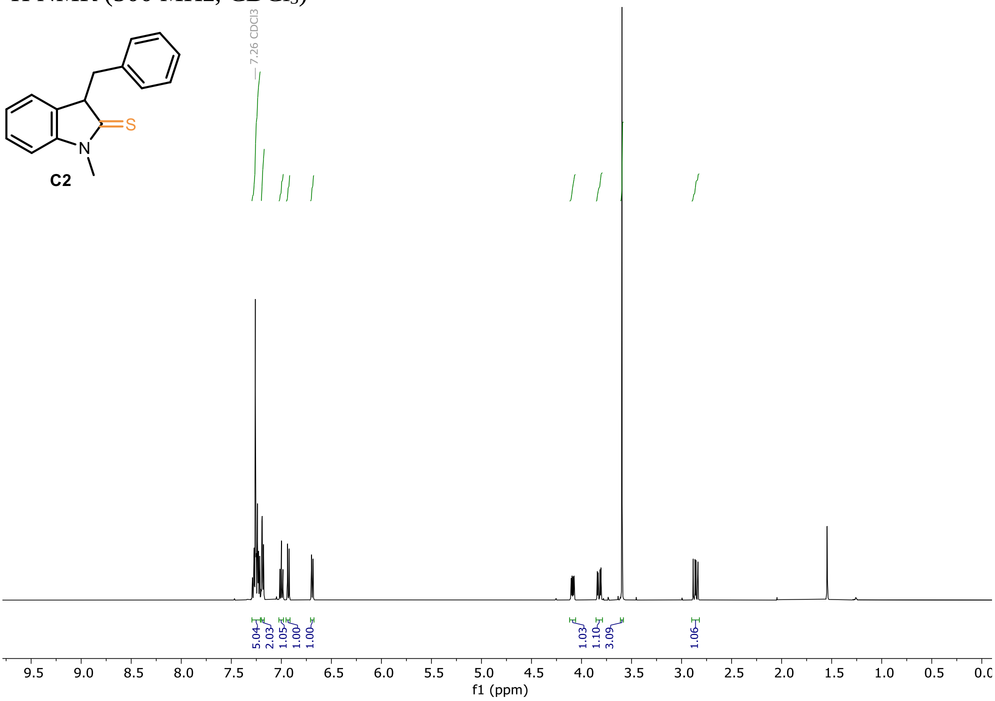

C2

^{13}C NMR (126 MHz, $CDCl_3$)

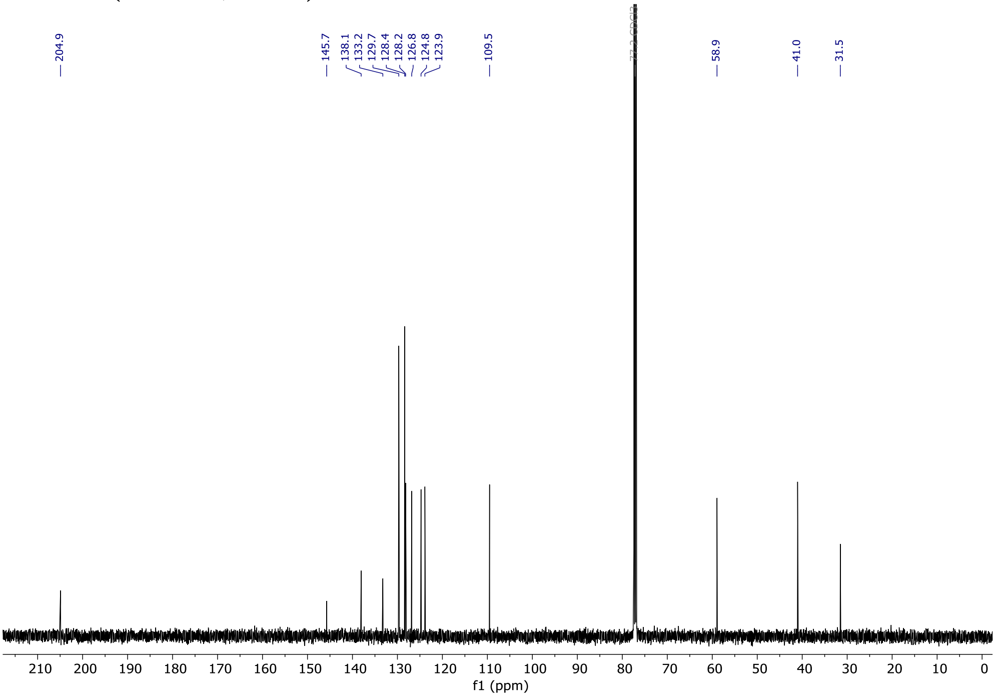

^{1}H NMR in DMF-d_7

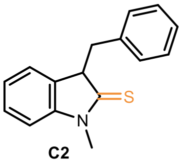

C2

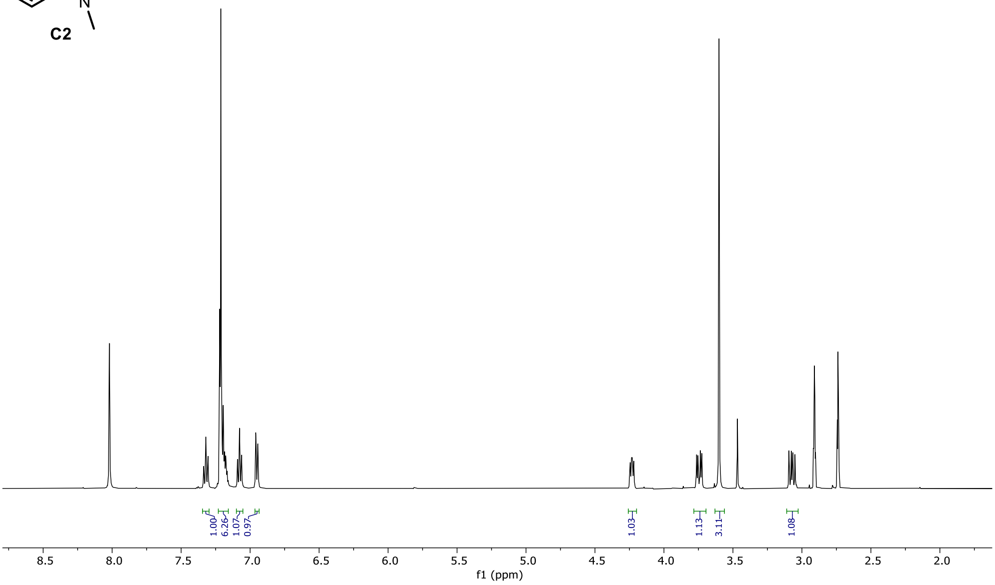

^{13}C NMR in DMF-d_7

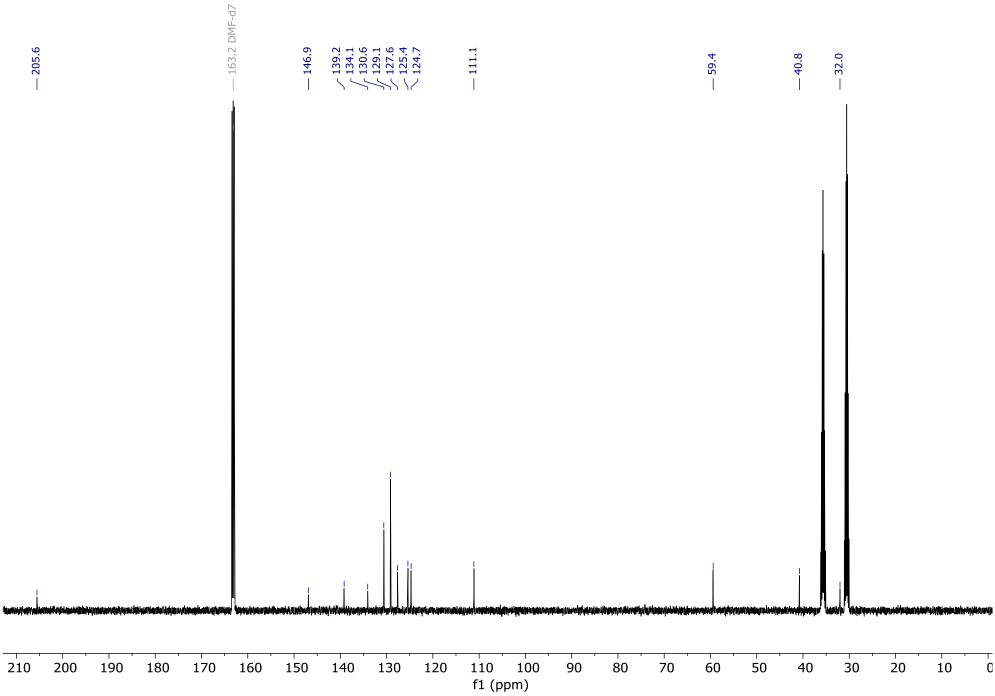

^{1}H NMR in DMF-d_7

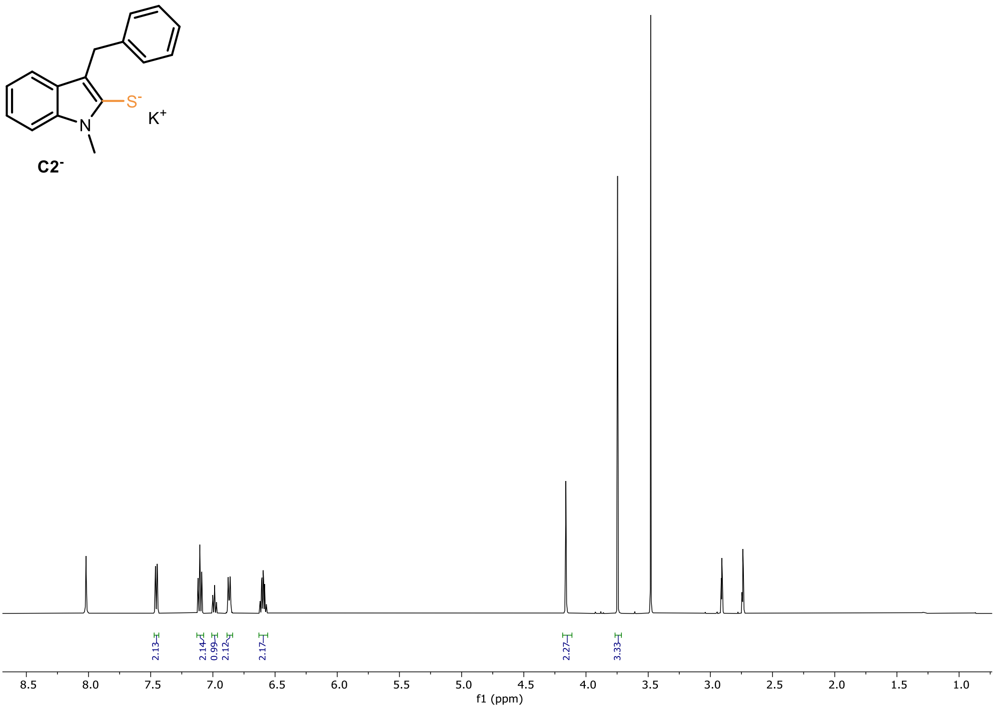

^{13}C NMR in DMF-d_7

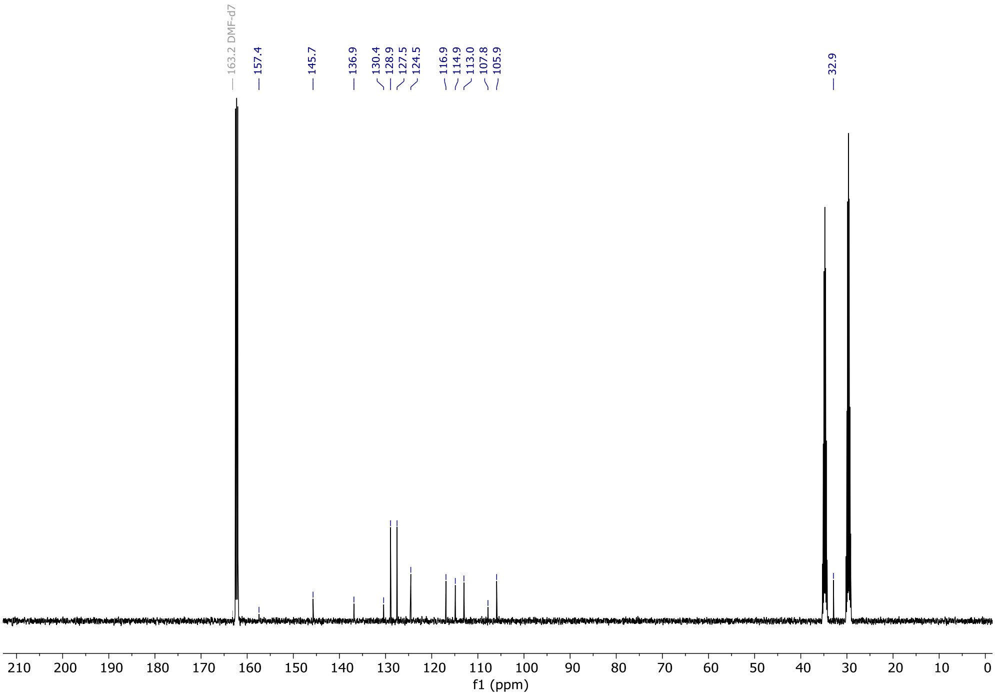

^{1}H NMR (500 MHz, $CDCl_3$)

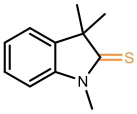

C3

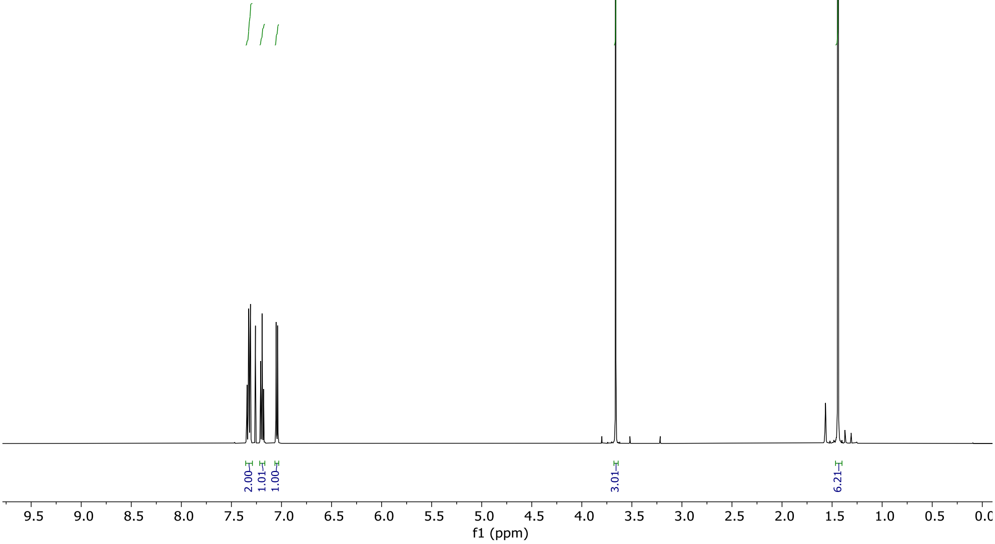

^{13}C NMR (101 MHz, $CDCl_3$)

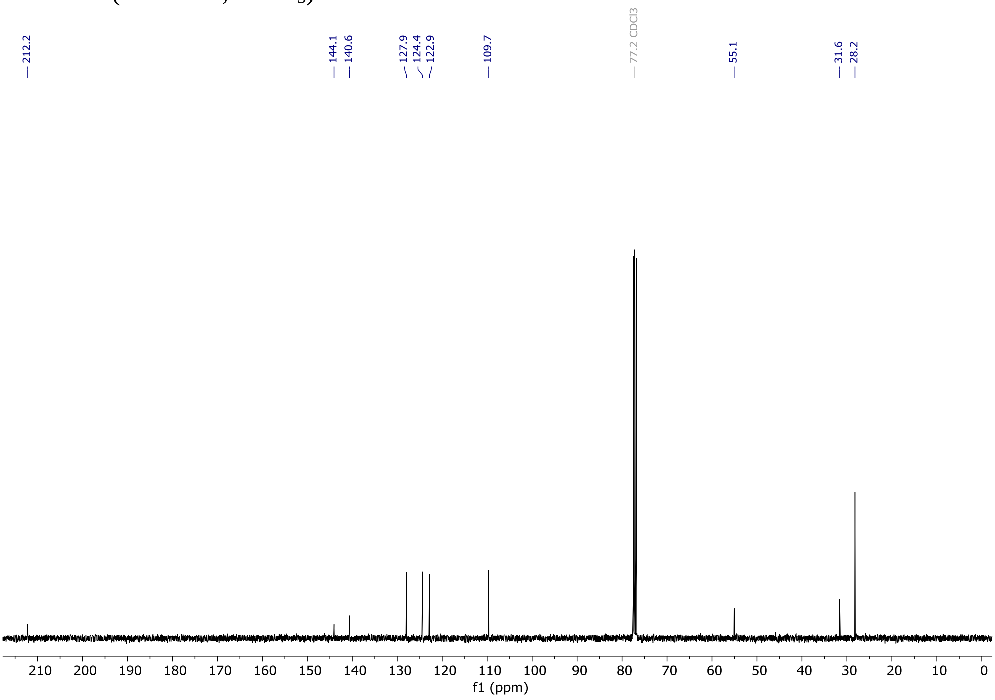

^{1}H NMR (500 MHz, $CDCl_3$)

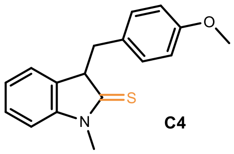

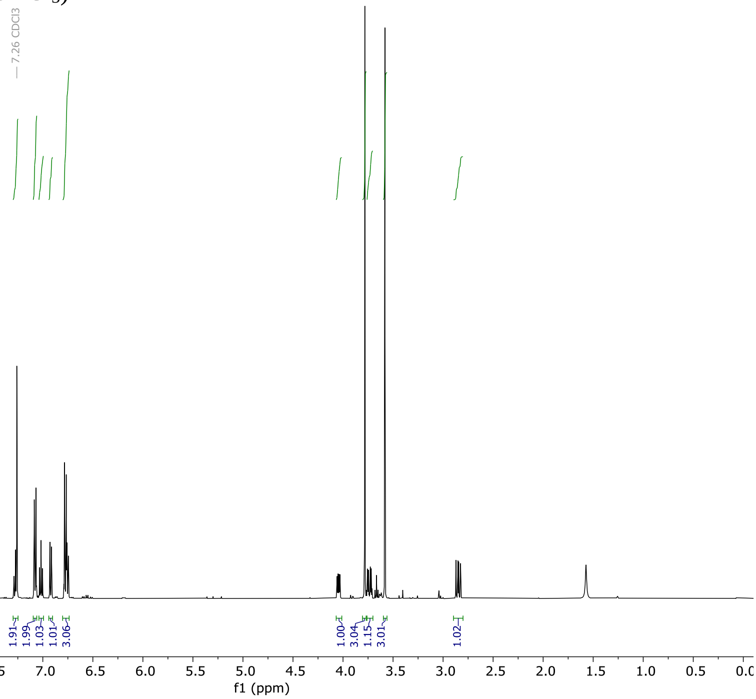

^{13}C NMR (126 MHz, $CDCl_3$)

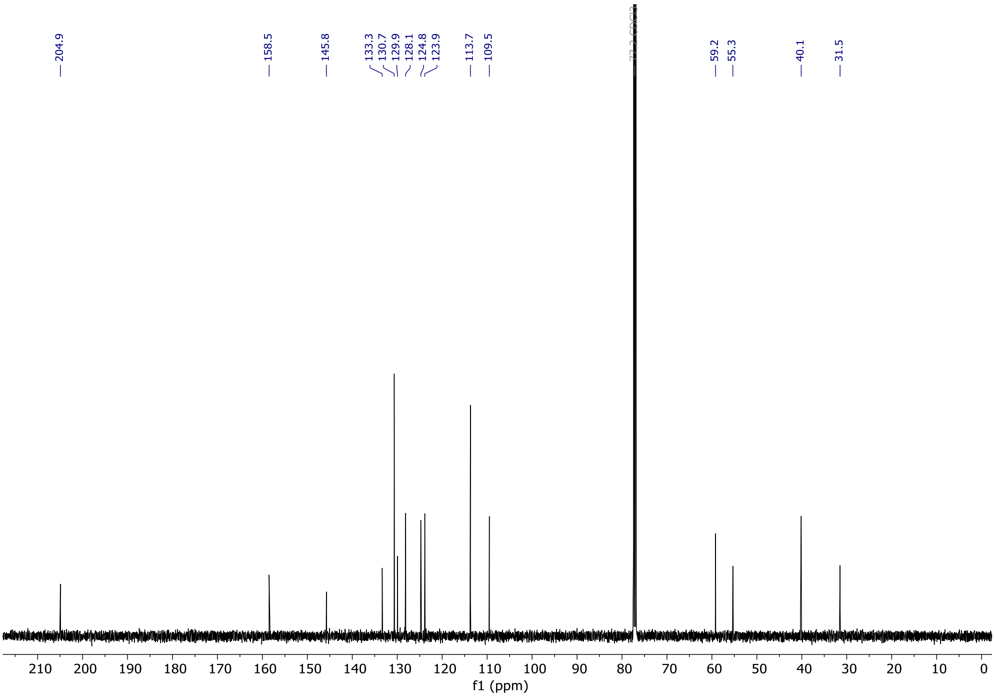

^{1}H NMR (500 MHz, $CDCl_3$)

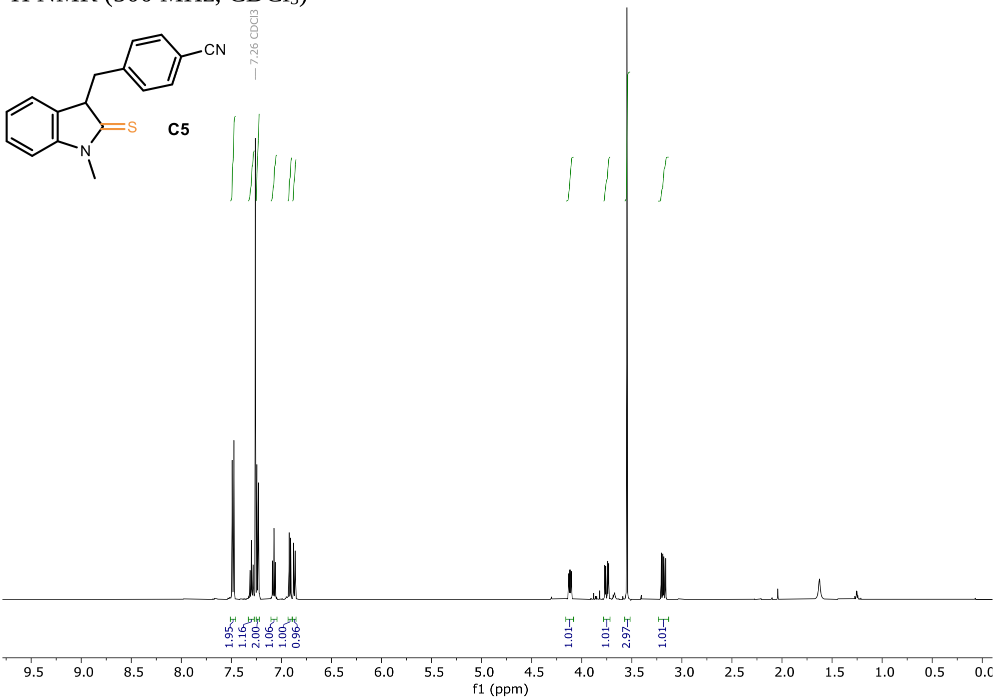

^{13}C NMR (126 MHz, $CDCl_3$)

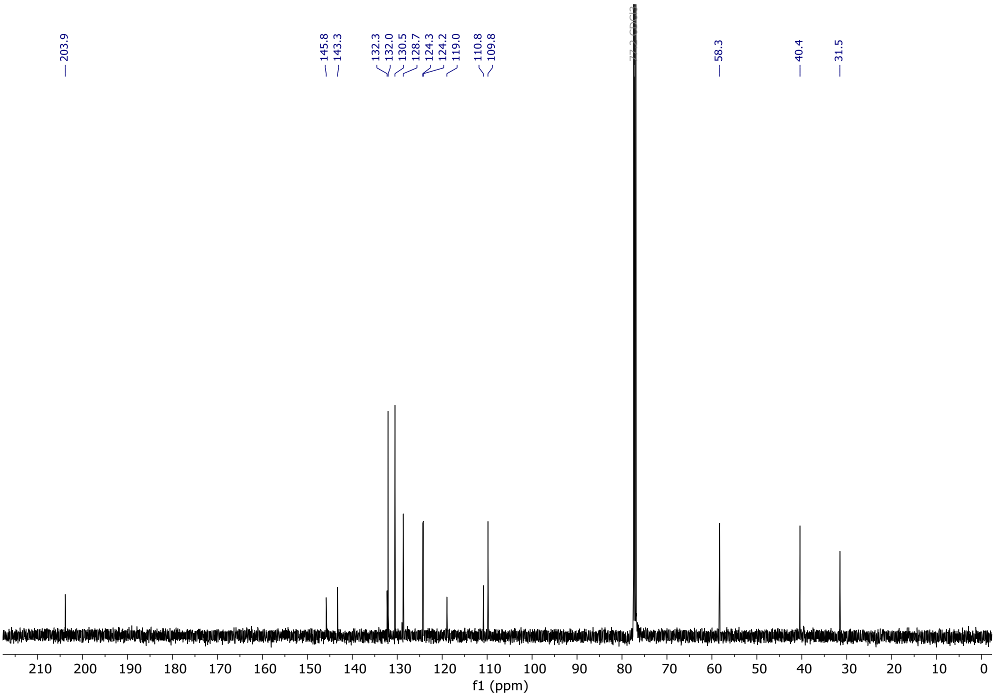

^{1}H NMR (500 MHz, $CDCl_3$)

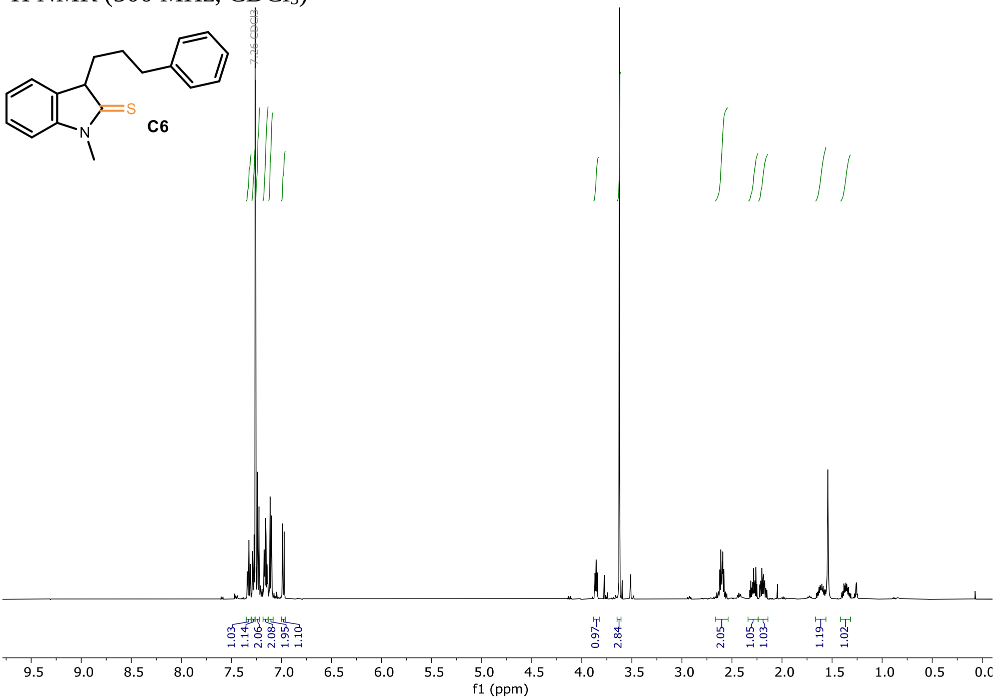

^{13}C NMR (126 MHz, $CDCl_3$)

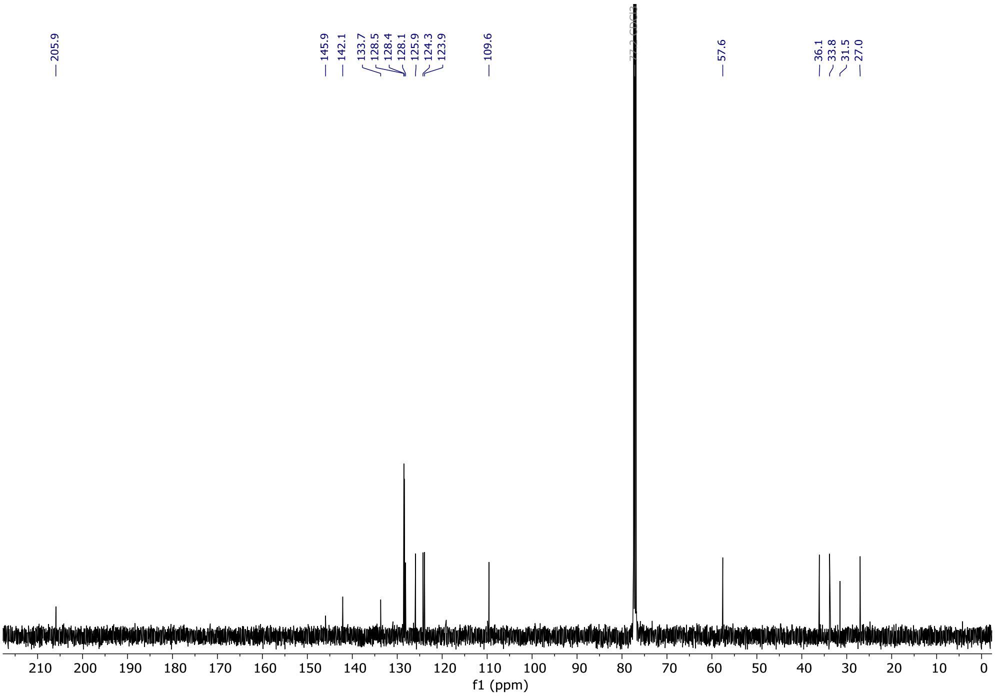

^{1}H NMR (500 MHz, $CDCl_3$)

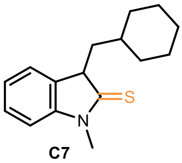

C7

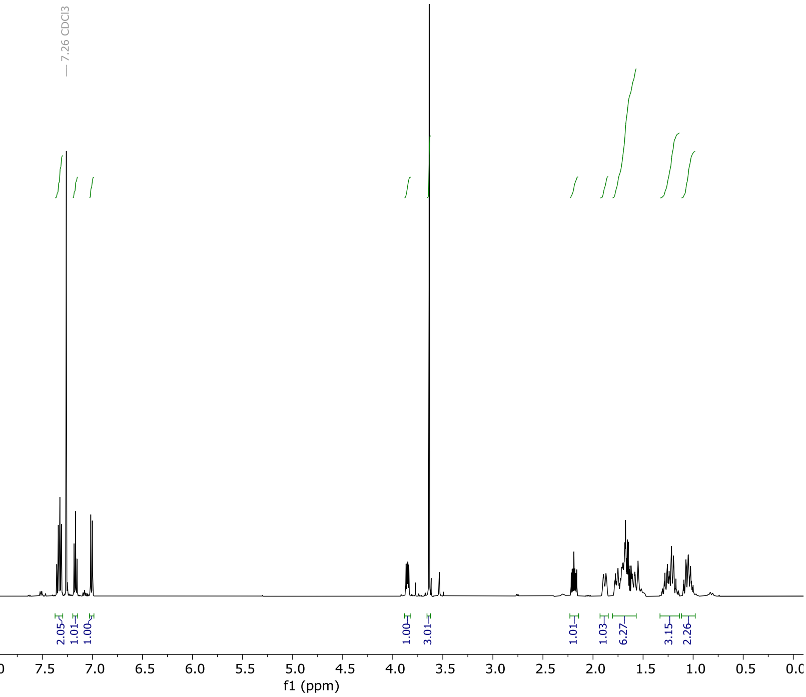

^{13}C NMR (126 MHz, $CDCl_3$)

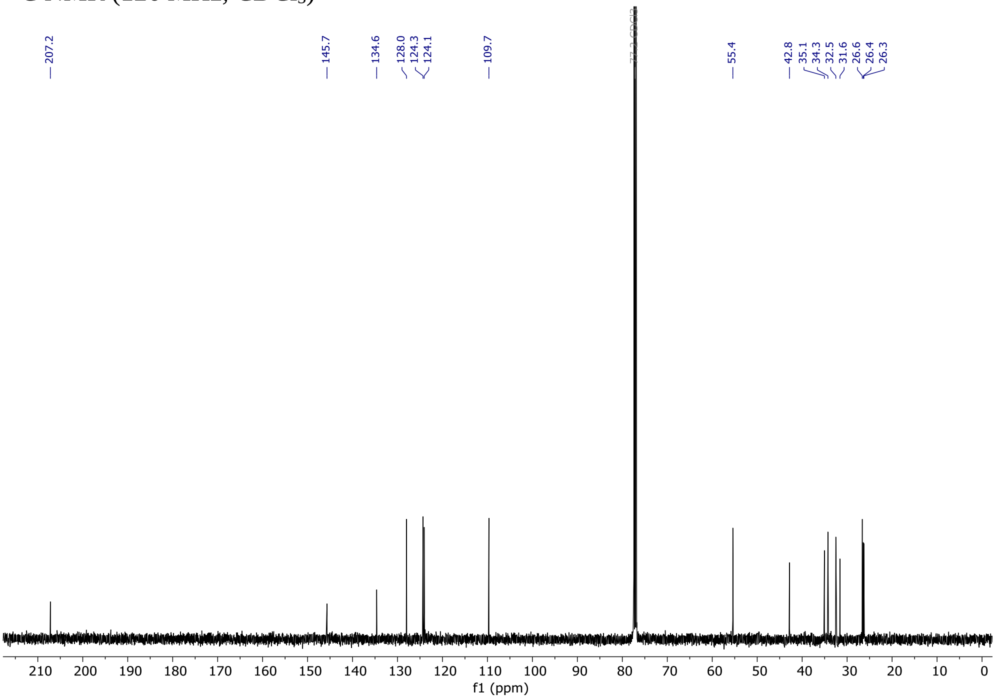

^{1}H NMR (500 MHz, $CDCl_3$)

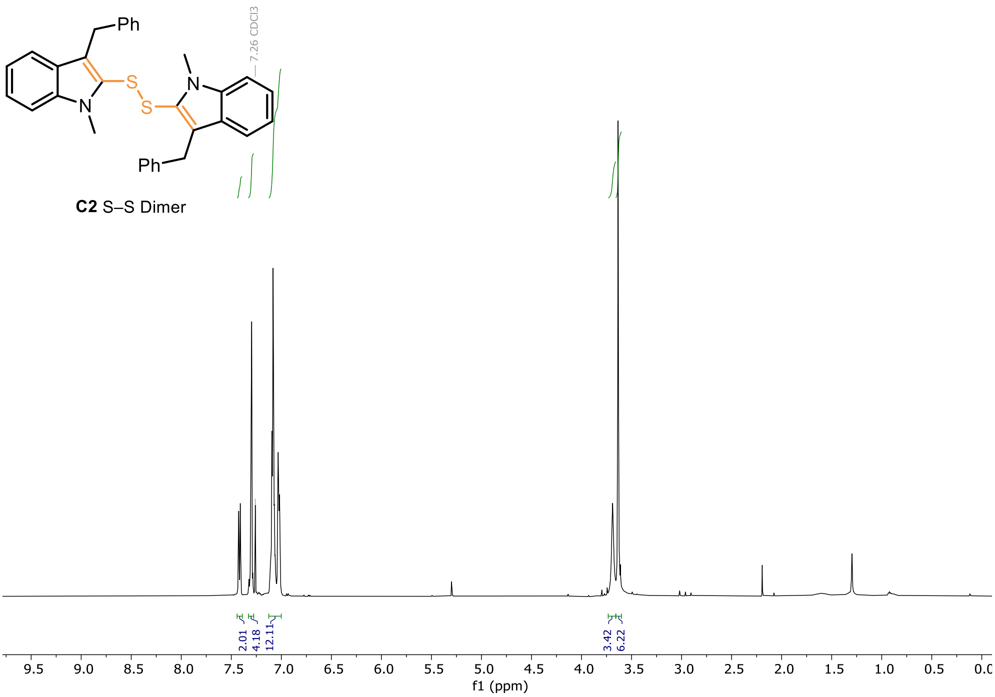

^{13}C NMR (126 MHz, $CDCl_3$)

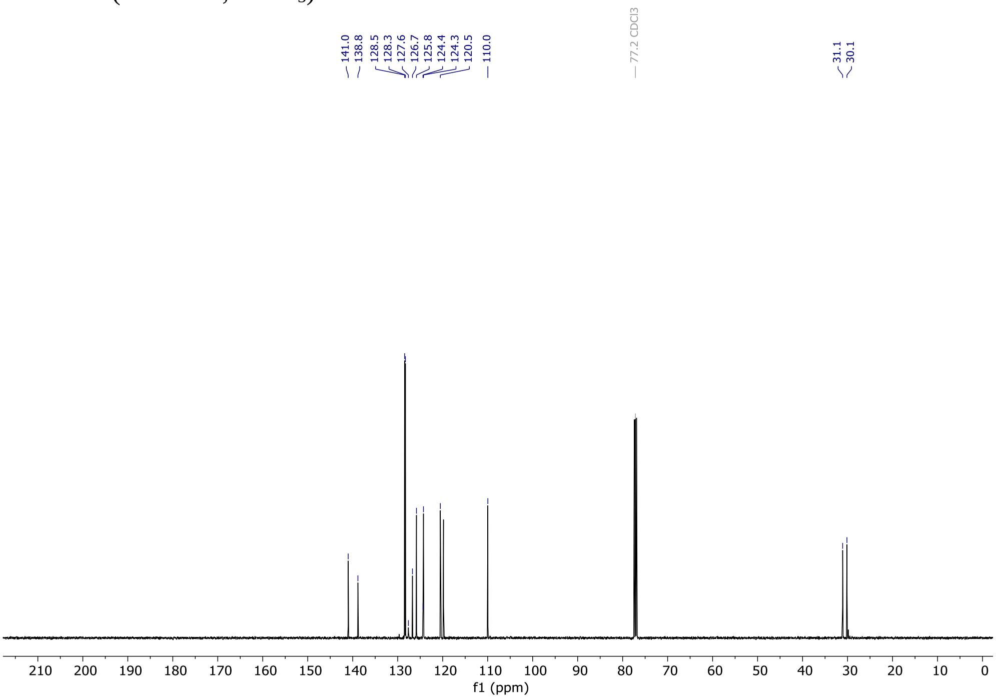

^{1}H NMR (500 MHz, $CDCl_3$)

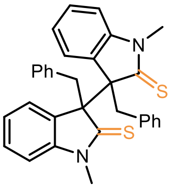

C2 C–C Dimer

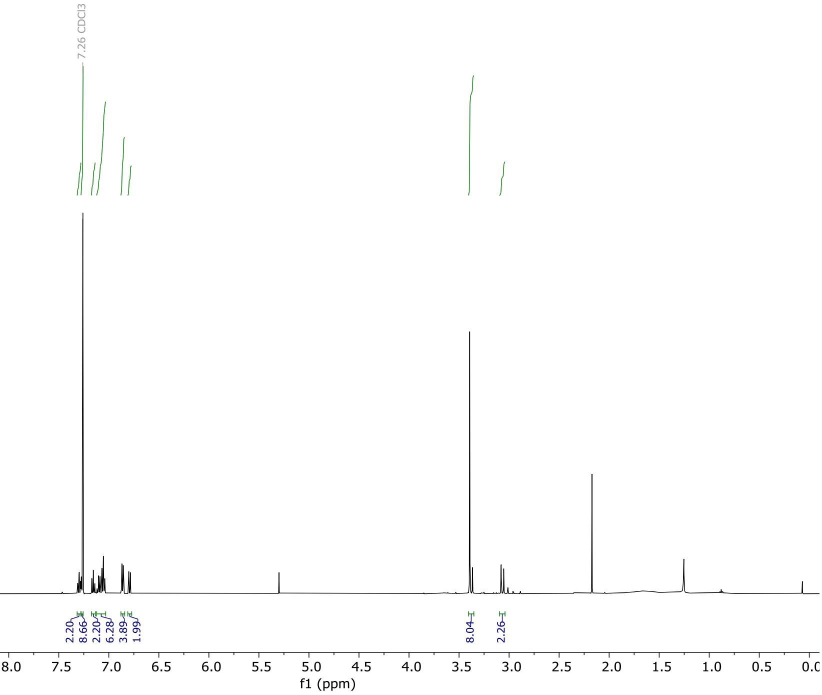

^{13}C NMR (126 MHz, $CDCl_3$)

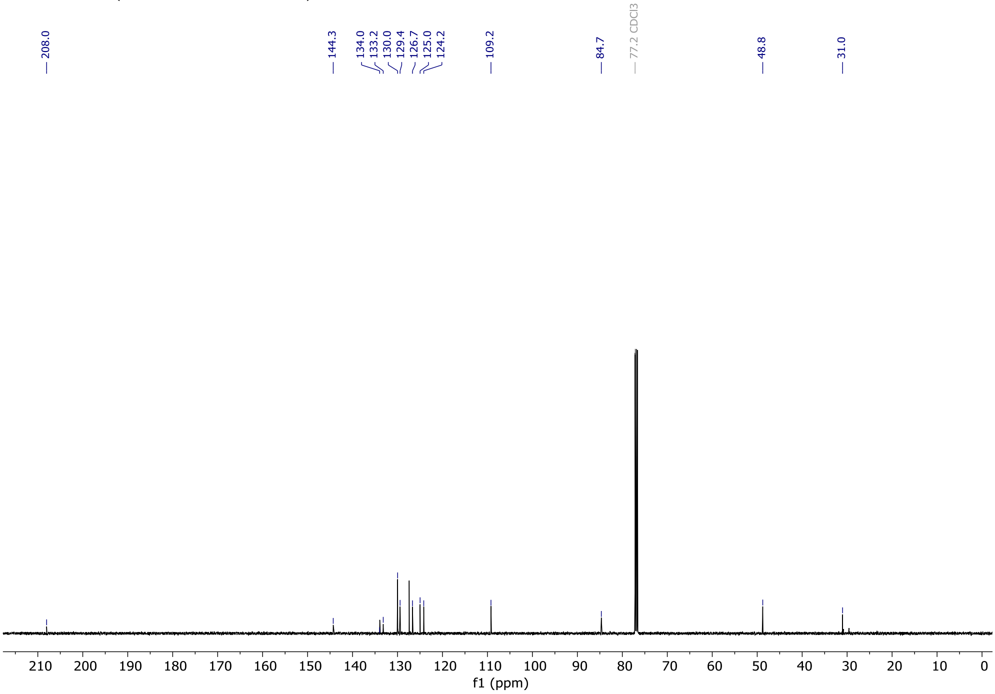

^{1}H NMR (500 MHz, $CDCl_3$)

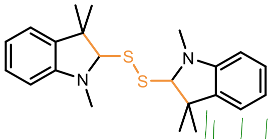

C3 S–S Dimer

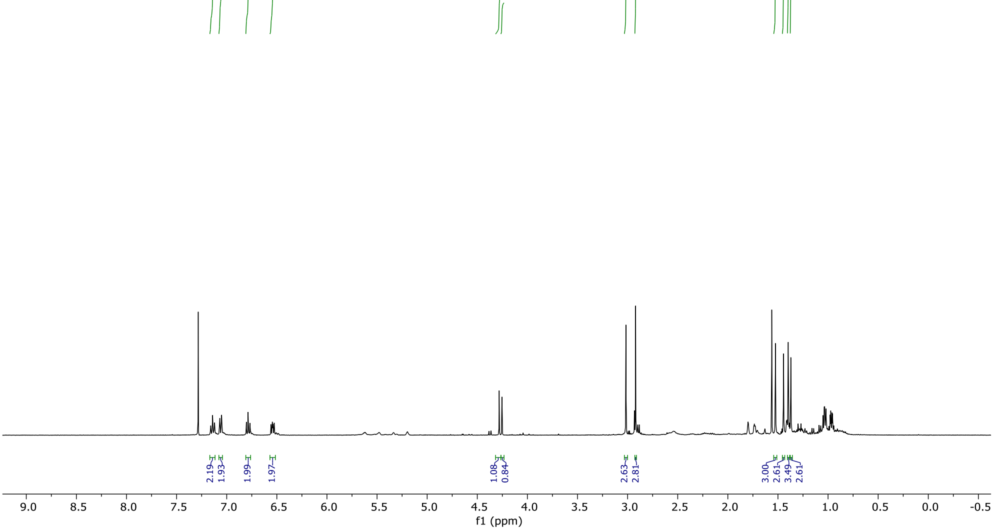

^{13}C NMR (126 MHz, $CDCl_3$)

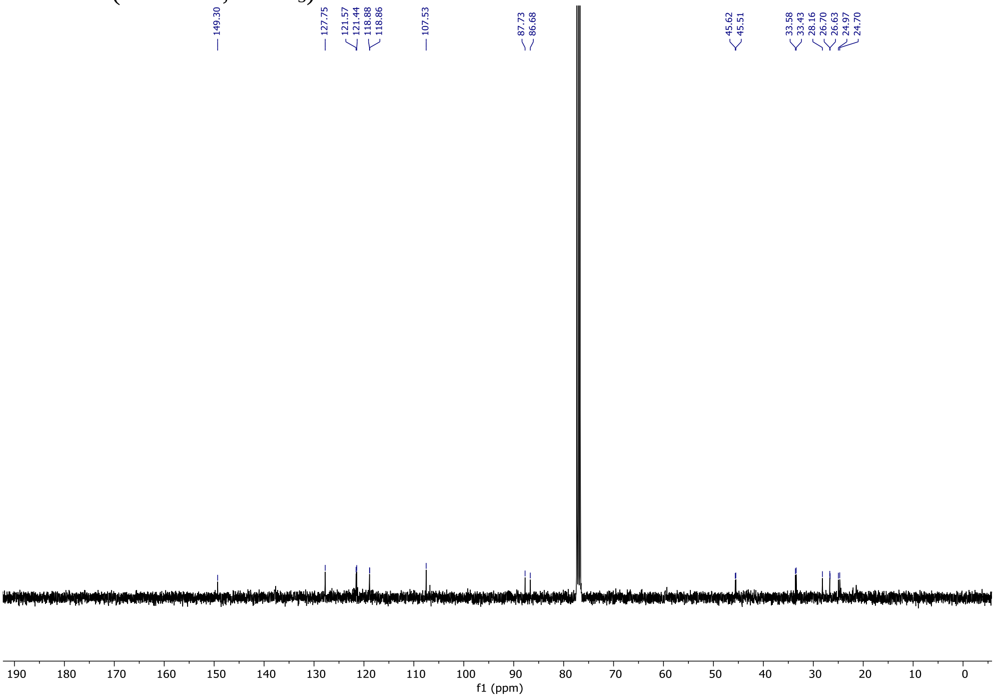

^{1}H NMR of the crude mixture in $CDCl_3$

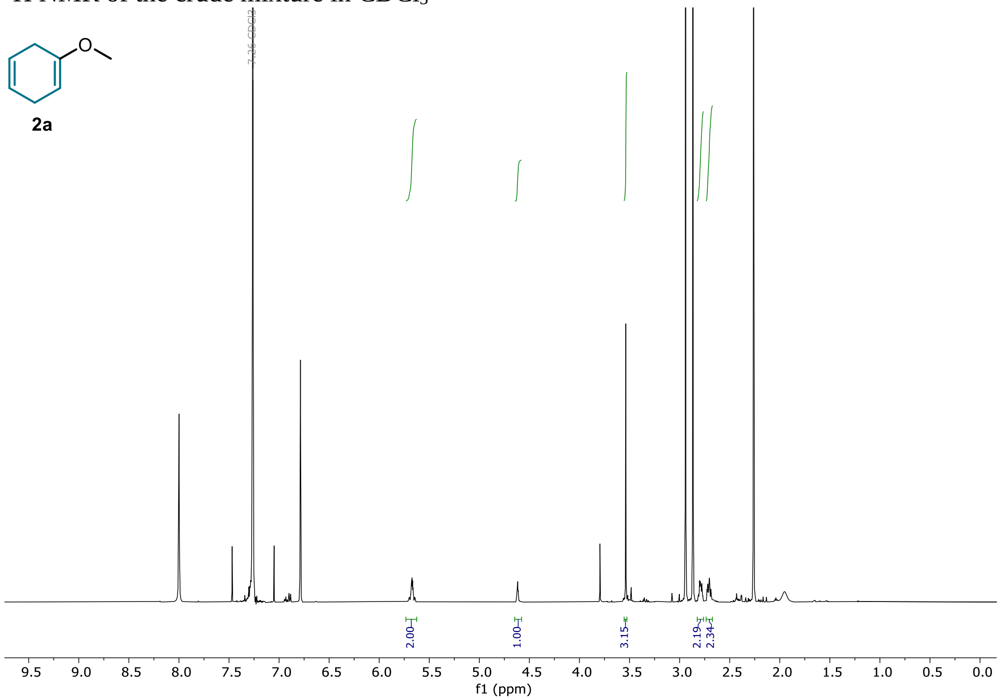

^{1}H NMR of the crude mixture in $CDCl_3$

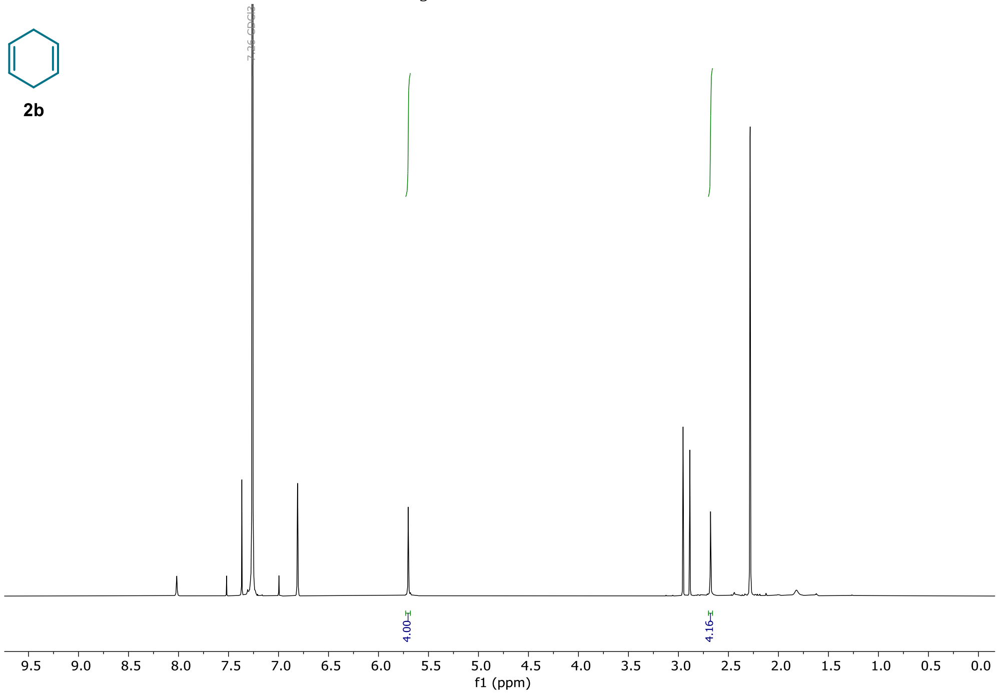

^{1}H NMR of the crude mixture in $CDCl_3$

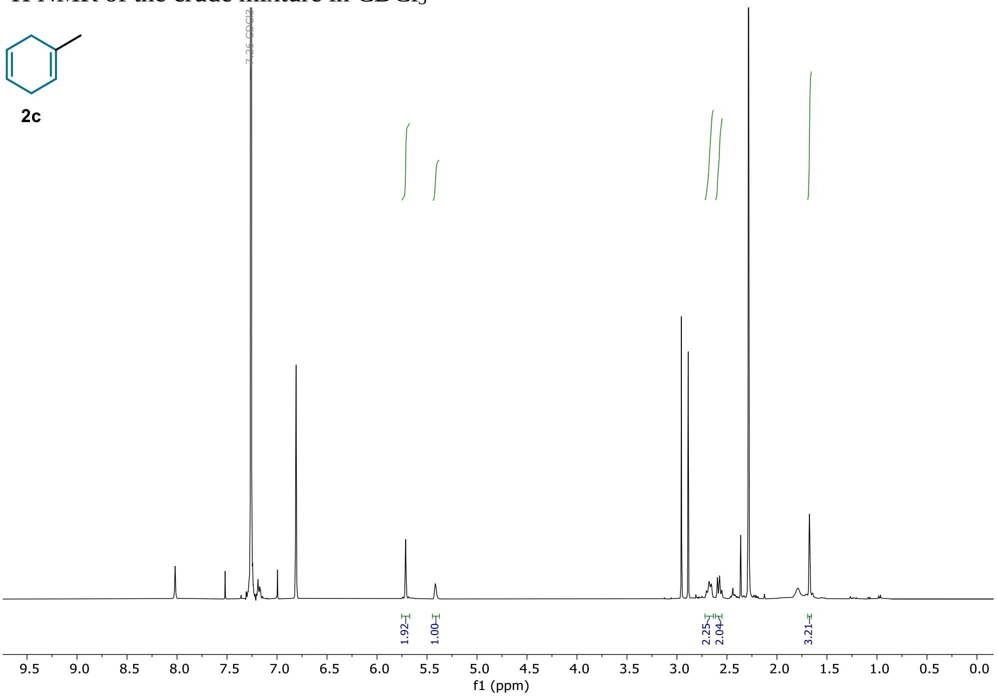

^{1}H NMR of the crude mixture in $CDCl_3$

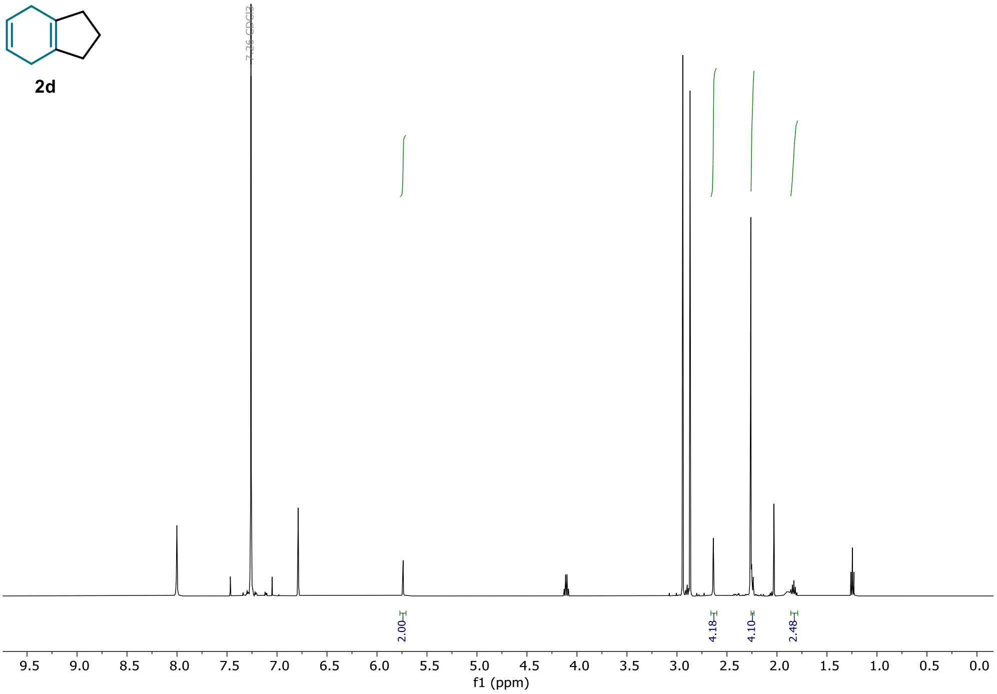

^{1}H NMR (600 MHz, $CDCl_3$)

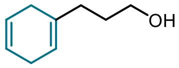

2e

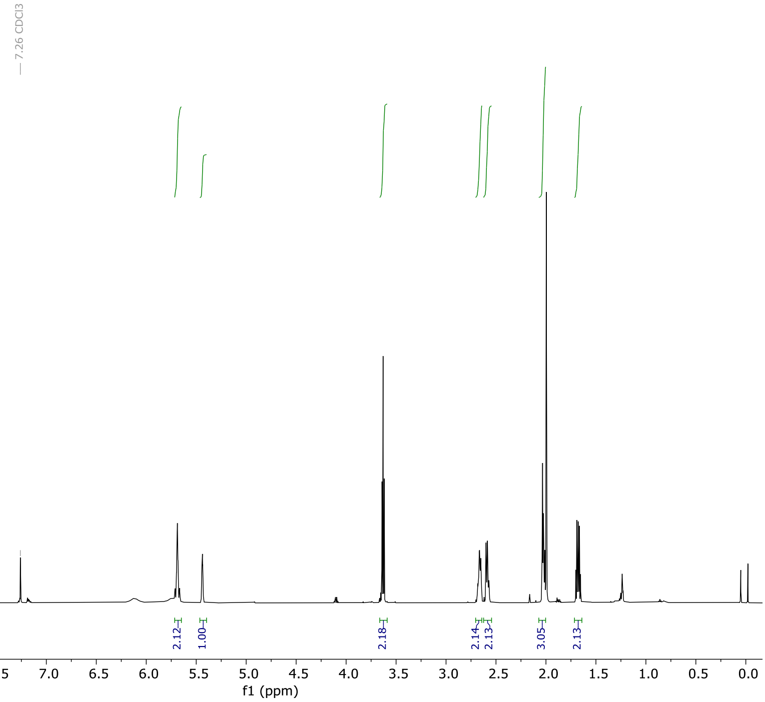

^{13}C NMR (151 MHz, $CDCl_3$)

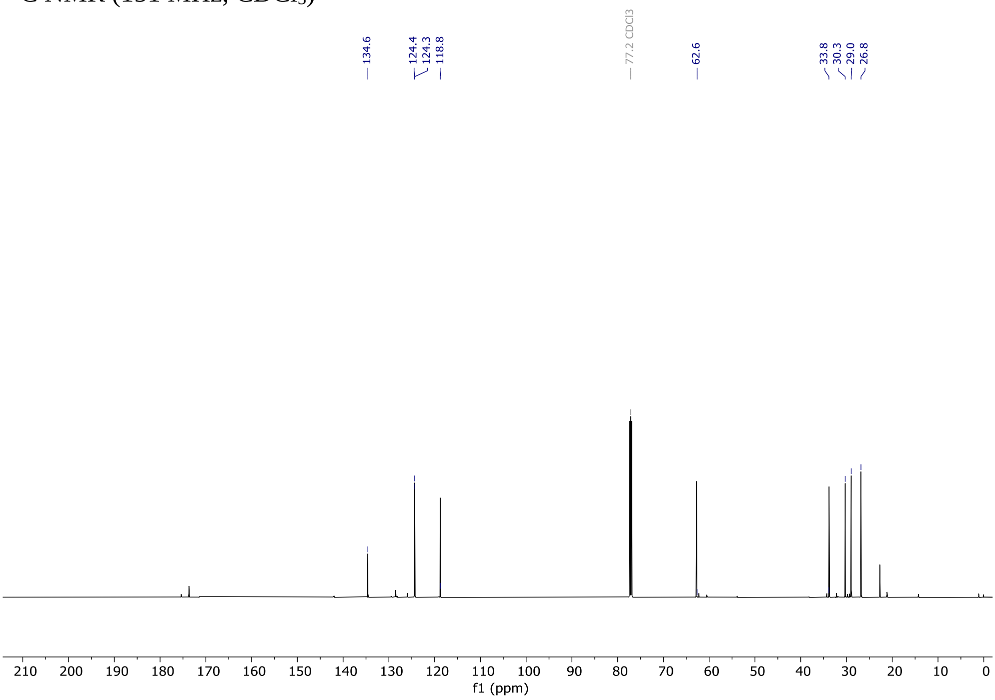

^{1}H NMR (600 MHz, $CDCl_3$)

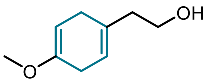

2f

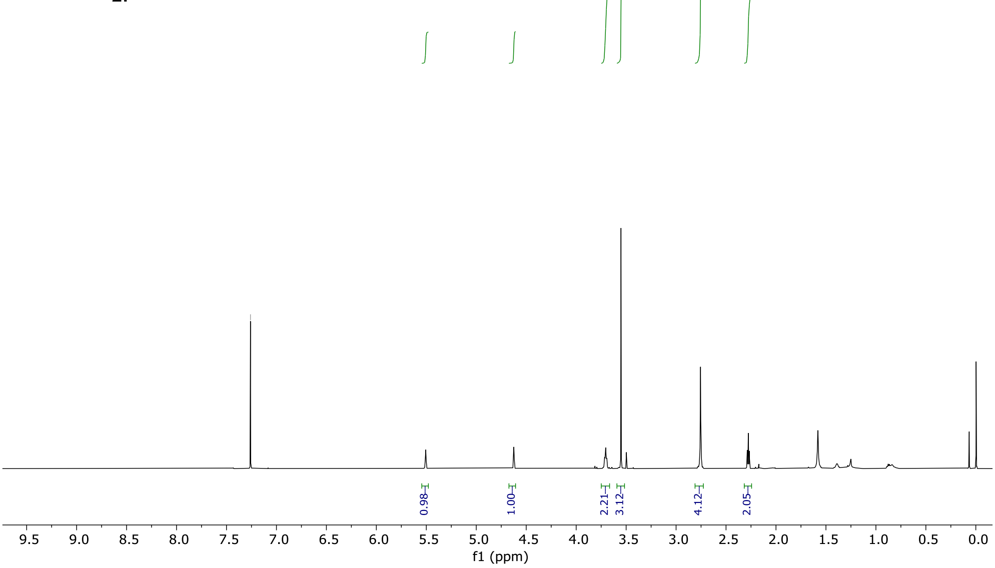

^{13}C NMR (151 MHz, $CDCl_3$)

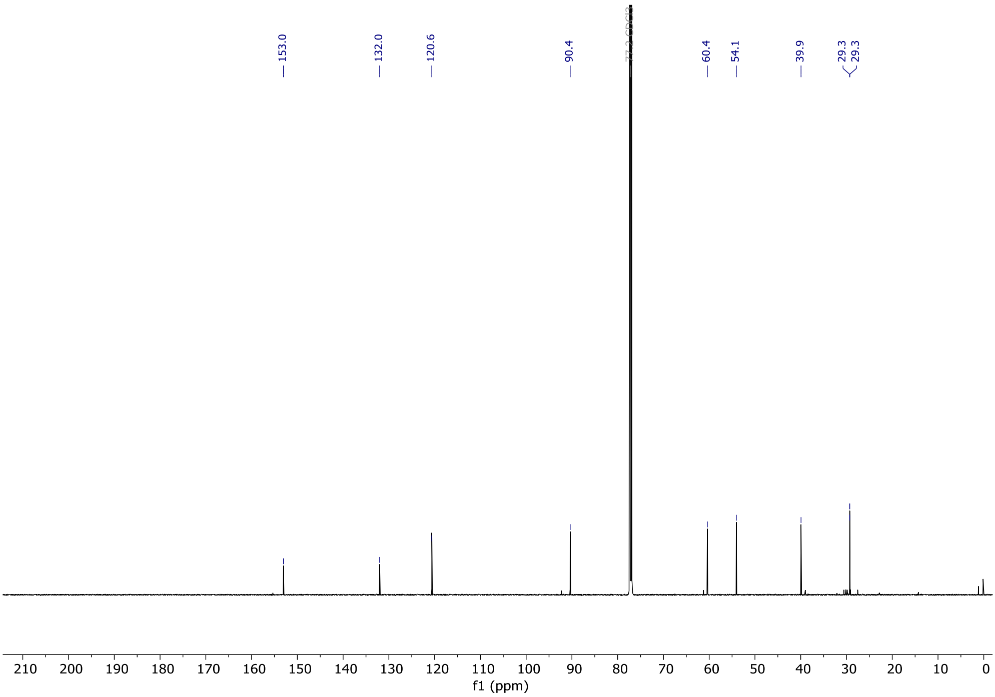

^{1}H NMR (500 MHz, $CDCl_3$)

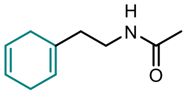

2g

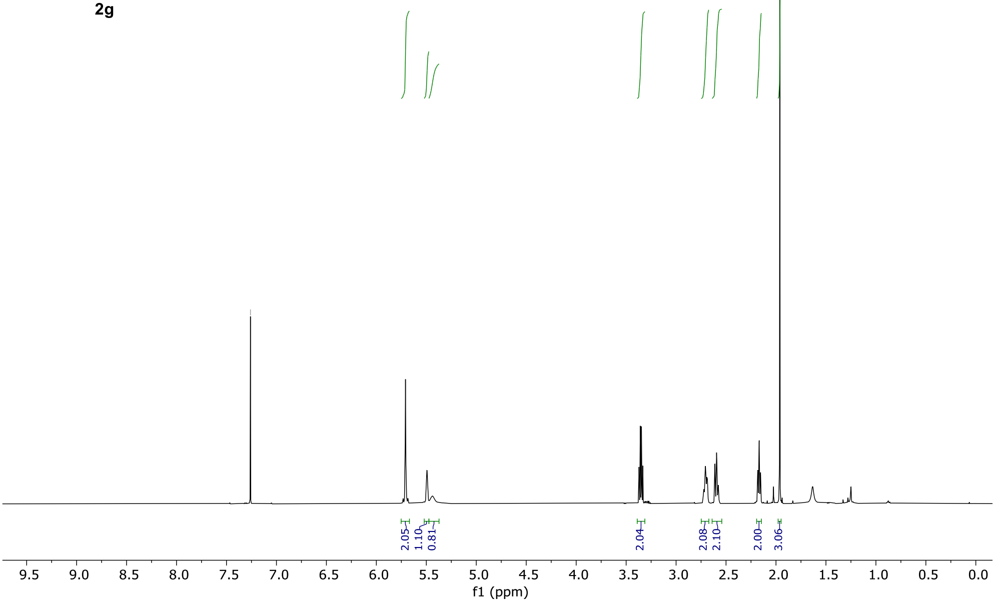

^{13}C NMR (126 MHz, $CDCl_3$)

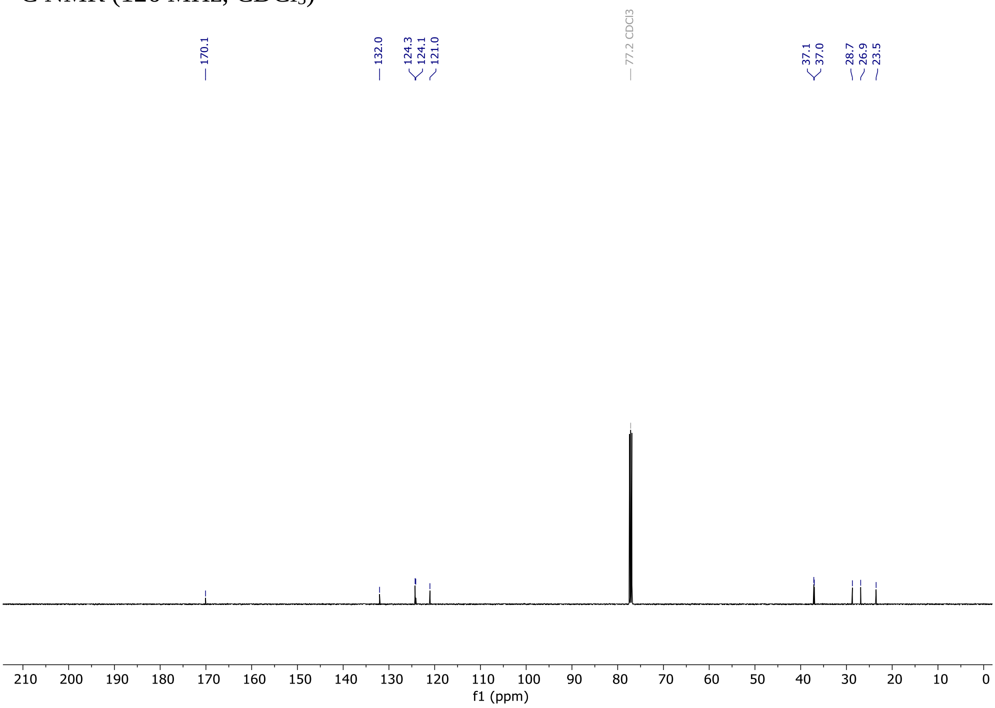

^{1}H NMR (600 MHz, $CDCl_3$)

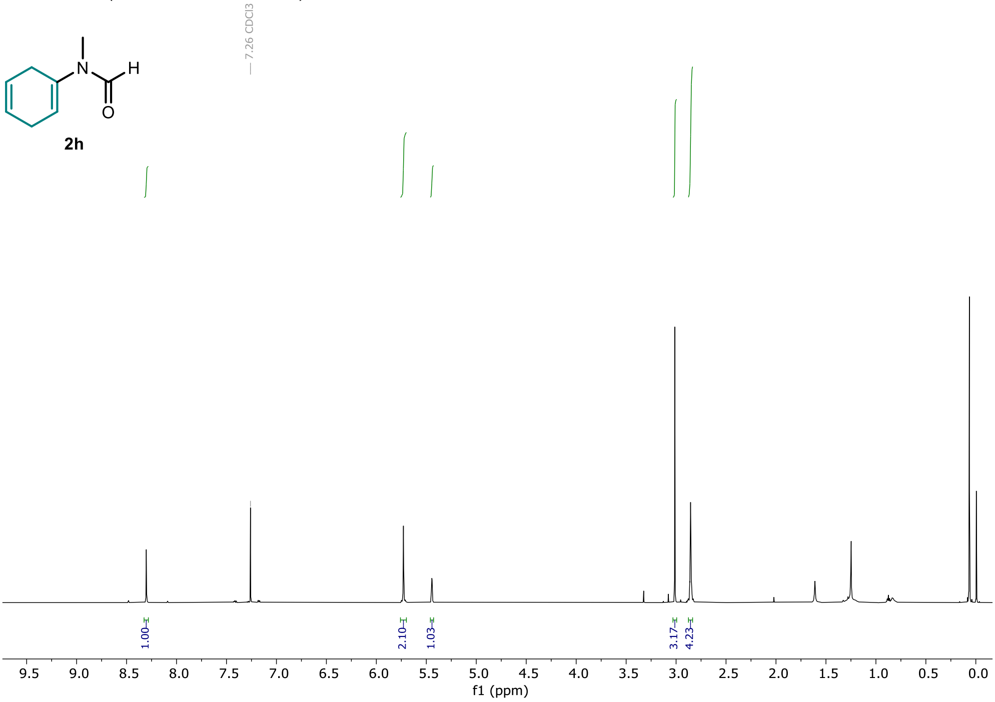

2h

^{1}H NMR (151 MHz, $CDCl_3$)

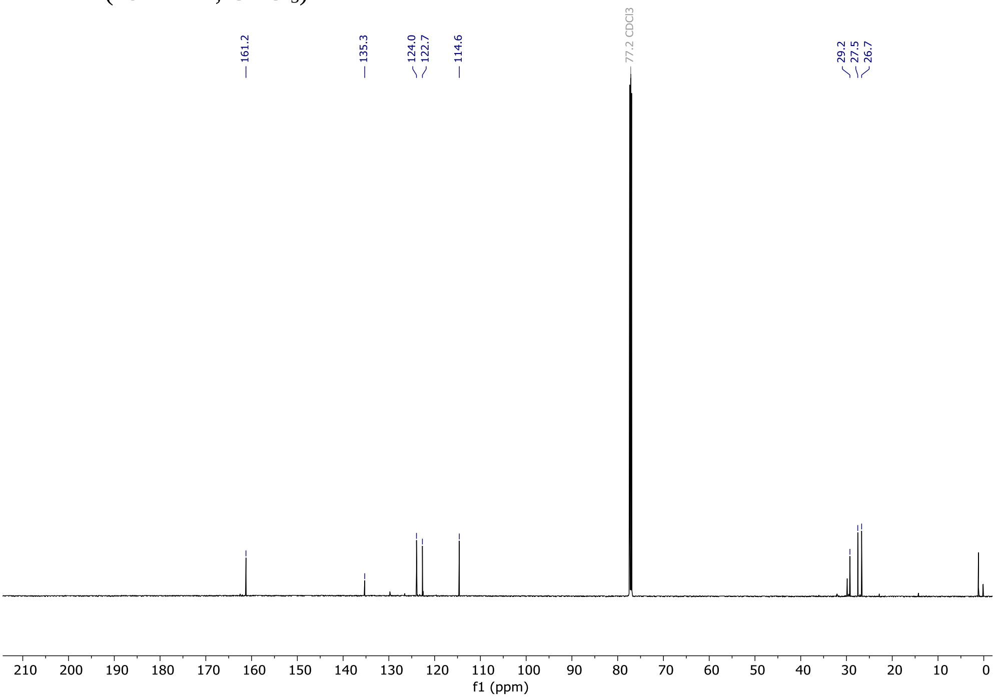

^{1}H NMR (600 MHz, $CDCl_3$)

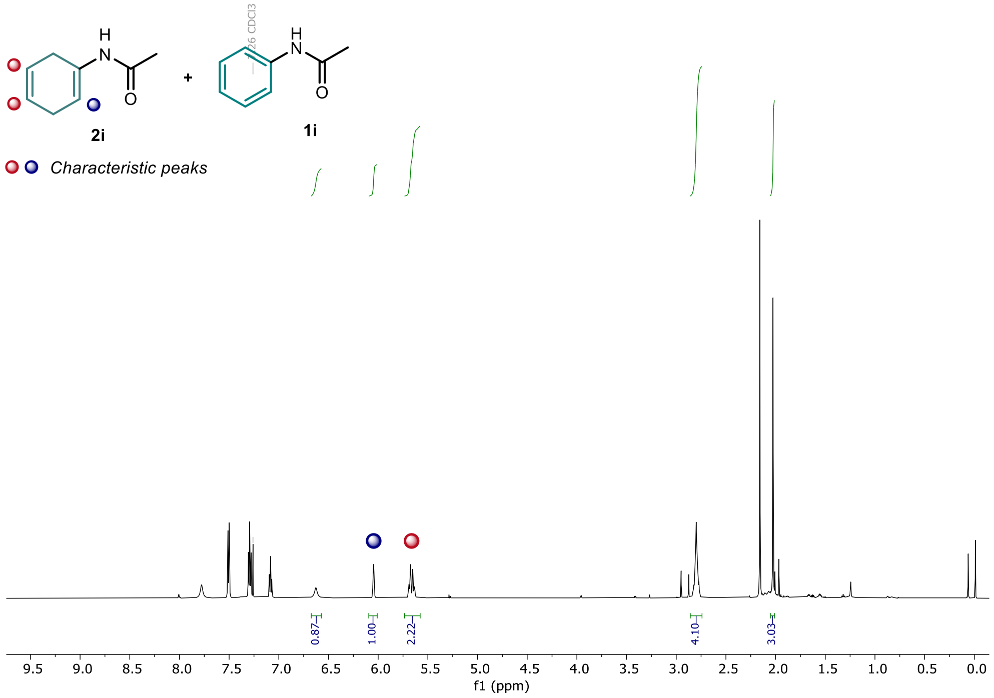

^{13}C NMR (151 MHz, $CDCl_3$)

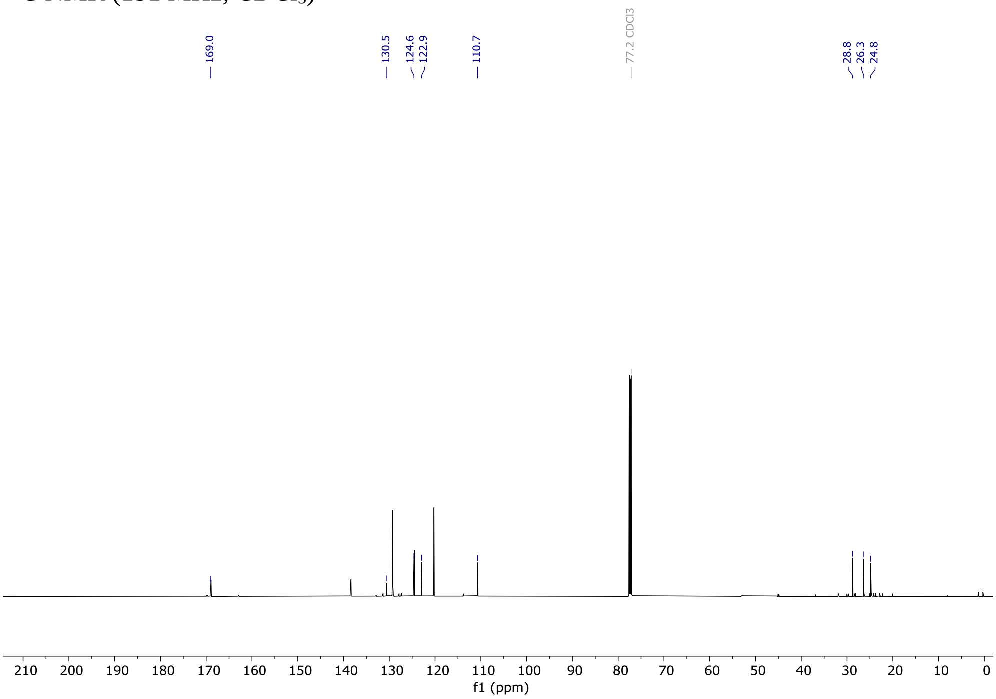

^{1}H NMR (600 MHz, $CDCl_3$)

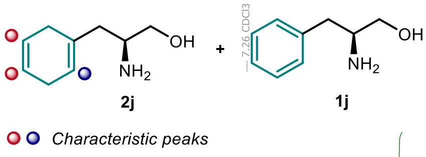

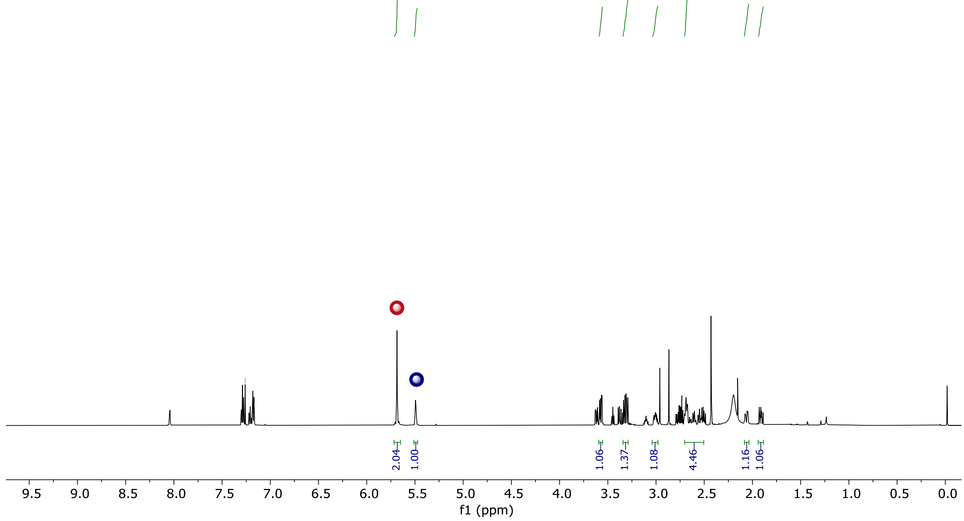

^{13}C NMR (151 MHz, $CDCl_3$)

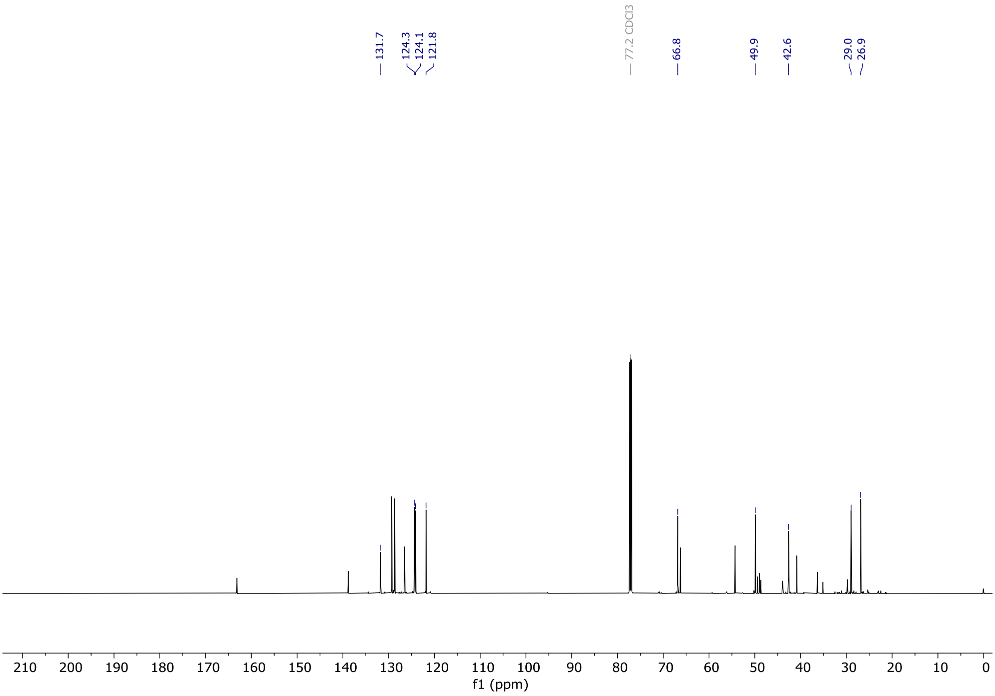

^{1}H NMR of the crude mixture in $CDCl_3$

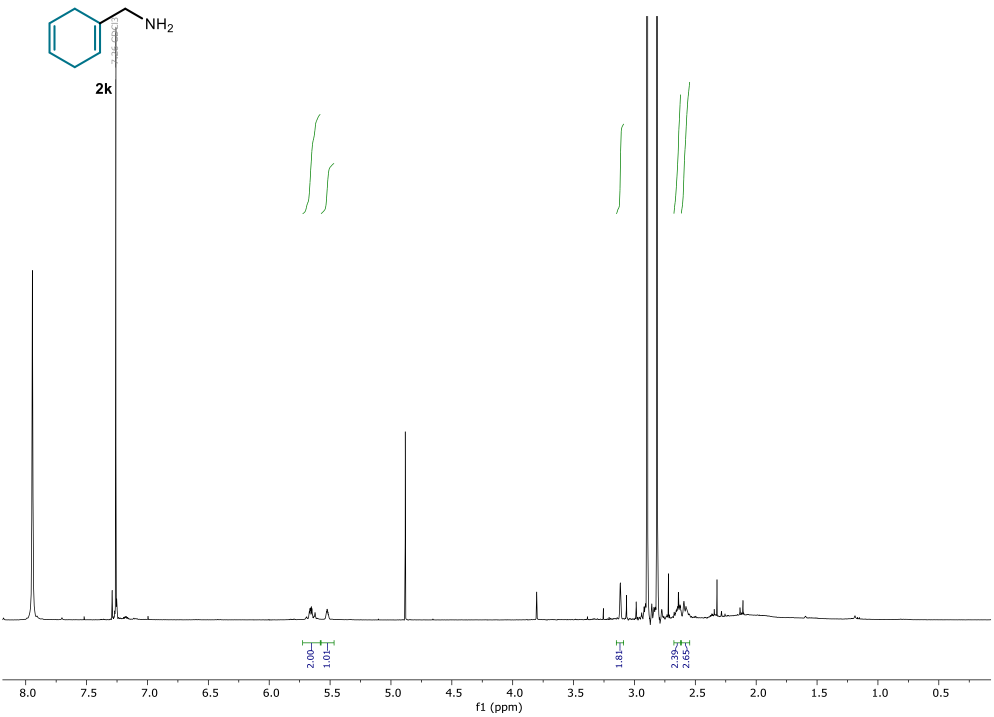

^{1}H NMR (600 MHz, $CDCl_3$)

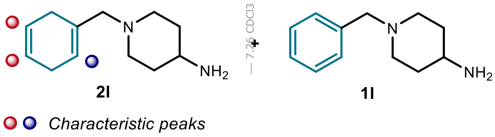

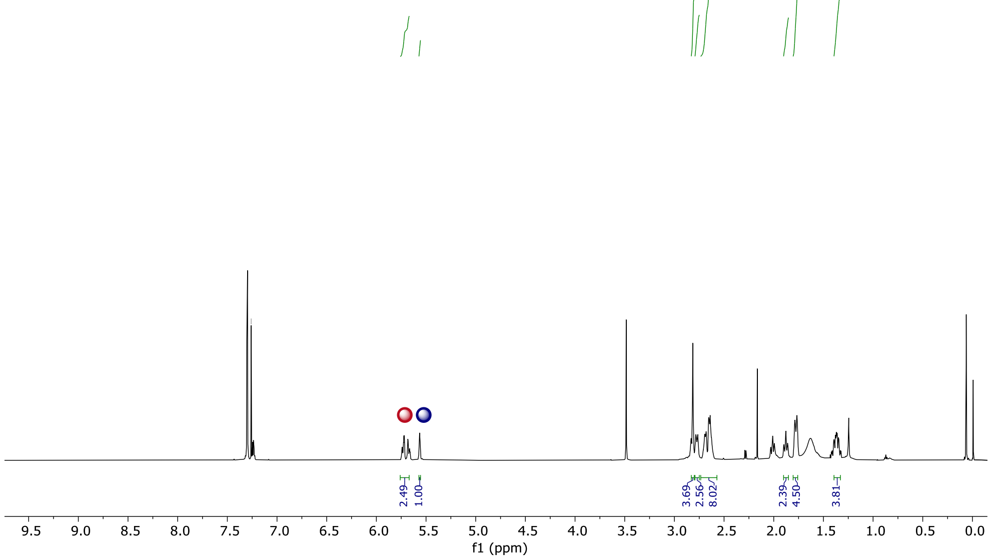

^{13}C NMR (151 MHz, $CDCl_3$)

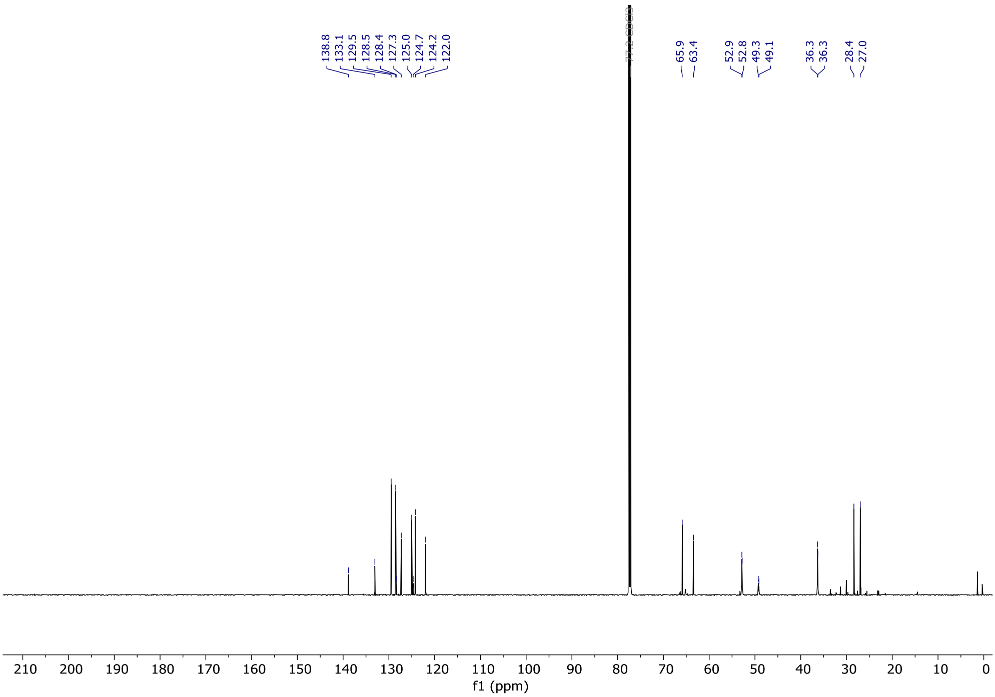

^{1}H NMR (500 MHz, $CDCl_3$)

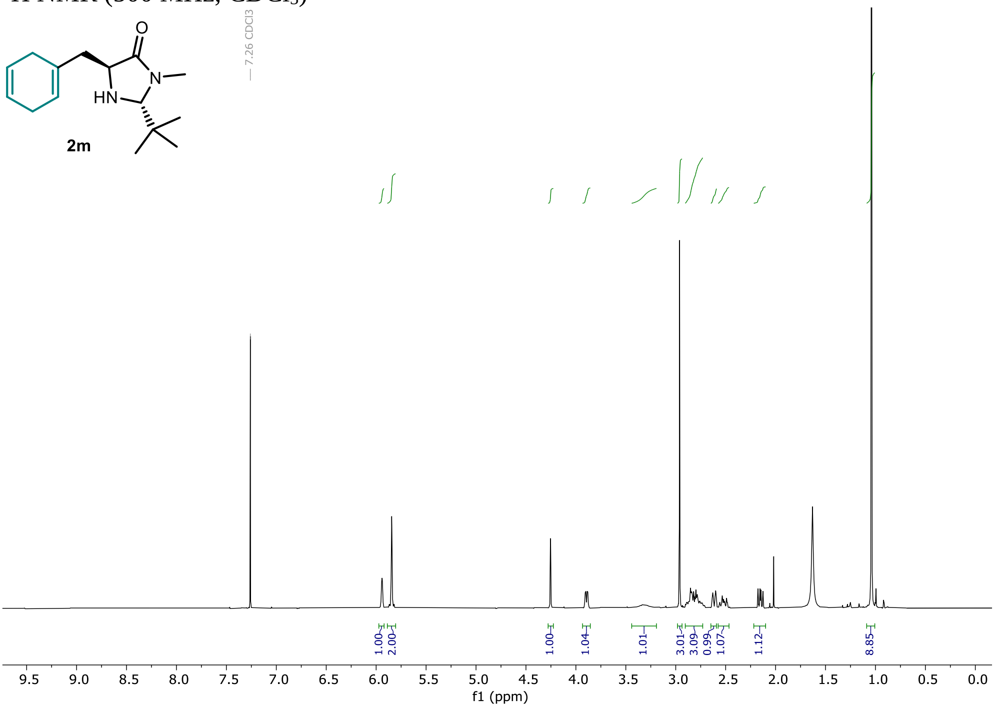

^{13}C NMR (126 MHz, $CDCl_3$)

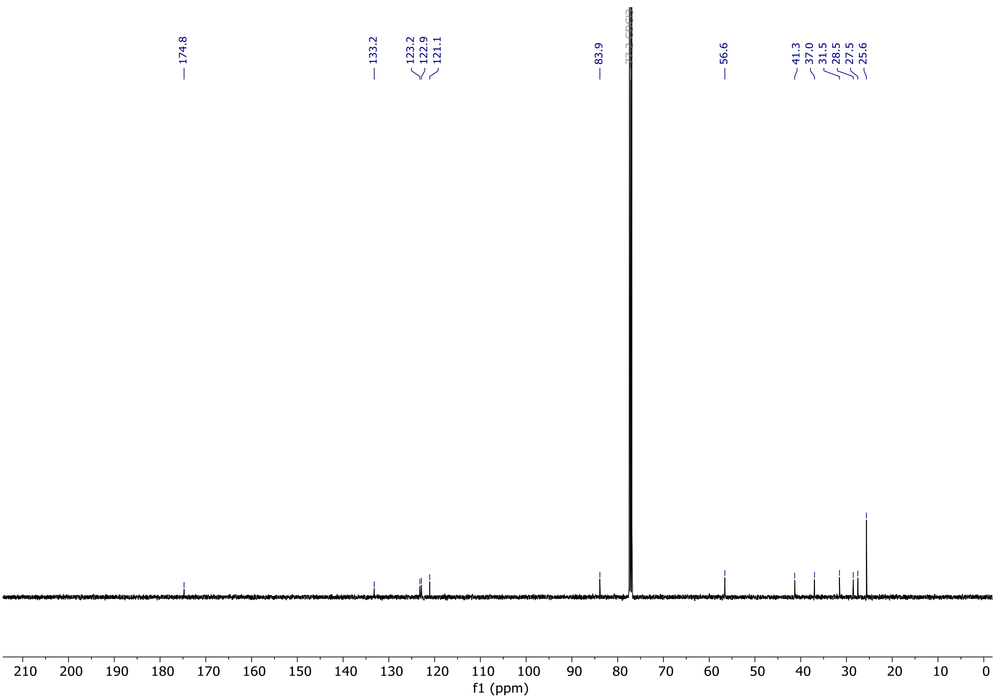

^{1}H NMR (600 MHz, $CDCl_3$)

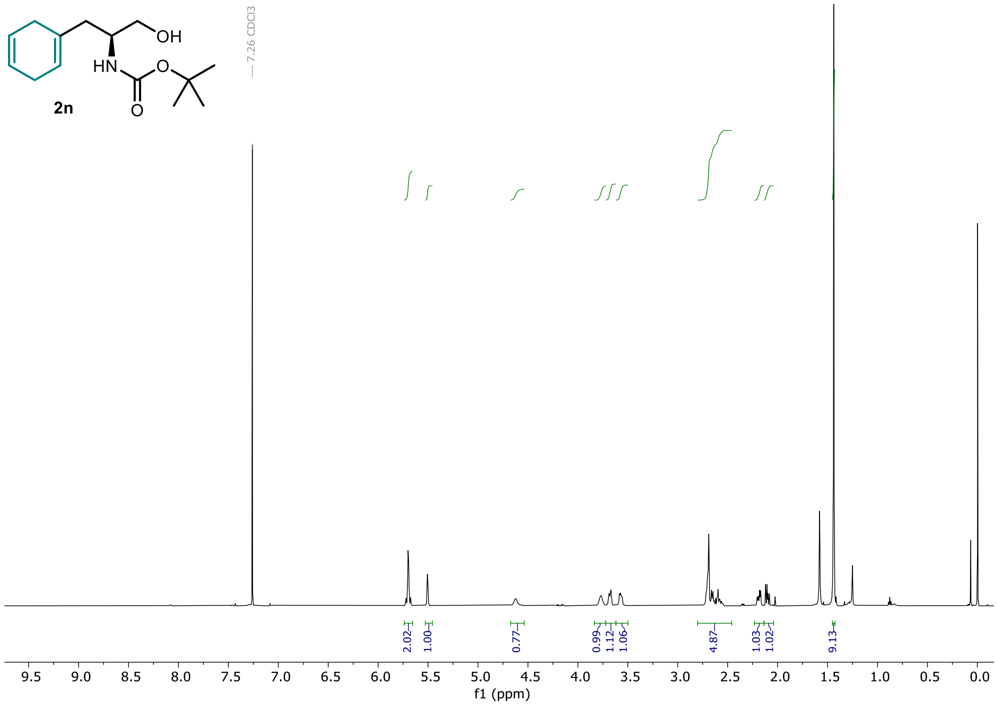

^{13}C NMR (151 MHz, $CDCl_3$)

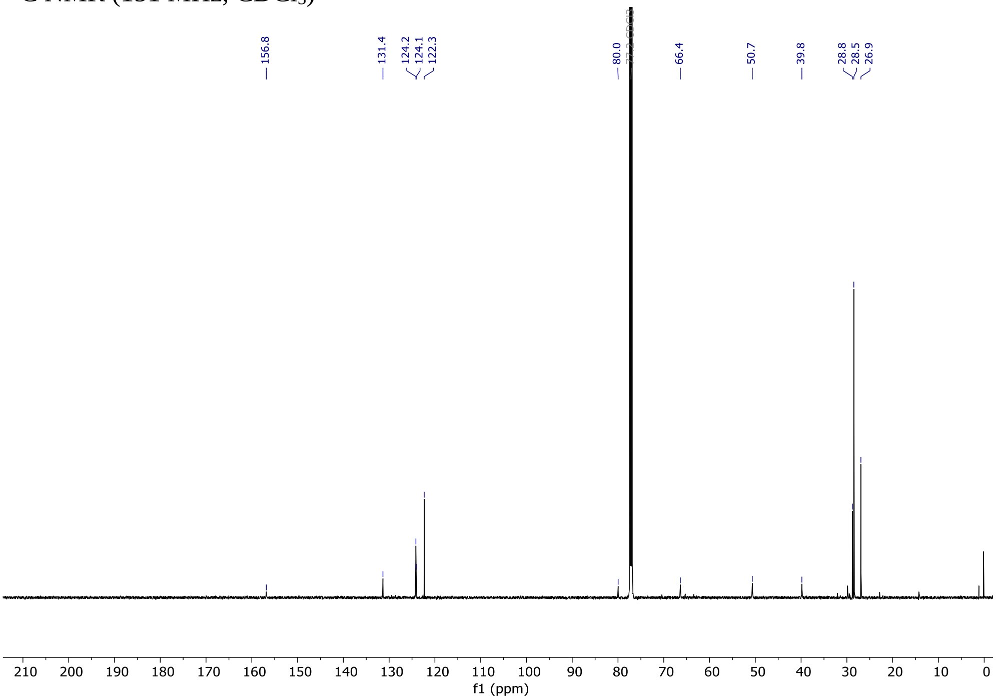

^{1}H NMR (500 MHz, $CDCl_3$)

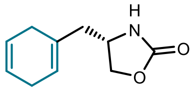

2o

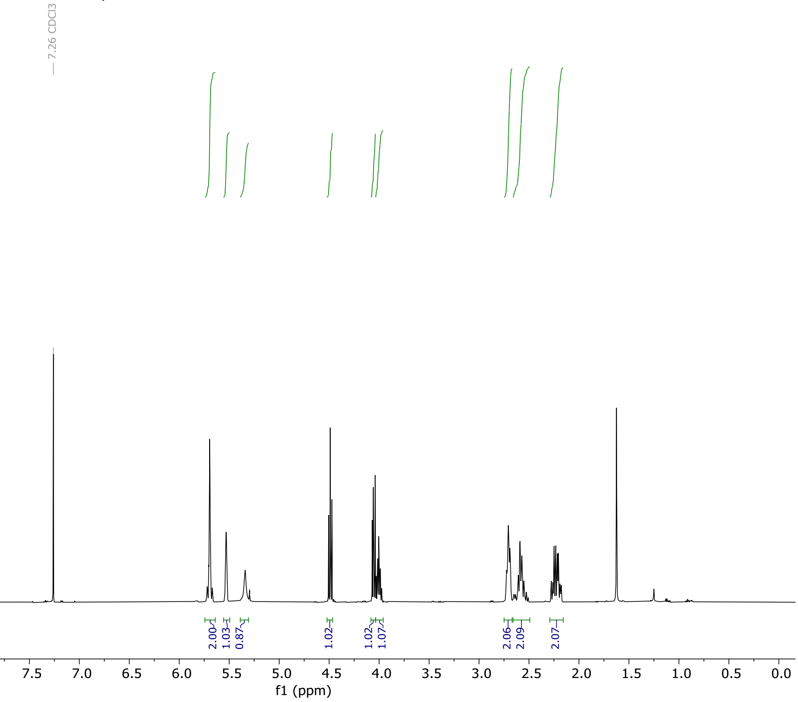

^{13}C NMR (126 MHz, $CDCl_3$)

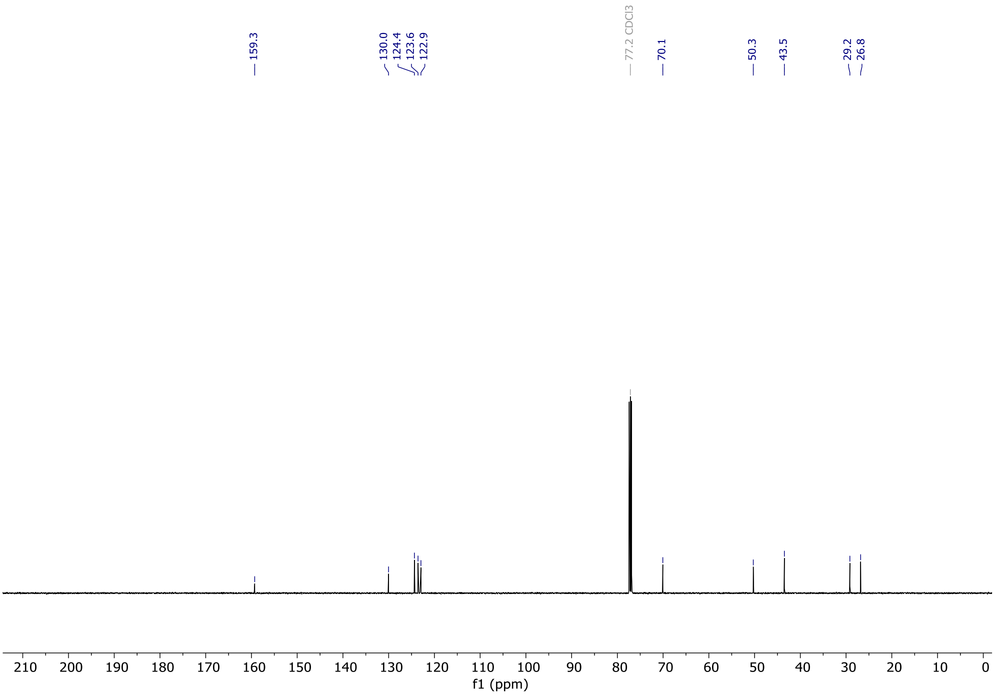

^{1}H NMR (600 MHz, $CDCl_3$)

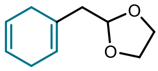

2p

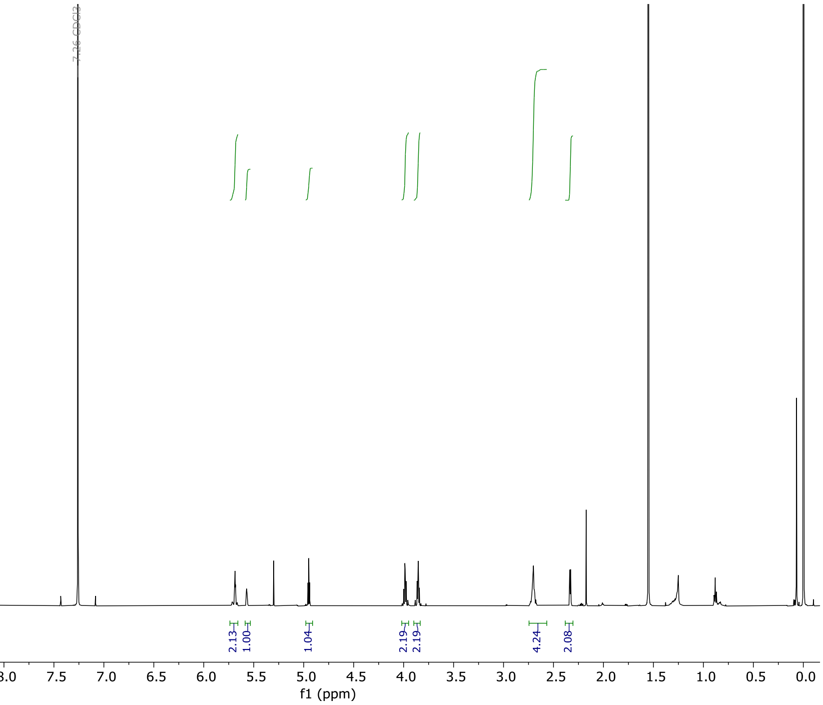

^{13}C NMR (151 MHz, $CDCl_3$)

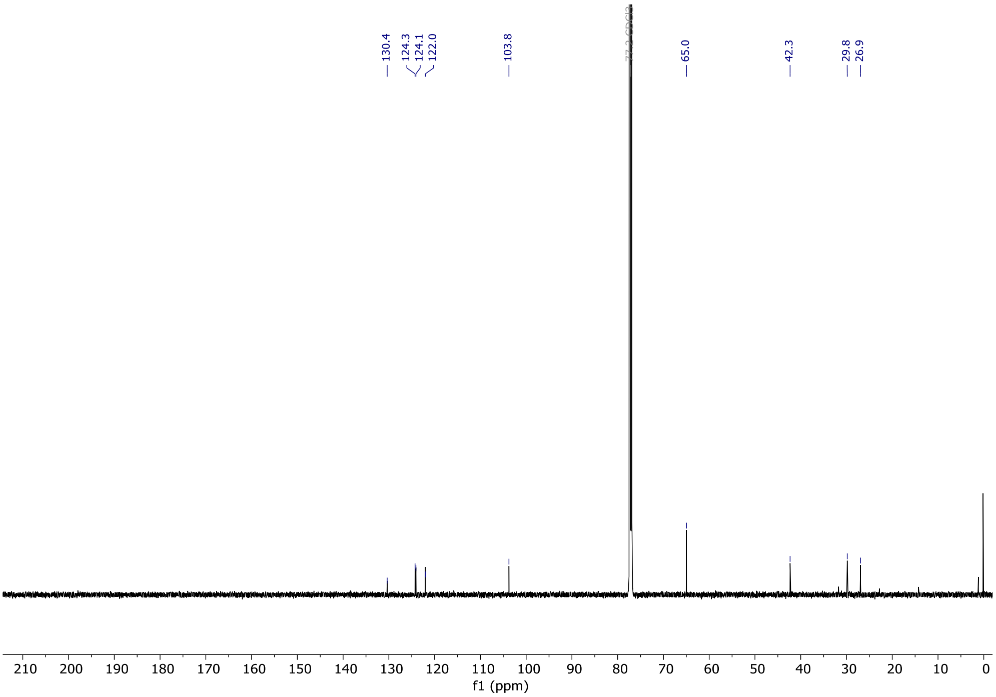

^{1}H NMR (600 MHz, $CDCl_3$)

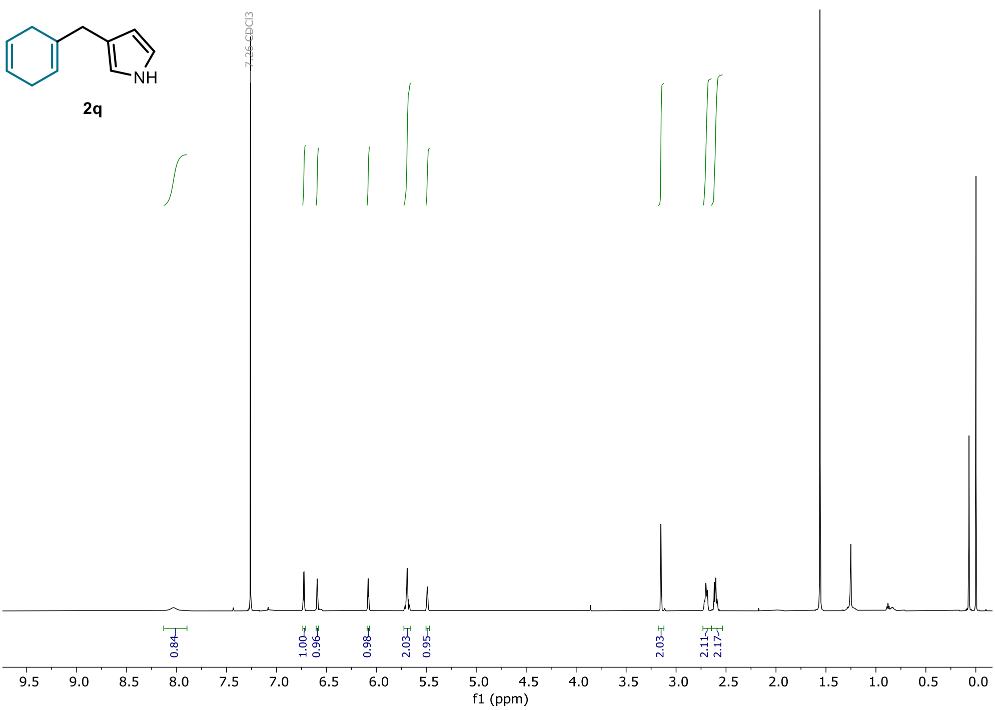

^{13}C NMR (151 MHz, $CDCl_3$)

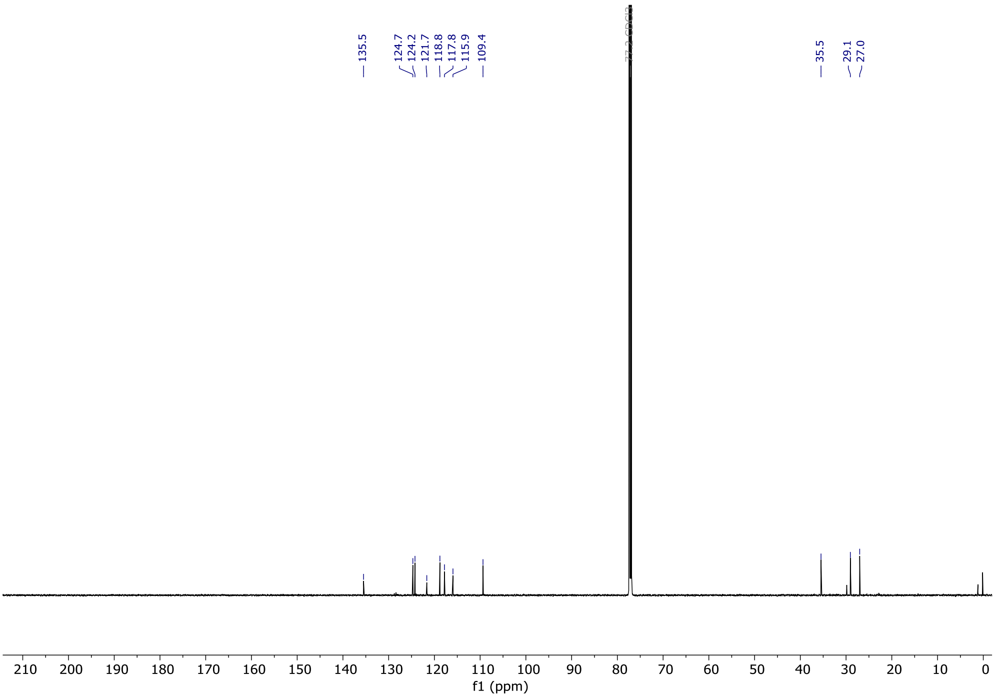

^{1}H NMR (600 MHz, $CDCl_3$)

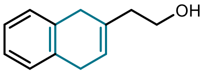

2r

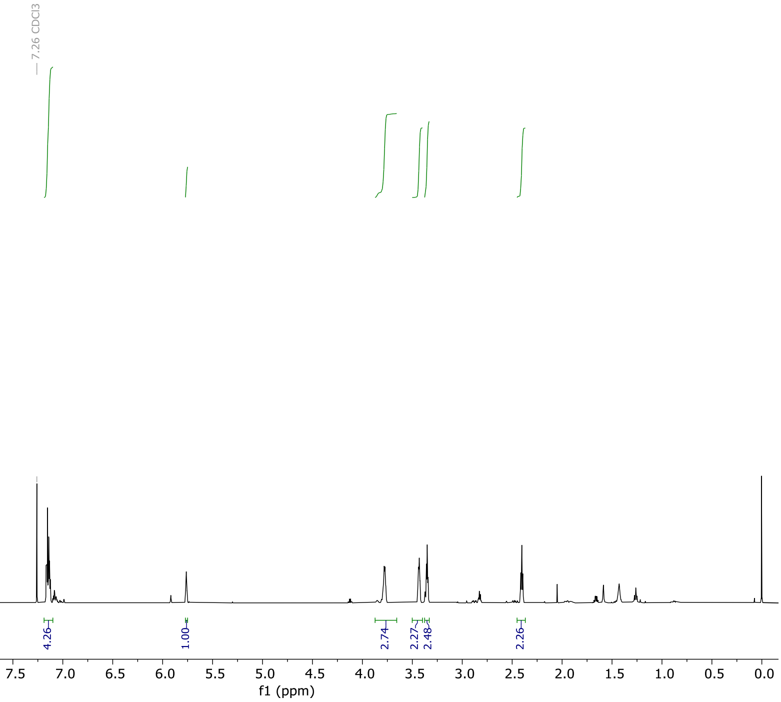

^{13}C NMR (151 MHz, $CDCl_3$)

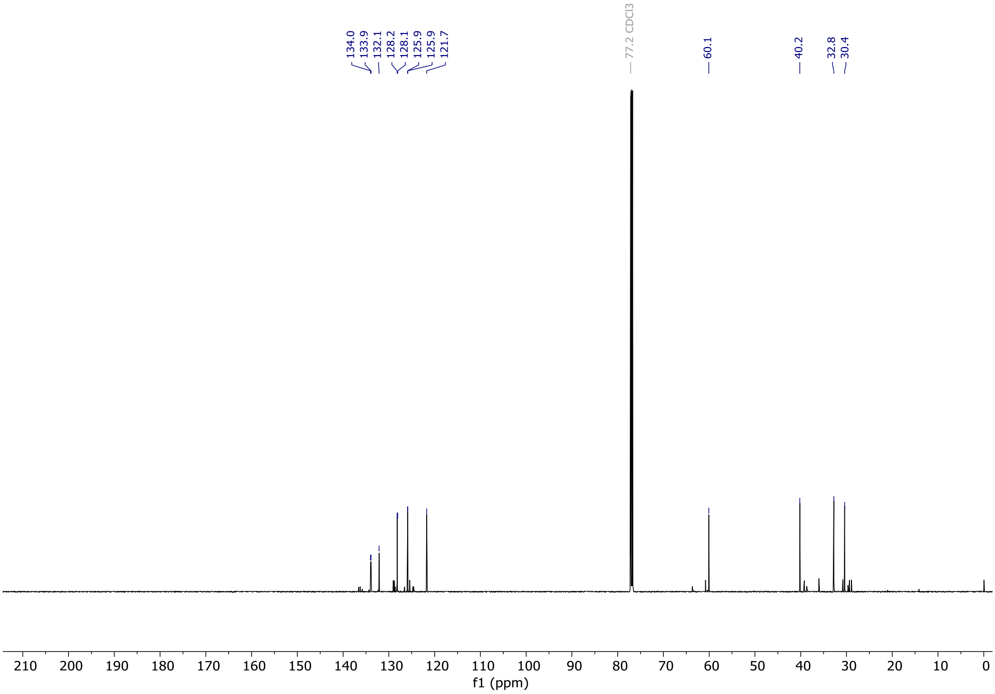

^{1}H NMR (600 MHz, $CDCl_3$)

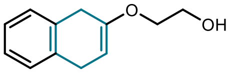

2s

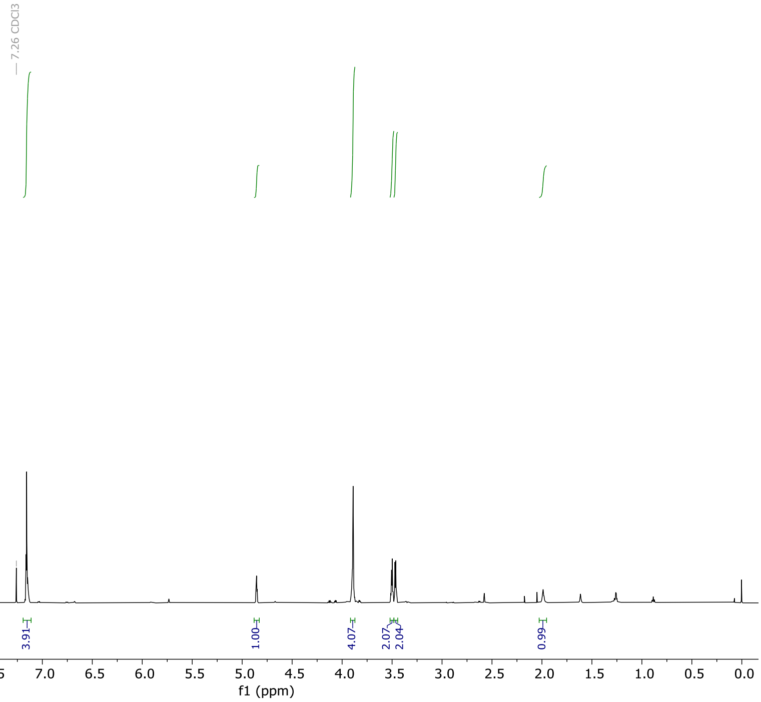

^{13}C NMR (151 MHz, $CDCl_3$)

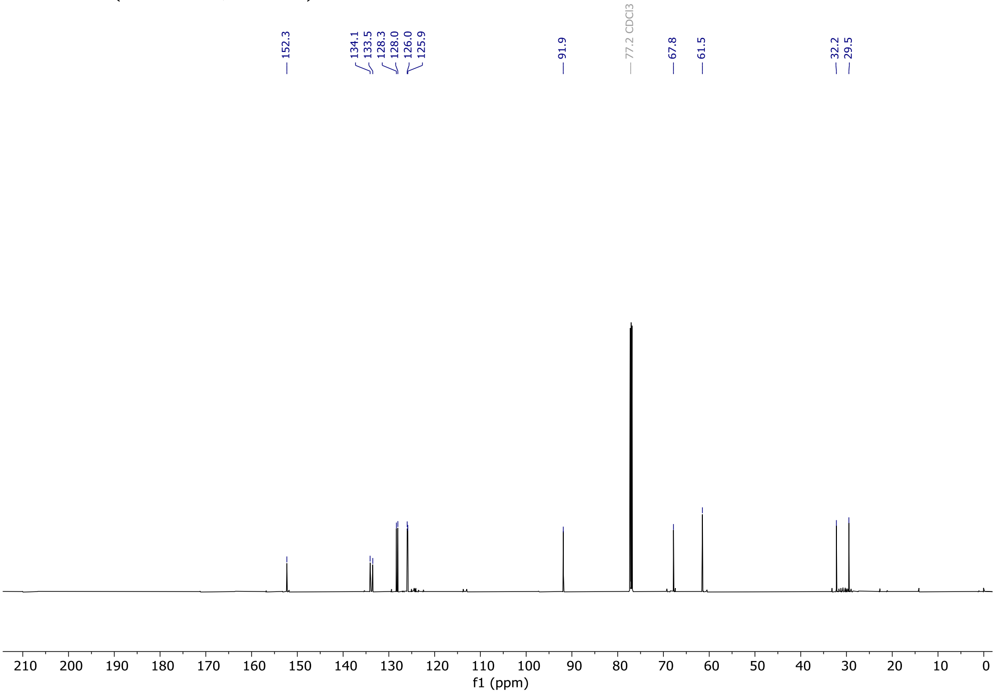

^{1}H NMR (600 MHz, CDCl$_3$)

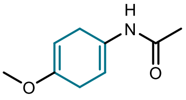

2t

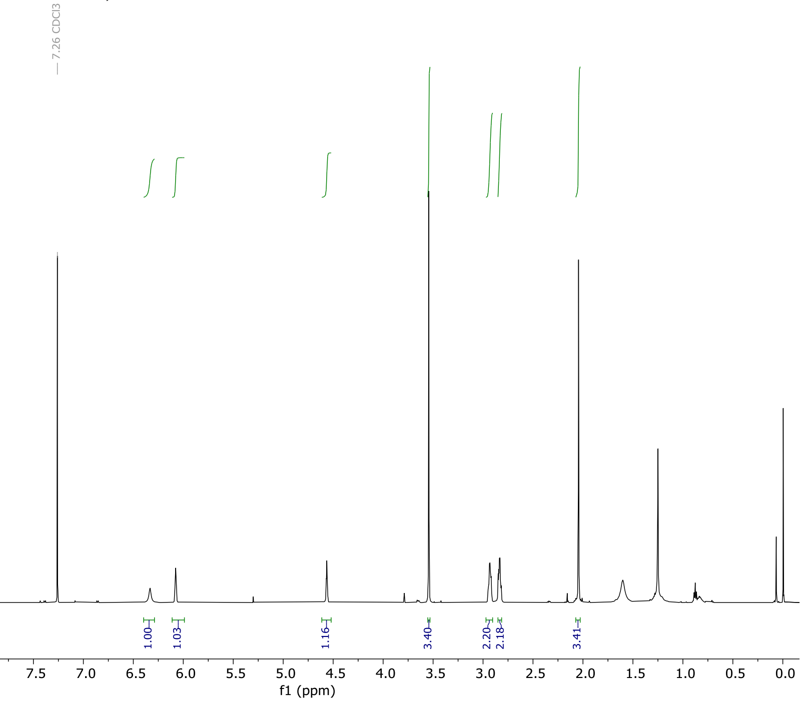

^{13}C NMR (151 MHz, CDCl$_3$)

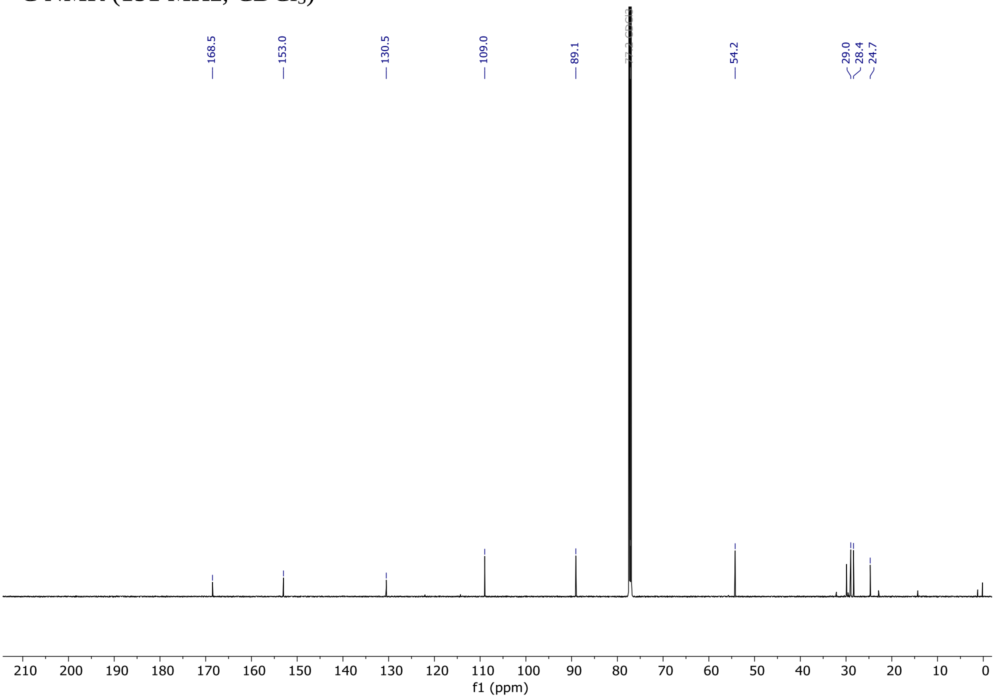

^{1}H NMR (600 MHz, DMSO-d_6)

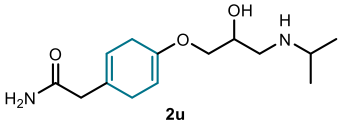

2u

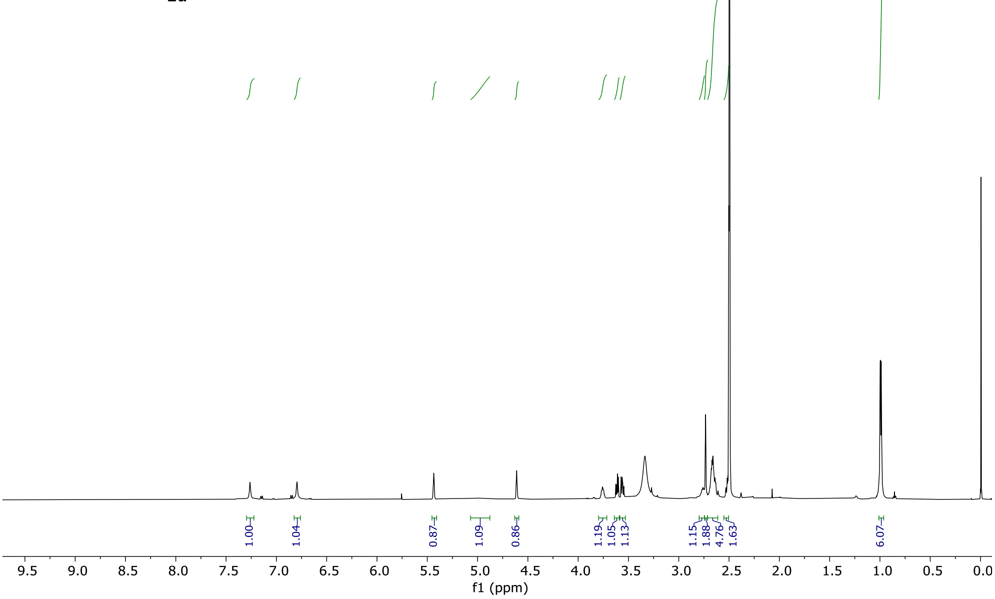

^{13}C NMR (151 MHz, DMSO-d_6)

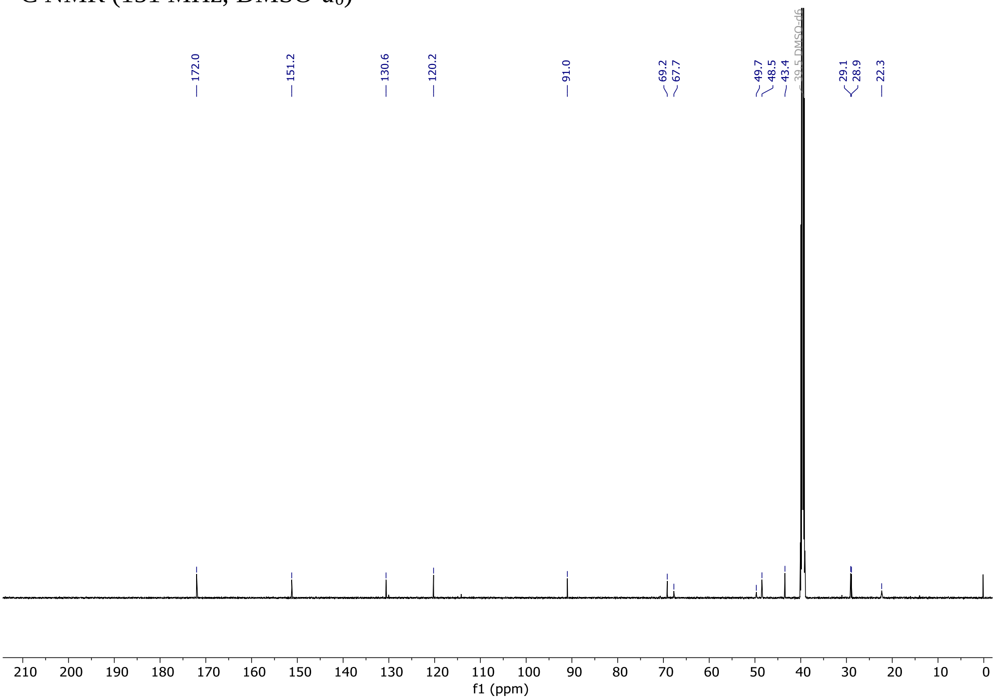

^{1}H NMR (600 MHz, $CDCl_3$)

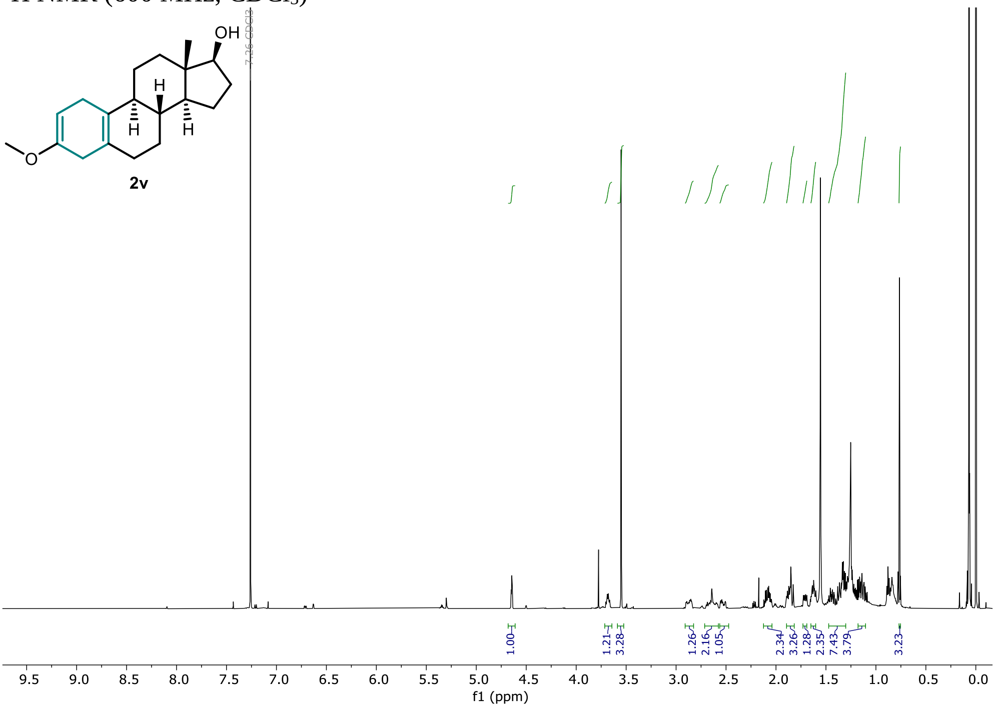

2v

^{13}C NMR (151 MHz, $CDCl_3$)

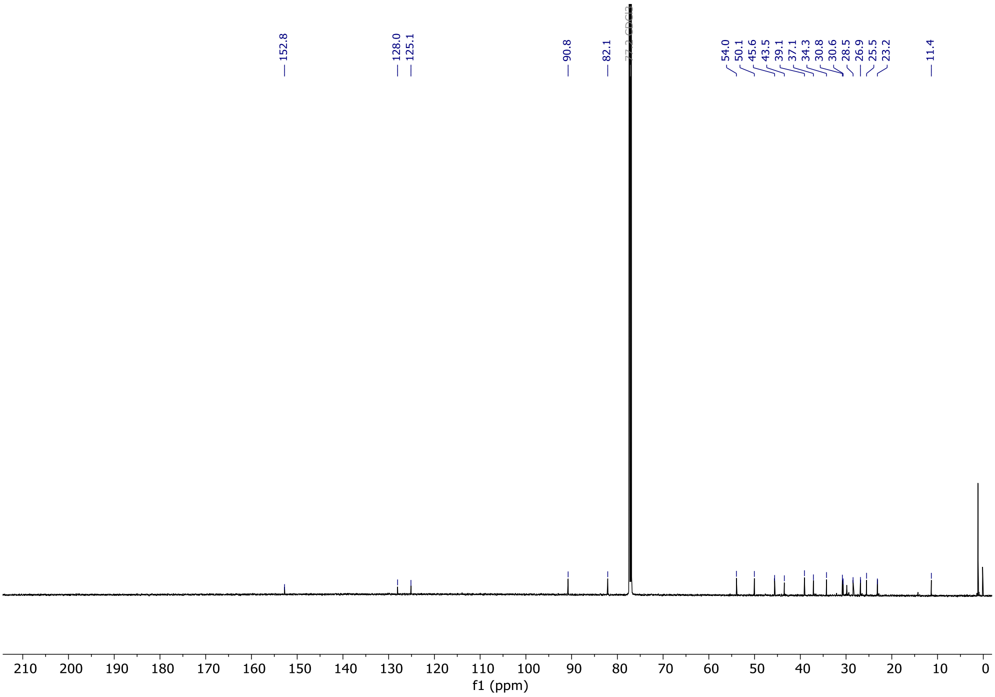

^{13} NMR (400 MHz, $CDCl_3$)

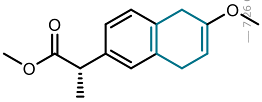

2w

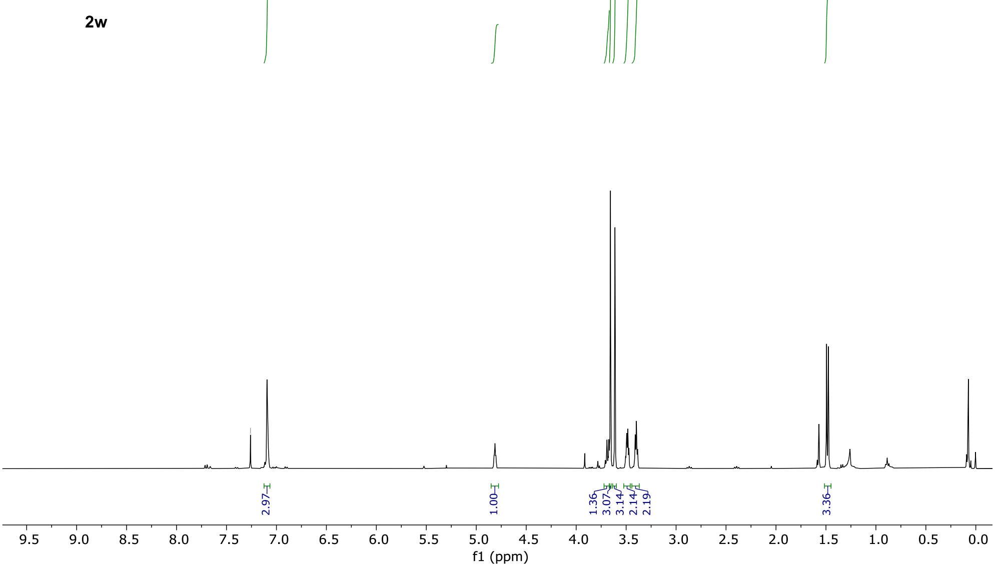

^{13}C NMR (101 MHz, $CDCl_3$)

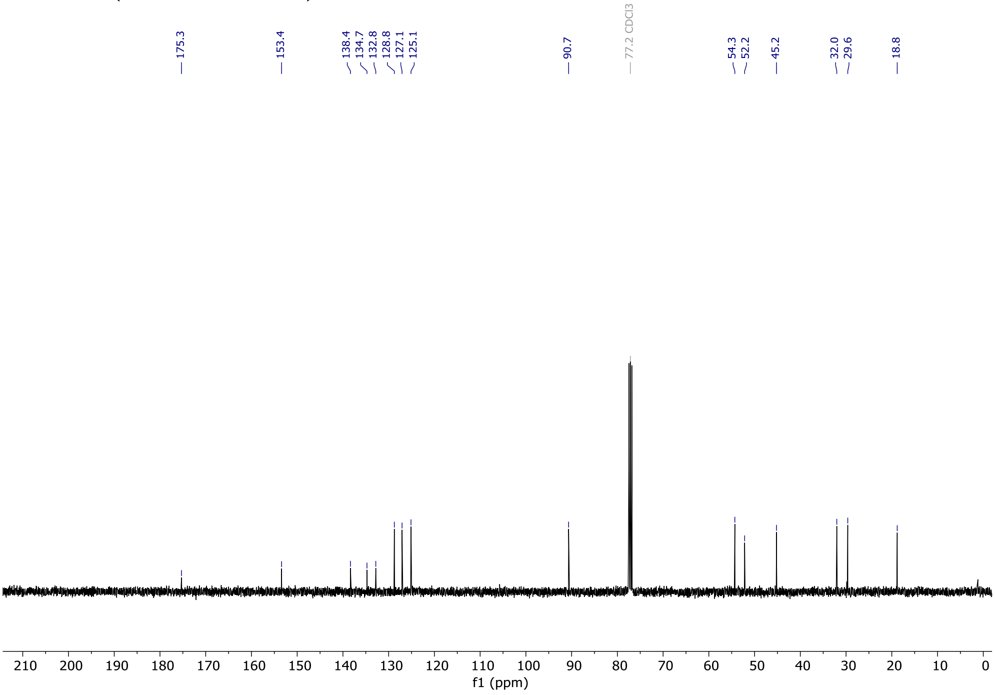

^{1}H NMR (400 MHz, $CDCl_3$)

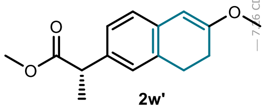

2w'

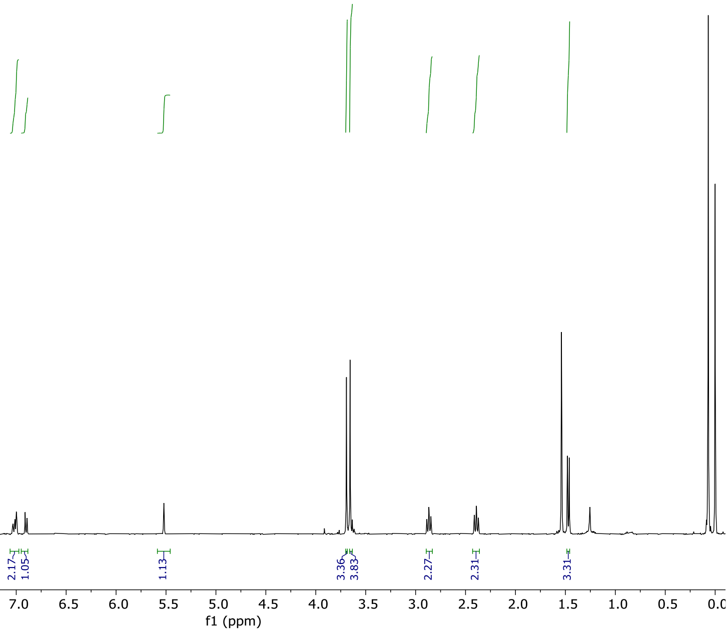

^{13}C NMR (151MHz, $CDCl_3$)

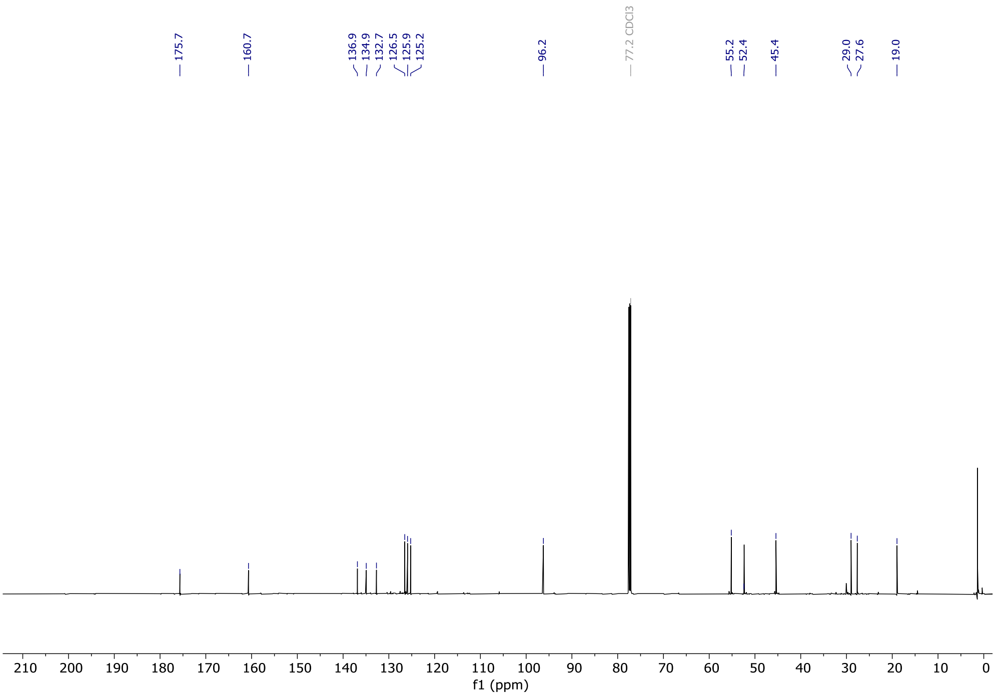

^{1}H NMR (600 MHz, CDCl3)

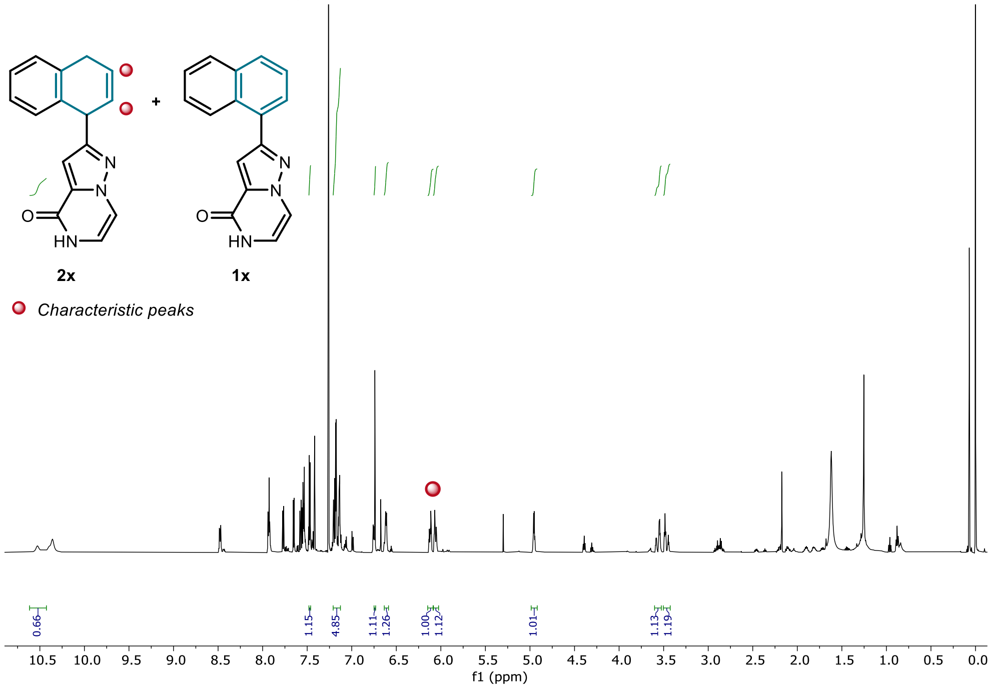

^{13}C NMR (151 MHz, CDCl3)

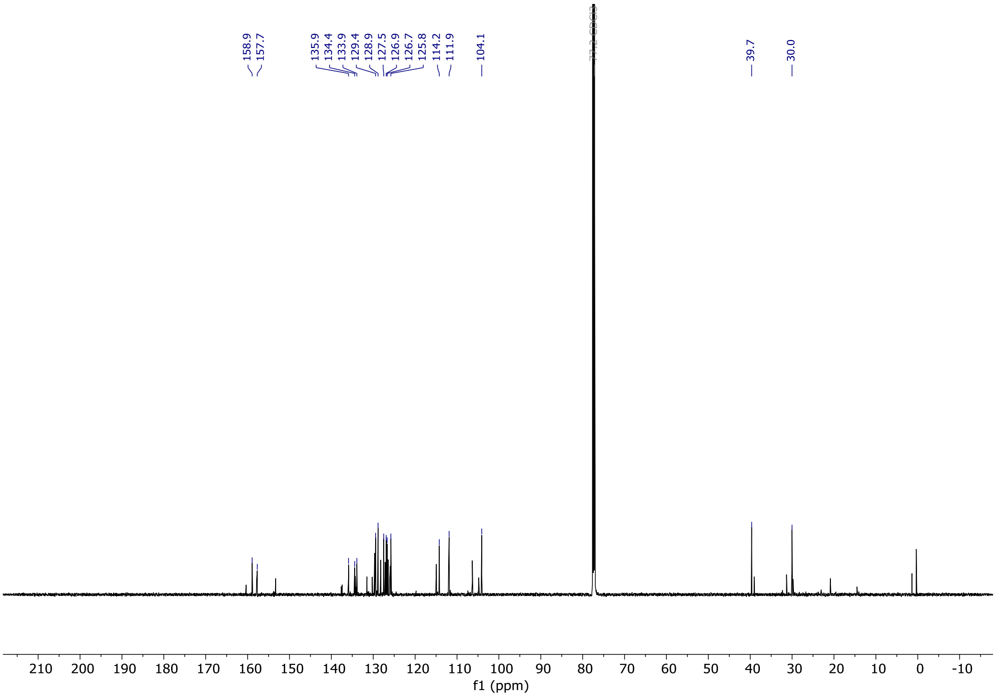

^{1}H NMR of the crude mixture in $CDCl_3$

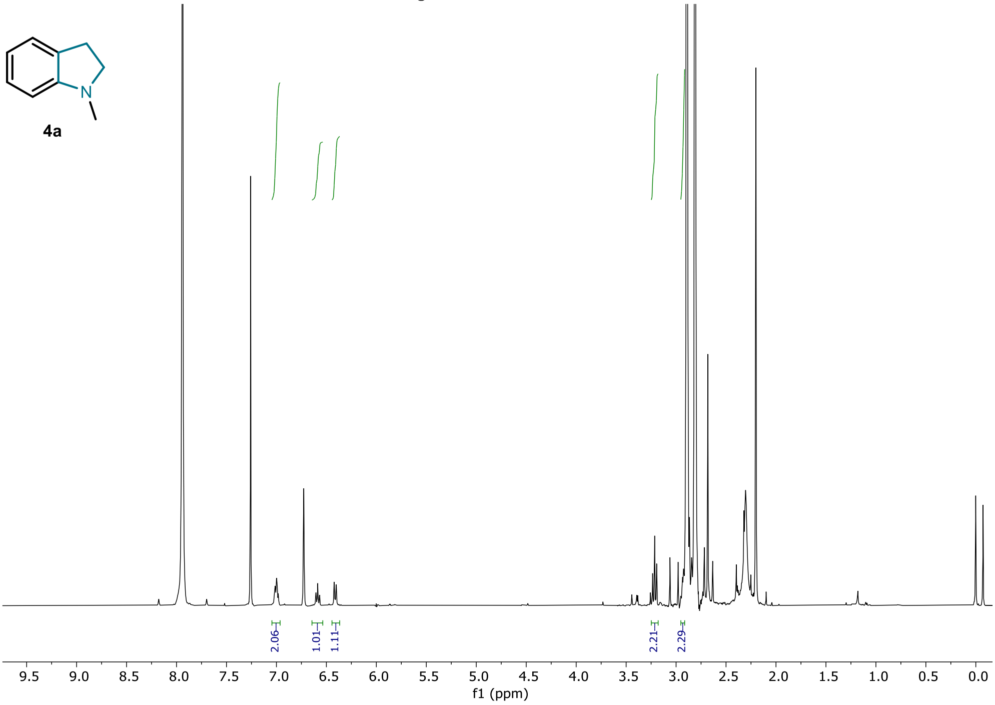

^{1}H NMR (500 MHz, $CDCl_3$)

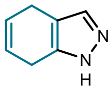

4b

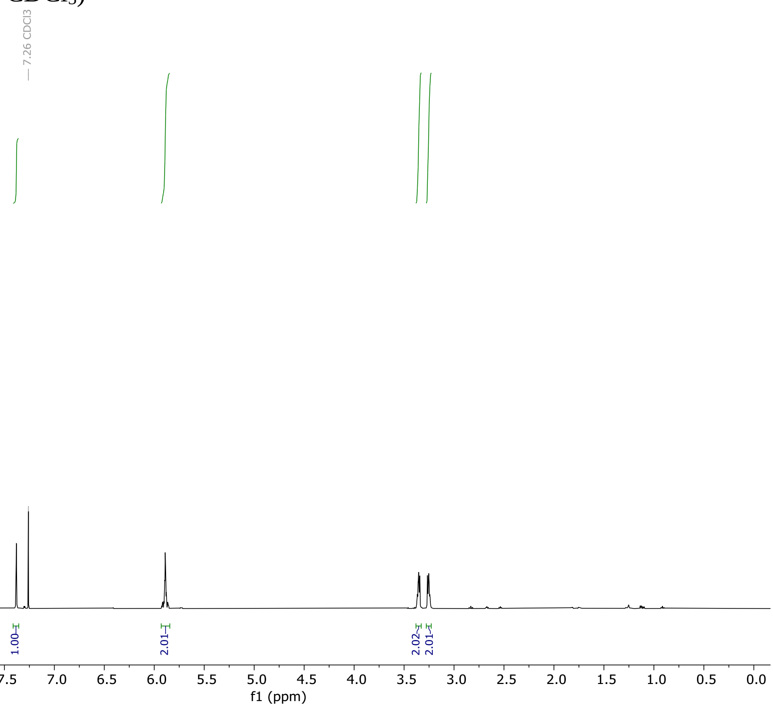

^{13}C NMR (126 MHz, $CDCl_3$)

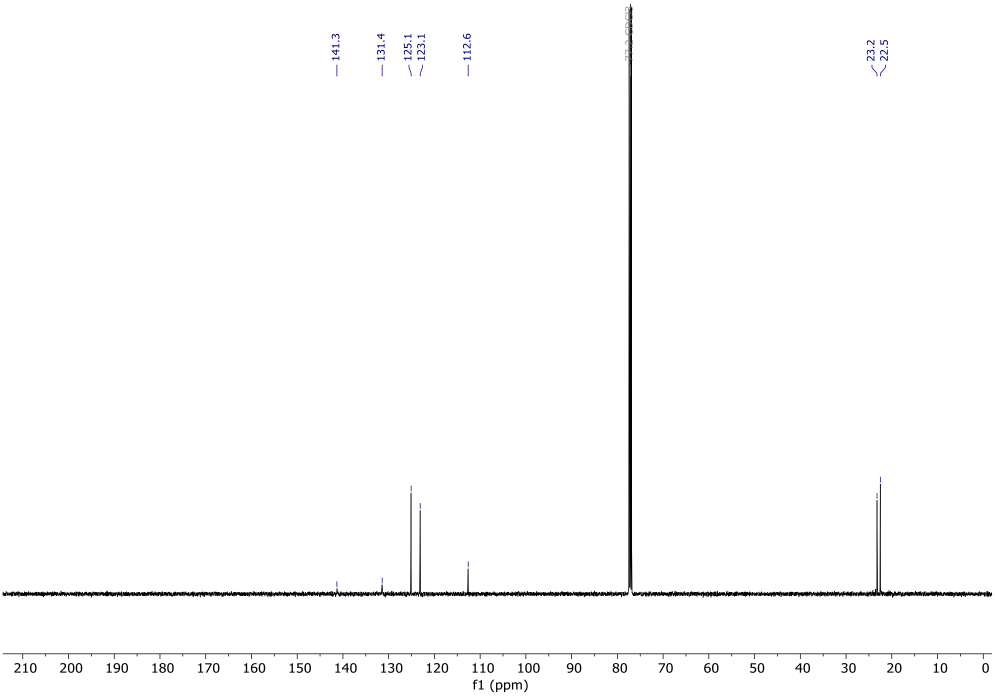

^{1}H NMR (600 MHz, $CDCl_3$)

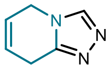

4c

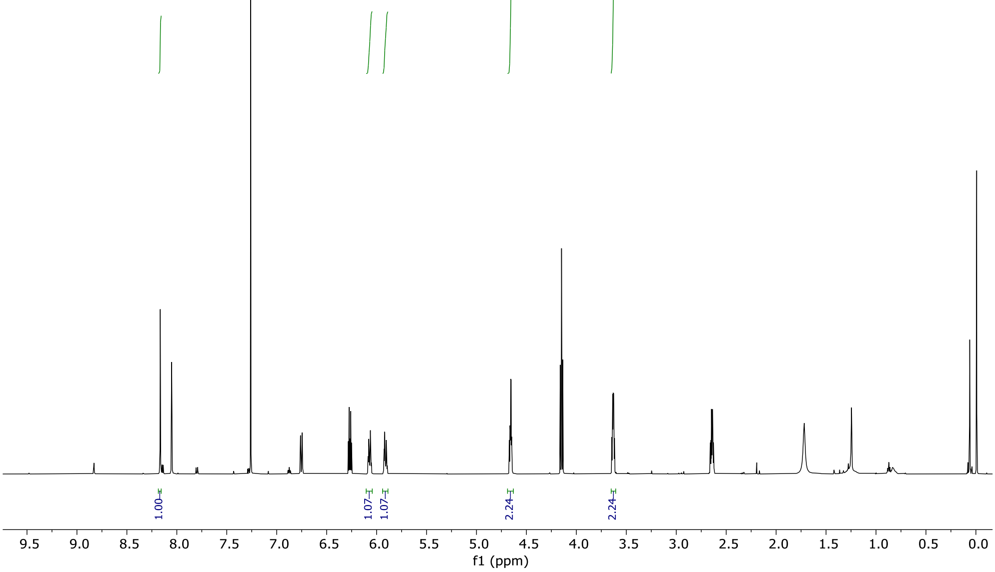

^{1}H NMR (600 MHz, $CDCl_3$)

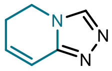

4c'

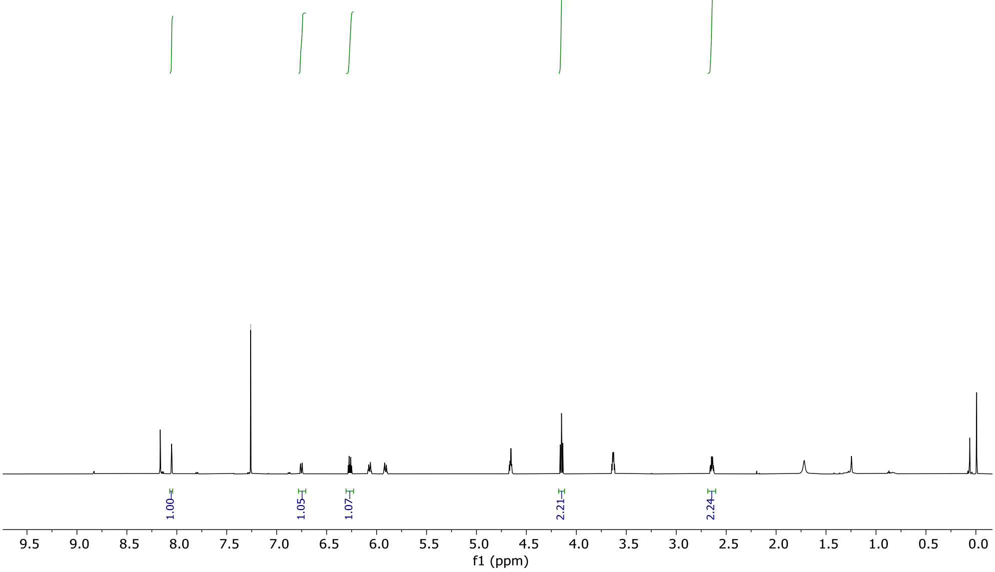

^{13}C NMR (151 MHz, $CDCl_3$)

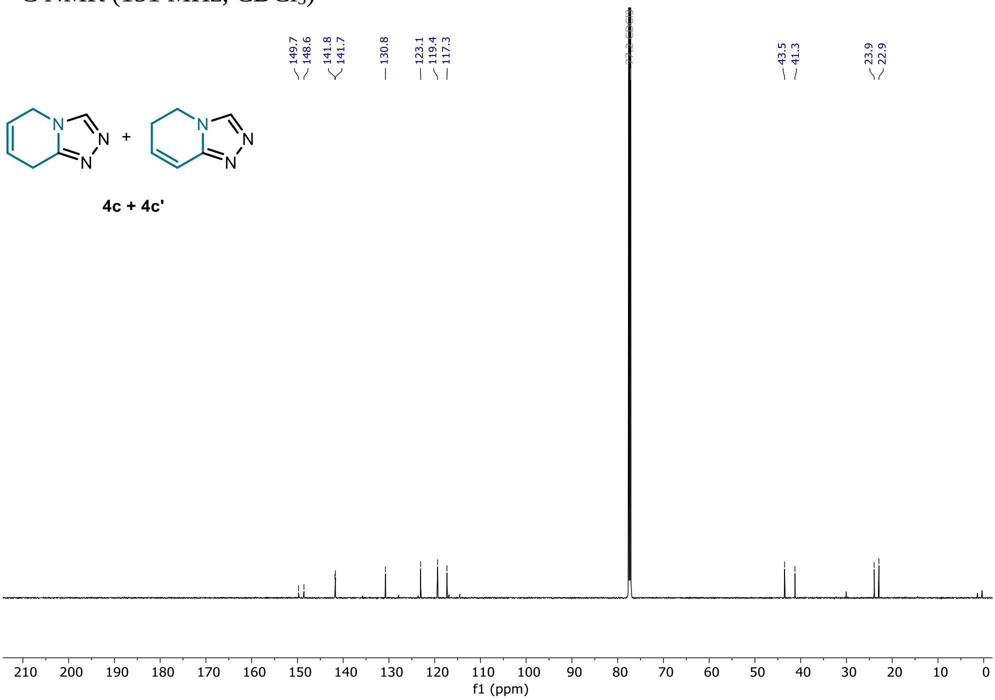

^{1}H NMR (600 MHz, $CDCl_3$)

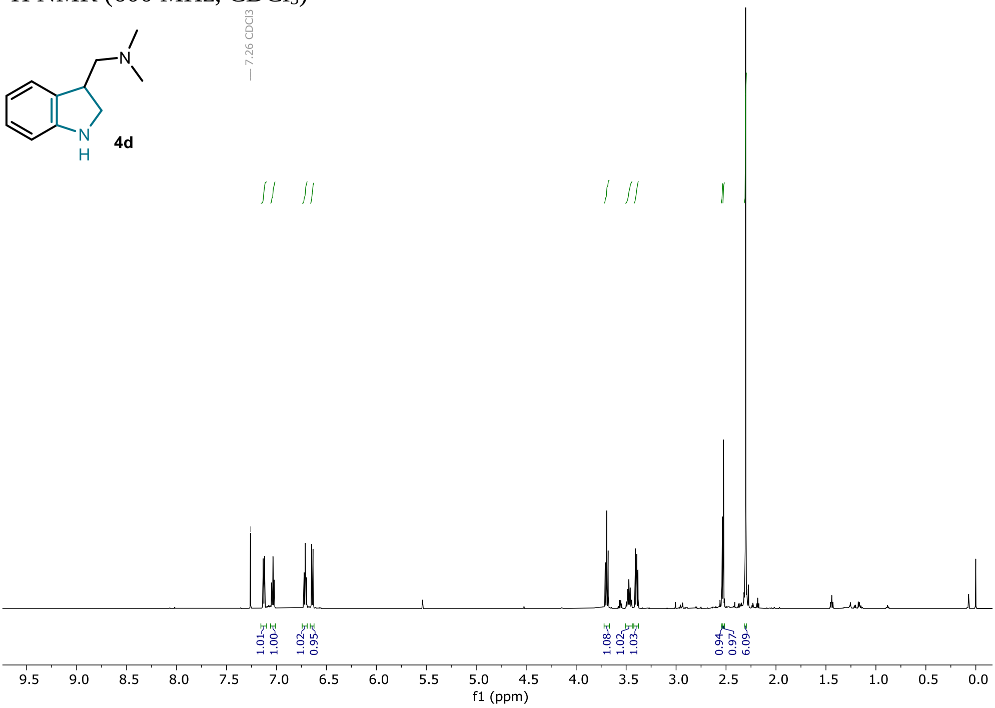

^{13}C NMR (151 MHz, $CDCl_3$)

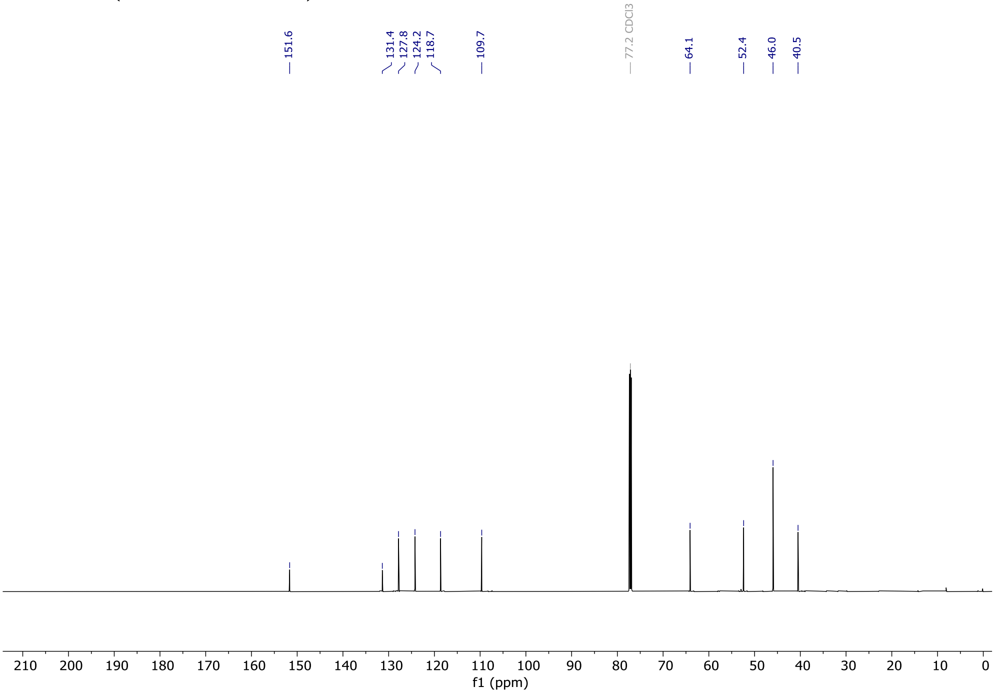

^{1}H NMR (600 MHz, $CDCl_3$)

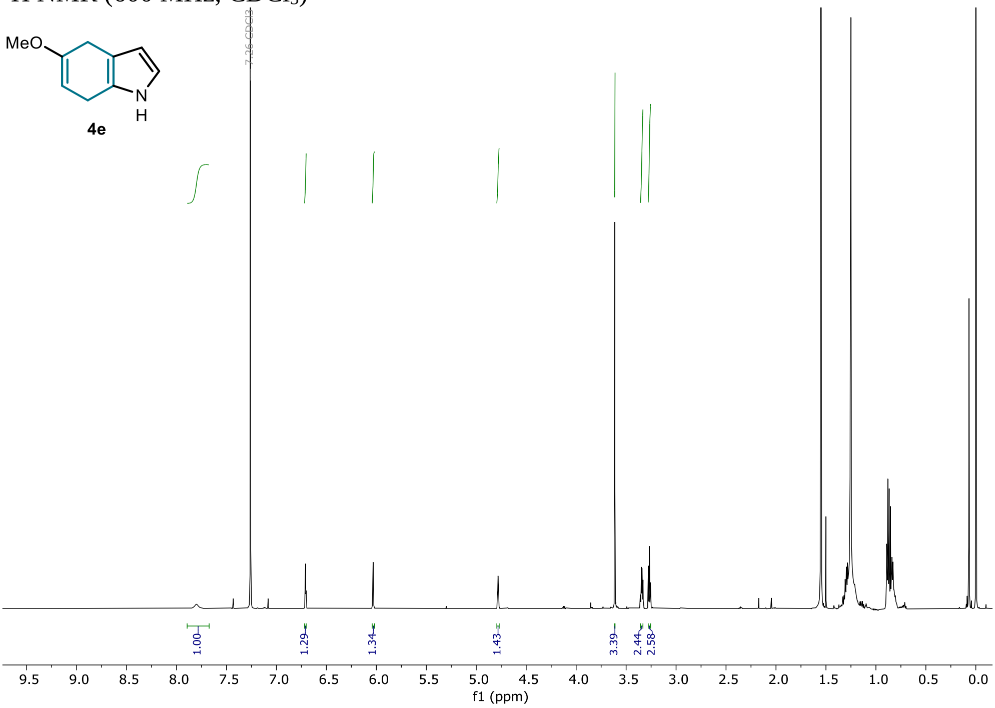

^{13}C NMR (151 MHz, $CDCl_3$)

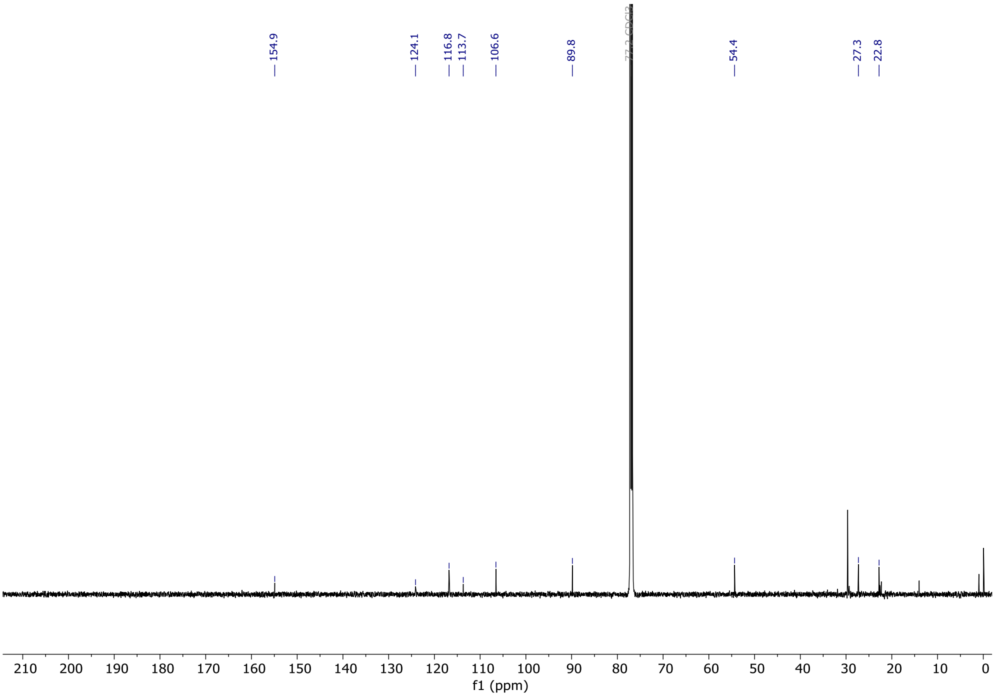

^{1}H NMR (600 MHz, $CDCl_3$)

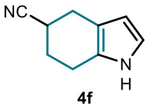

4f

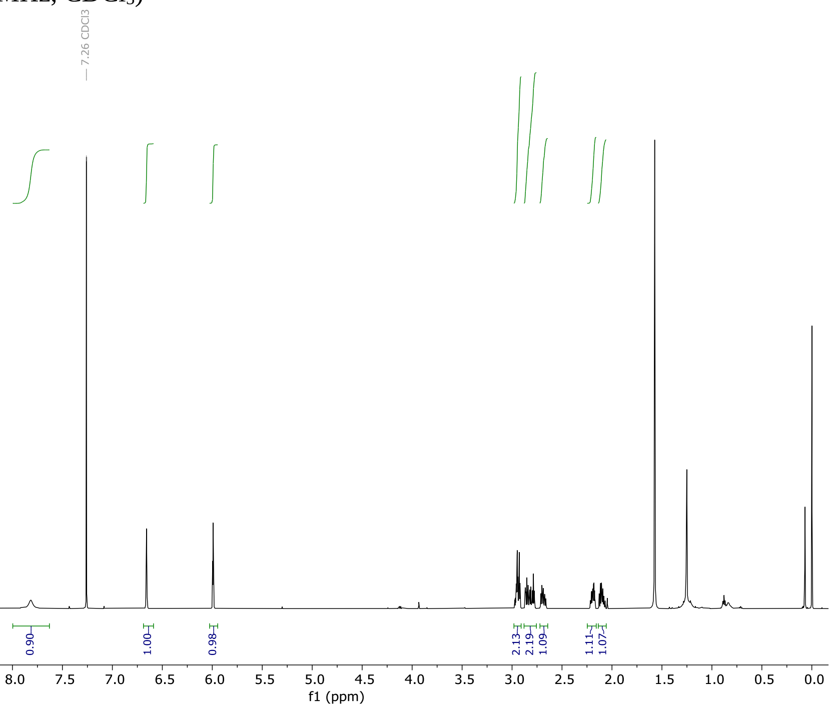

^{13}C NMR (151 MHz, $CDCl_3$)

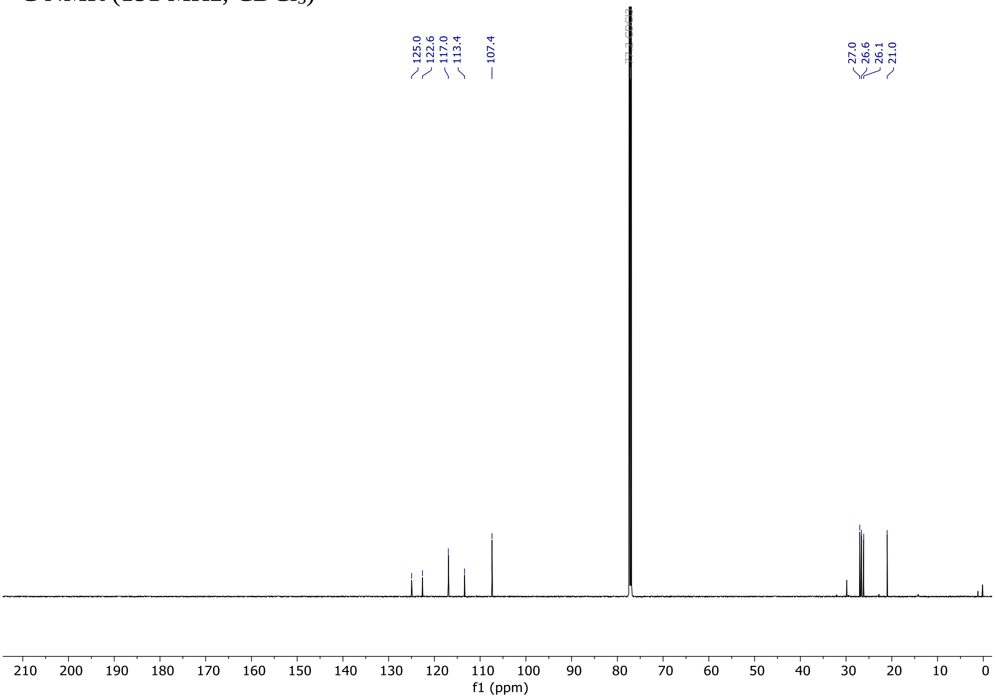

^{1}H NMR (600 MHz, $CDCl_3$)

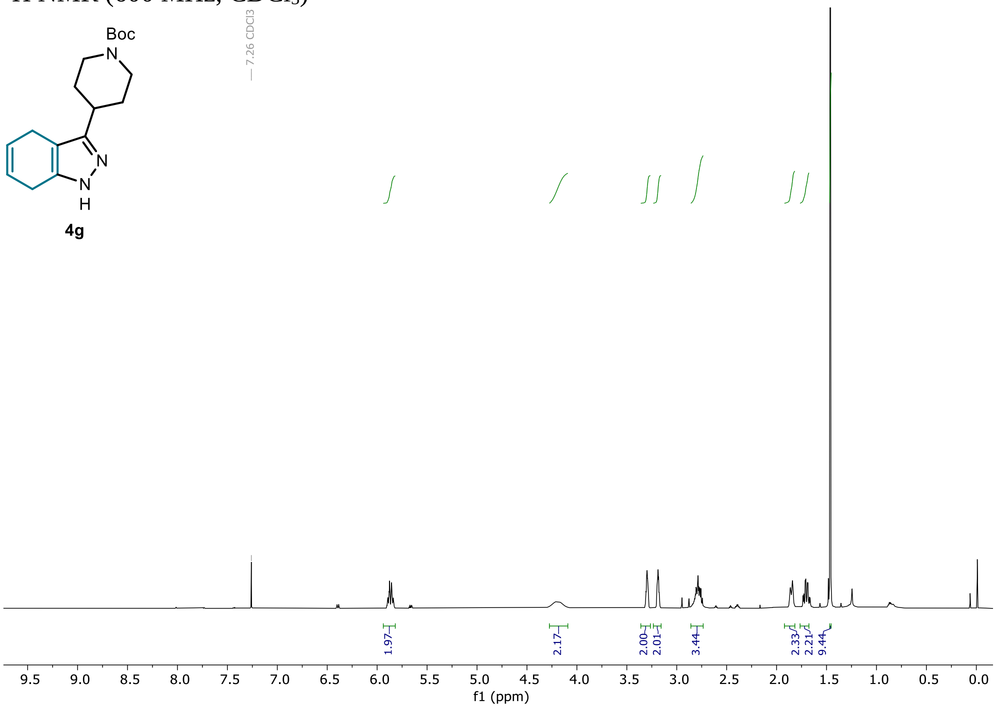

^{13}C NMR (151 MHz, $CDCl_3$)

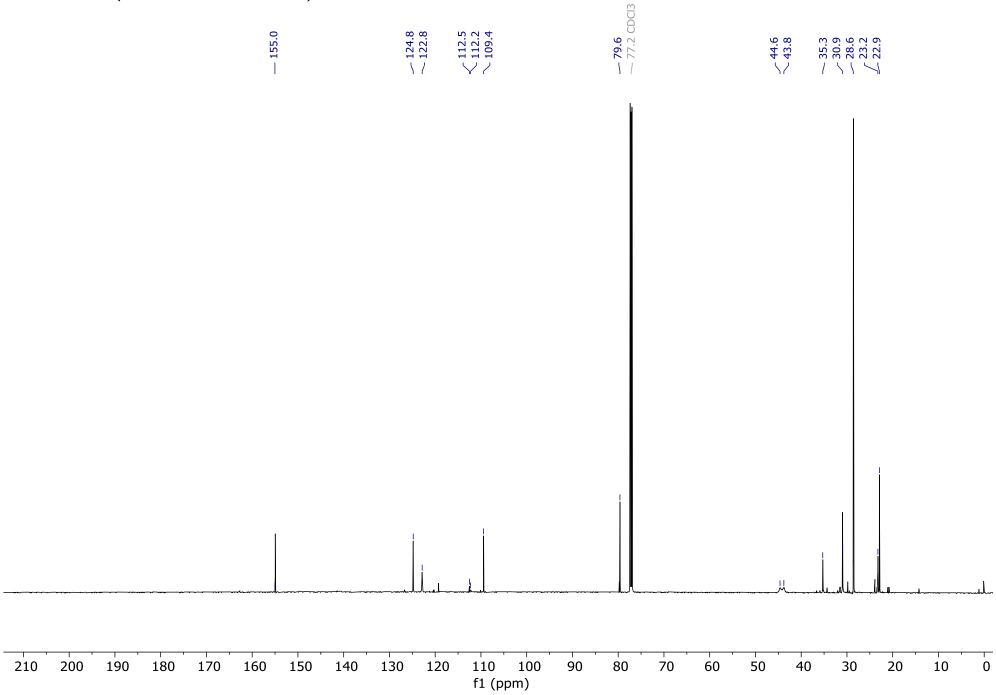

^{1}H NMR of the crude mixture in $CDCl_3$

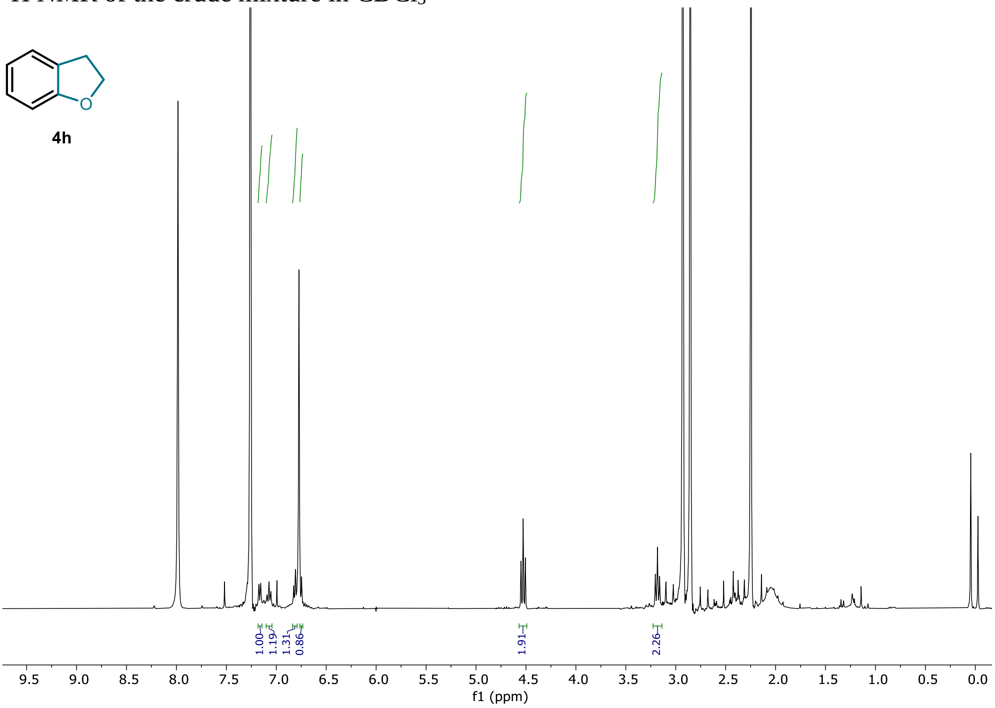

^{1}H NMR of the crude mixture in $CDCl_3$

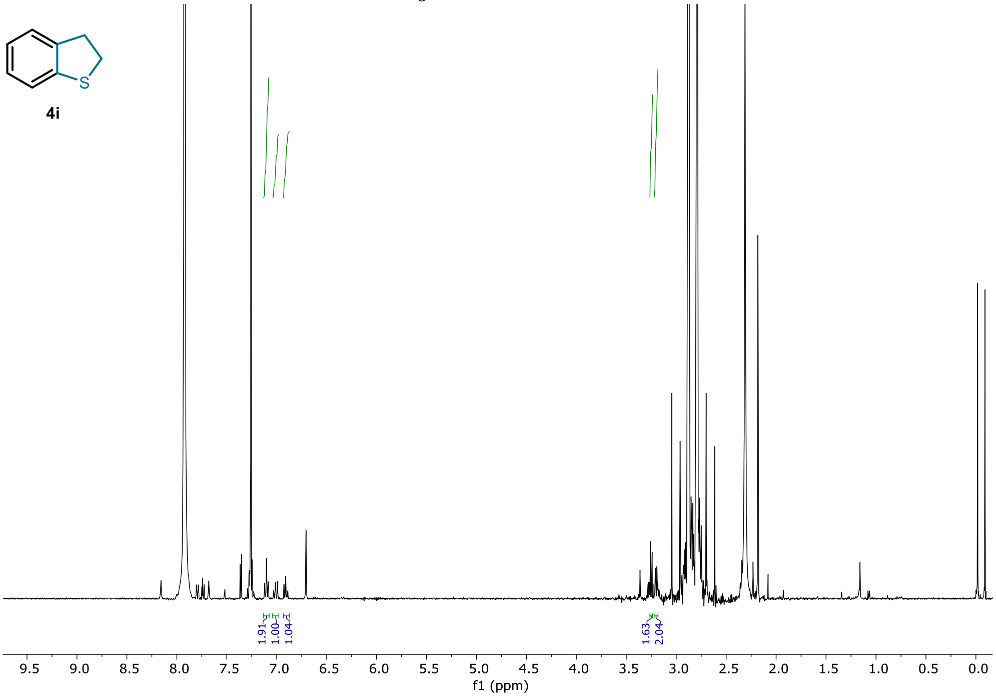

^{1}H NMR of the crude mixture in $CDCl_3$

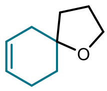

5e

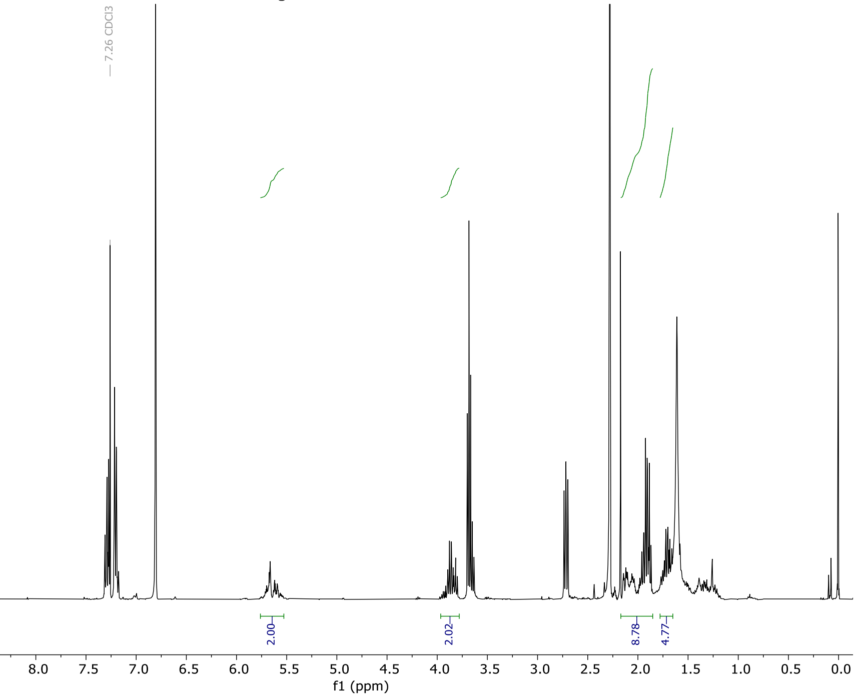

^{1}H NMR (600 MHz, $CDCl_3$)

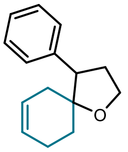

5z
diastereomer mixture 1:0.5

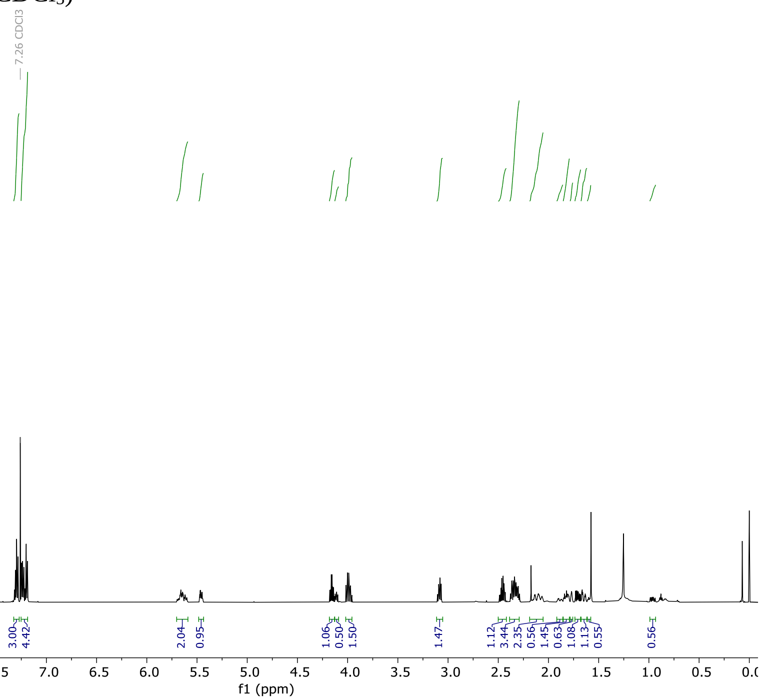

^{13}C NMR (151 MHz, $CDCl_3$)

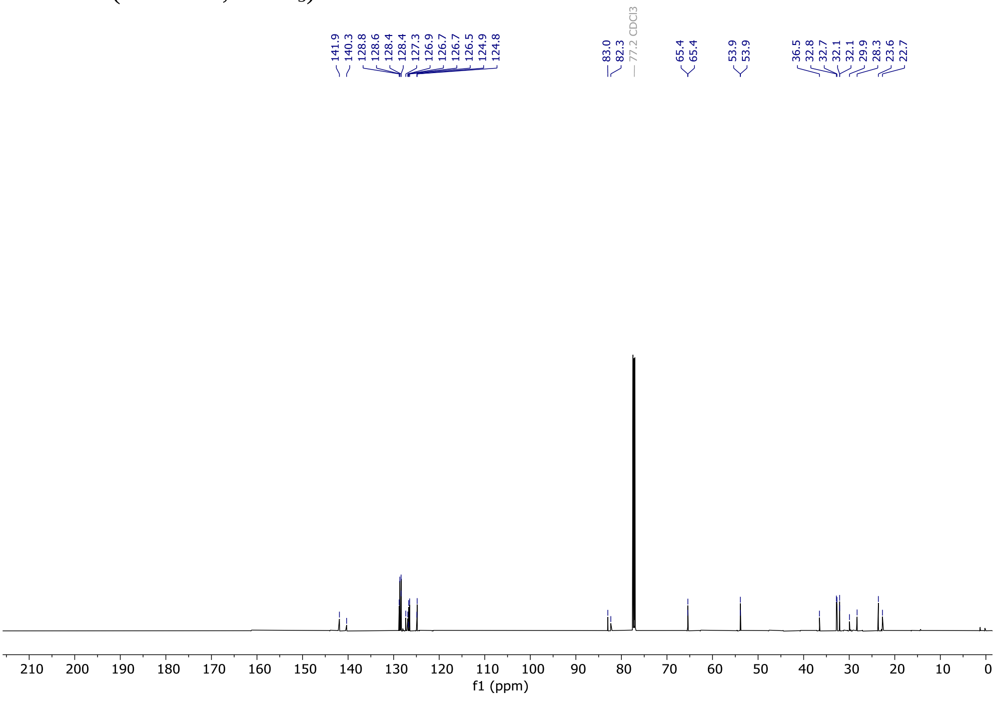

^{1}H NMR (600 MHz, $CDCl_3$)

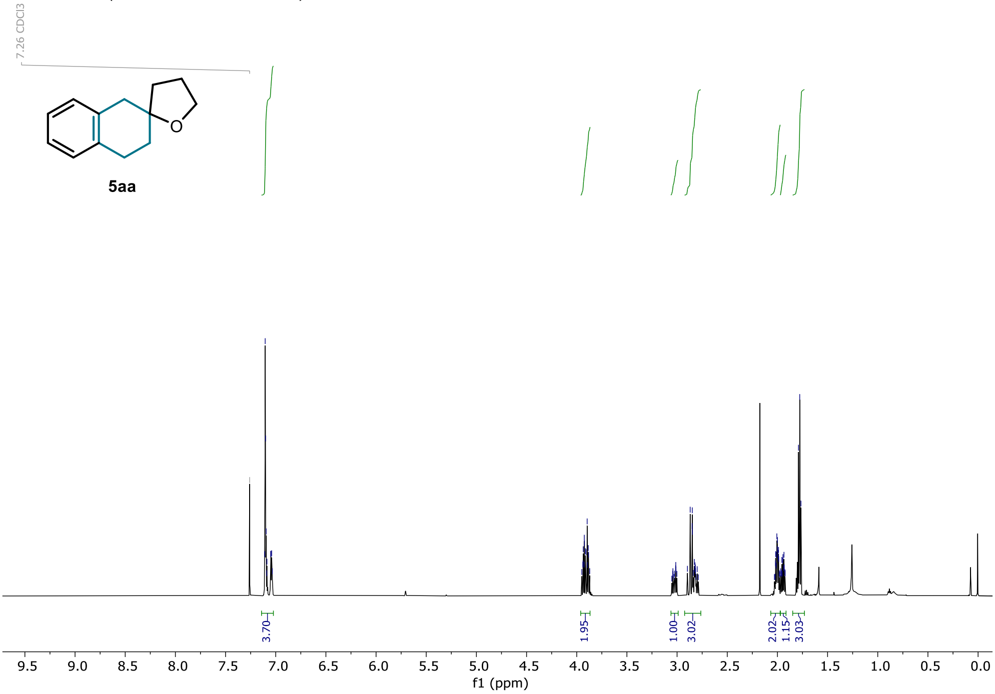

^{13}C NMR (151 MHz, $CDCl_3$)

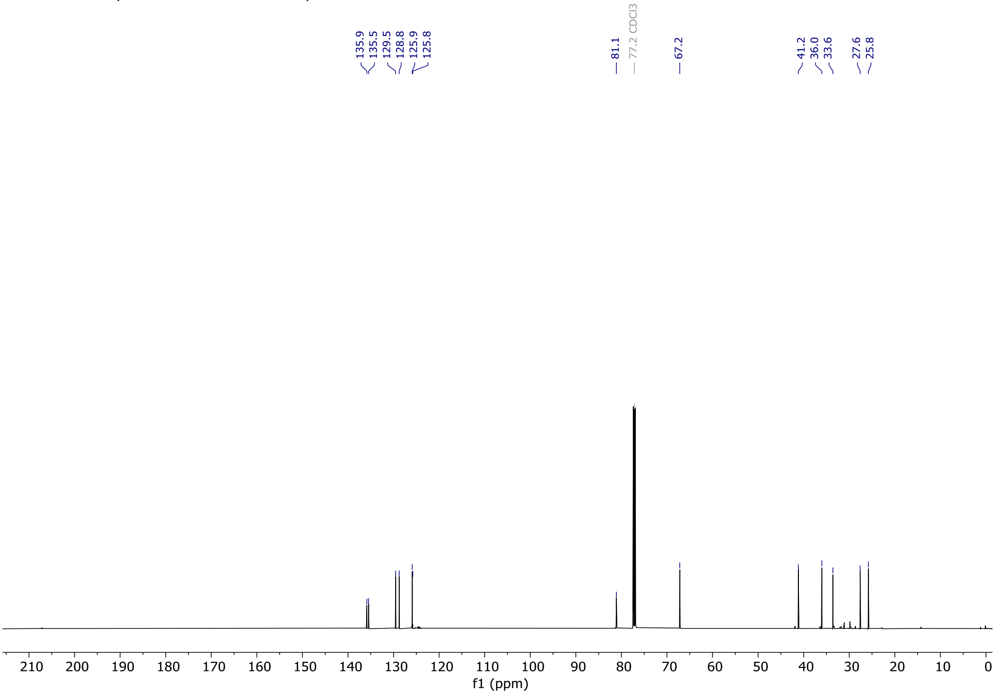

^{1}H NMR (600 MHz, $CDCl_3$)

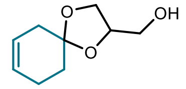

5y
diastereomer mixture 1:1

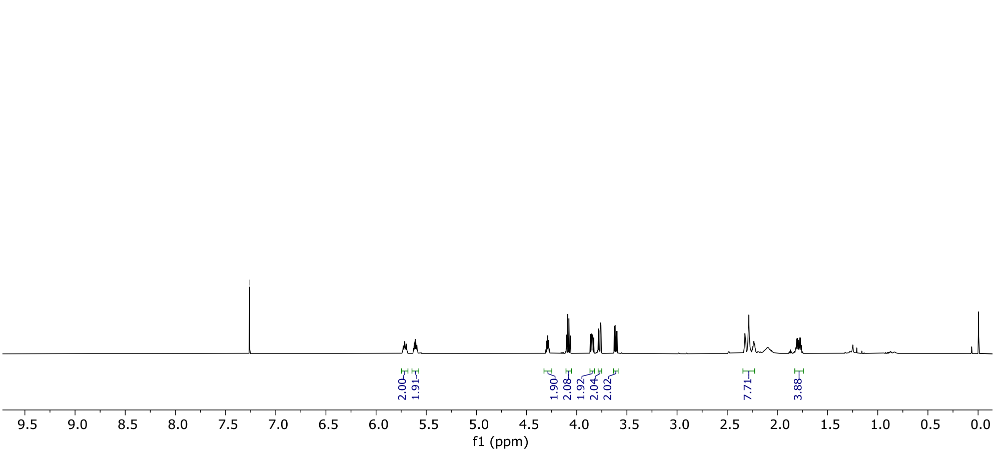

^{13}C NMR (151 MHz, $CDCl_3$)

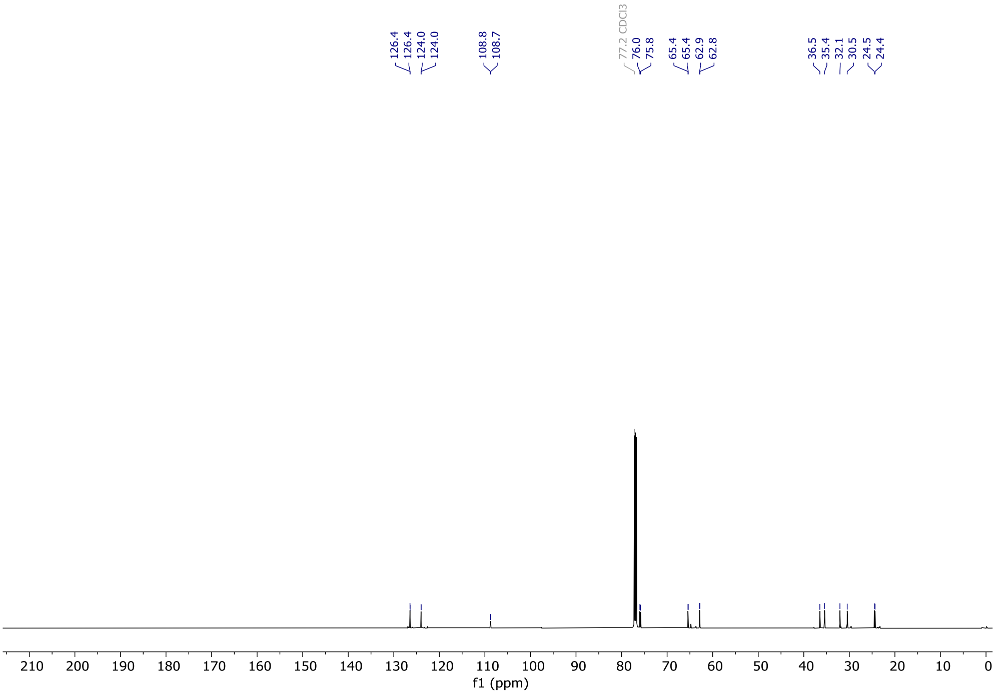

^{1}H NMR (600 MHz, $CDCl_3$)

5ab

diastereomer mixture 1:1

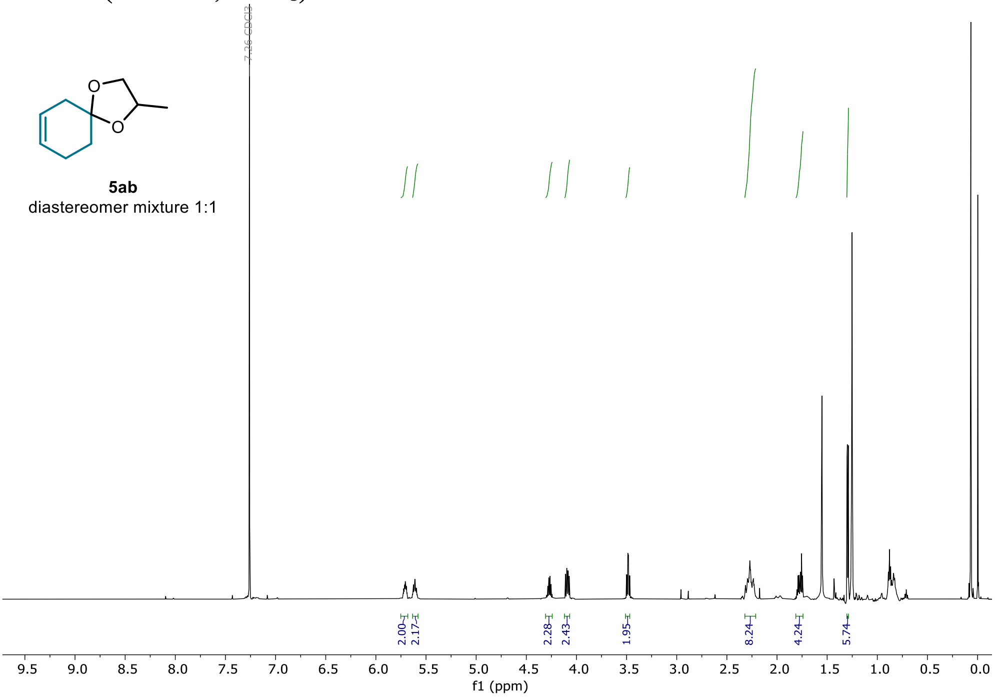

^{13}C NMR (151 MHz, $CDCl_3$)

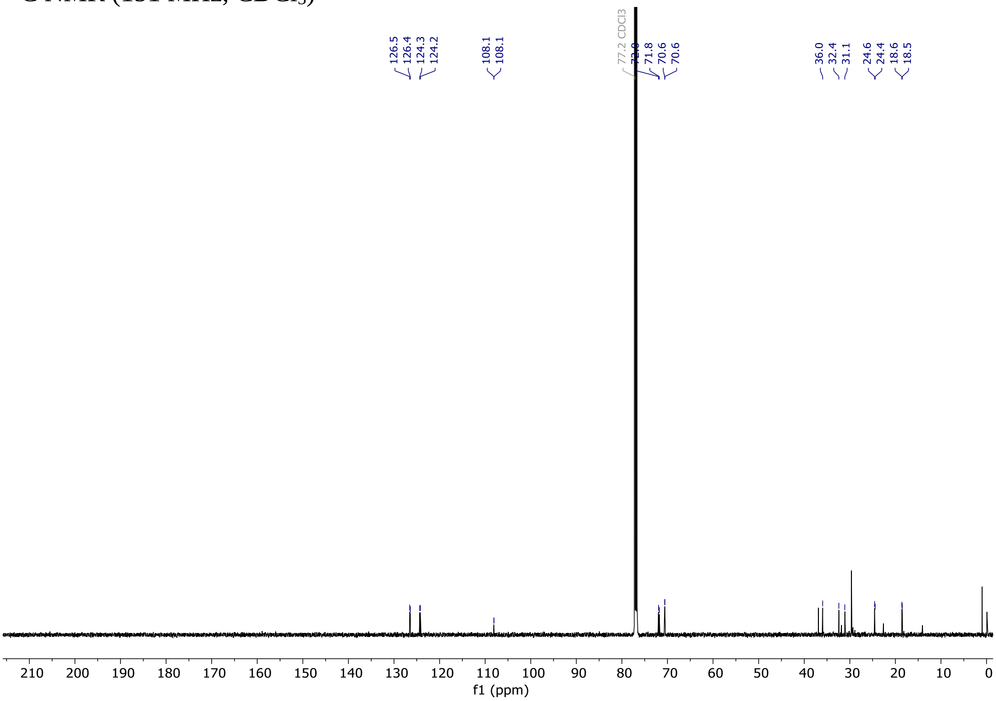

^{1}H NMR (600 MHz, $CDCl_3$)

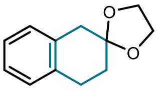

5s

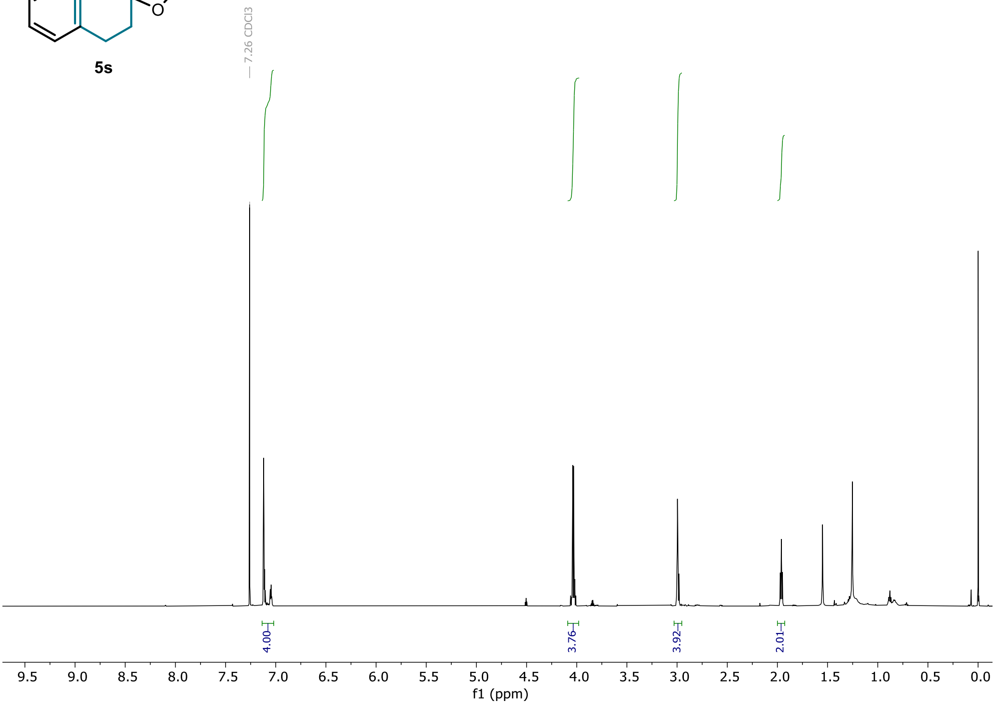

^{13}C NMR (151 MHz, $CDCl_3$)

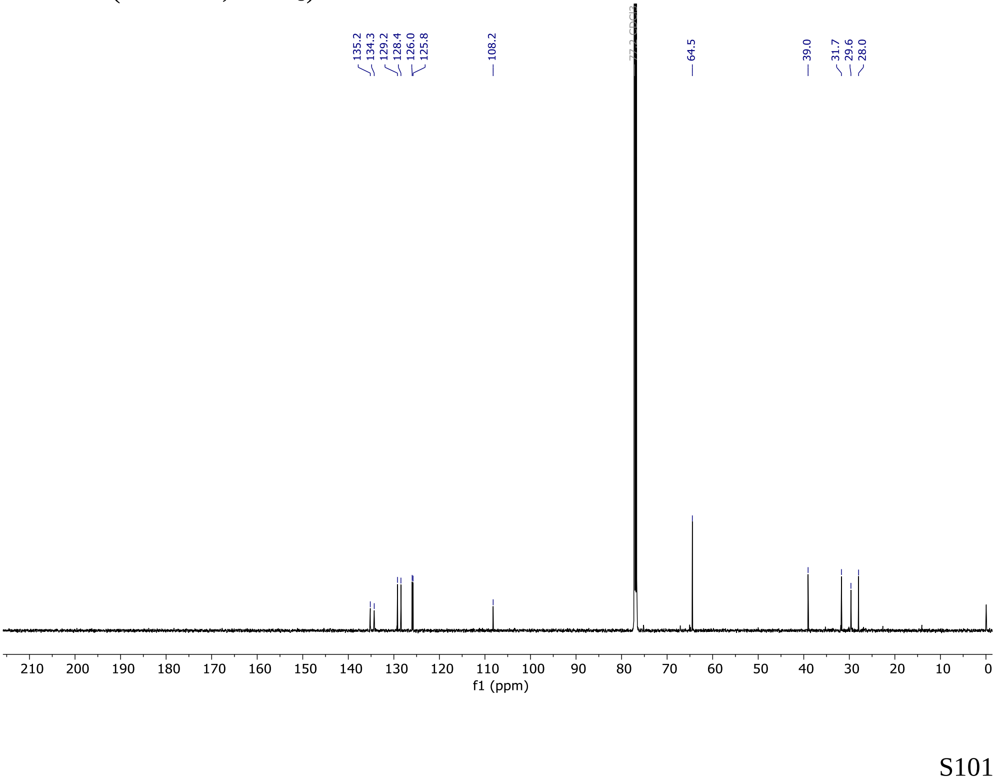

^{1}H NMR of the crude mixture in $CDCl_3$

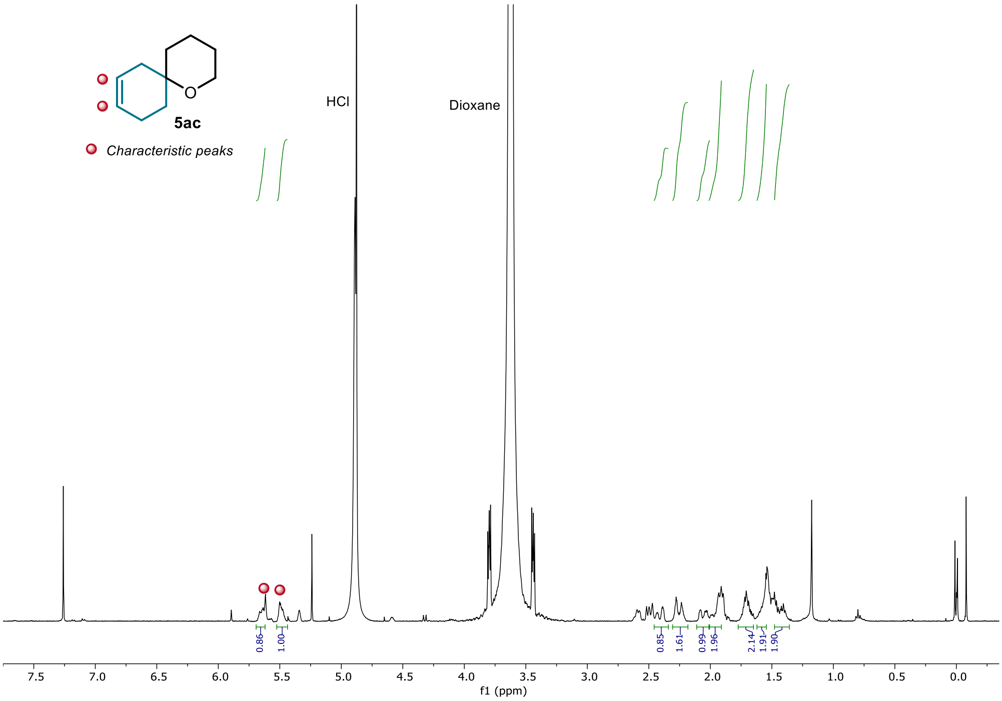

^{13}C NMR of the crude mixture in $CDCl_3$

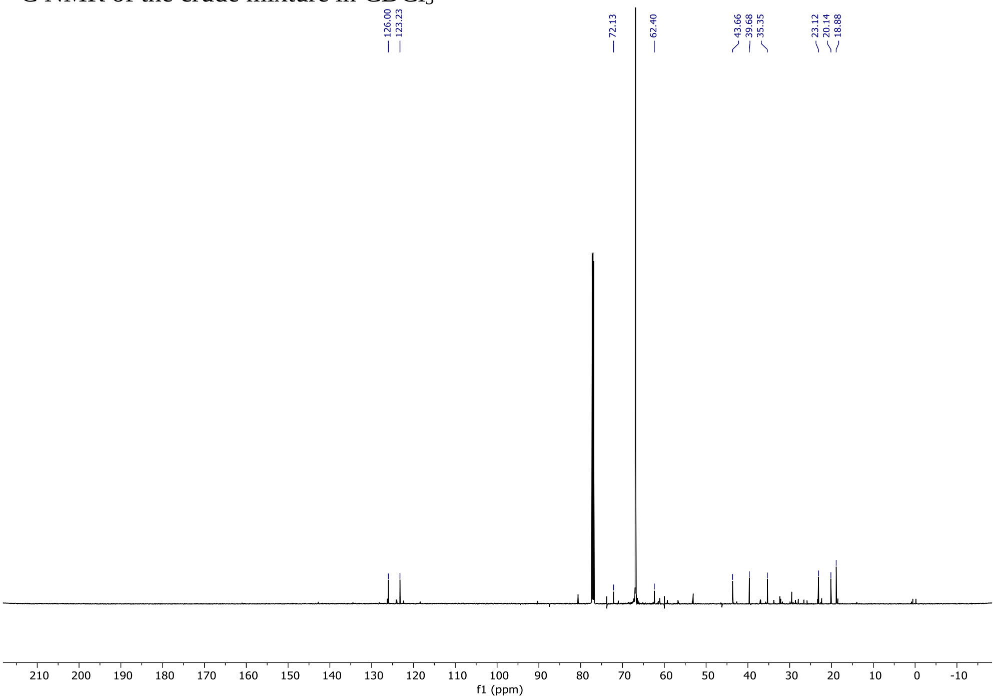

^1H NMR of the crude mixture in $CDCl_3$

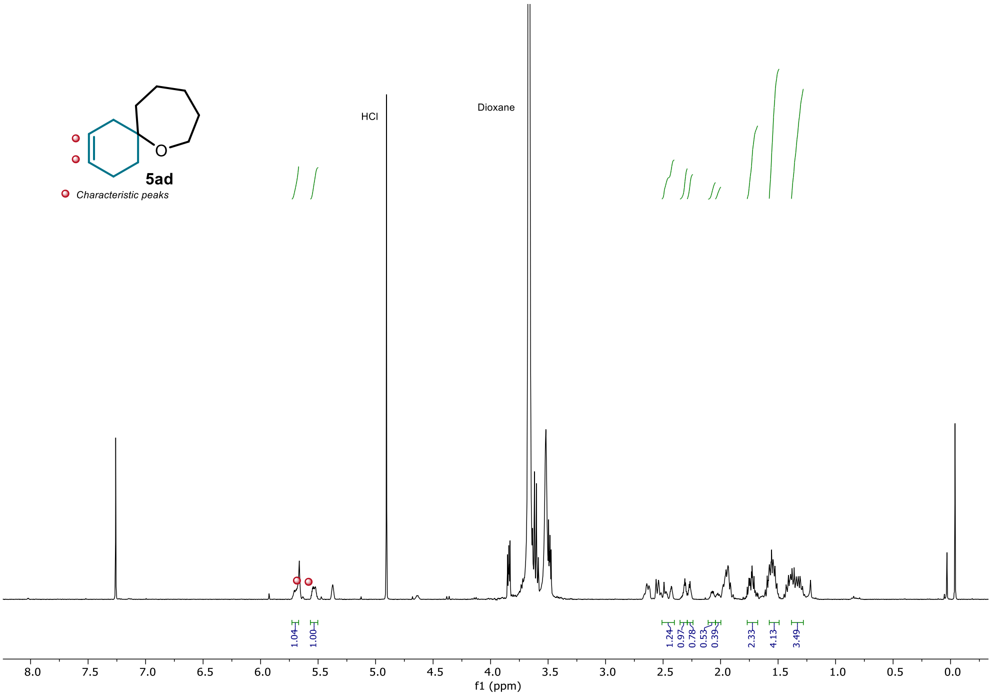

^{13}C NMR of the crude mixture in $CDCl_3$

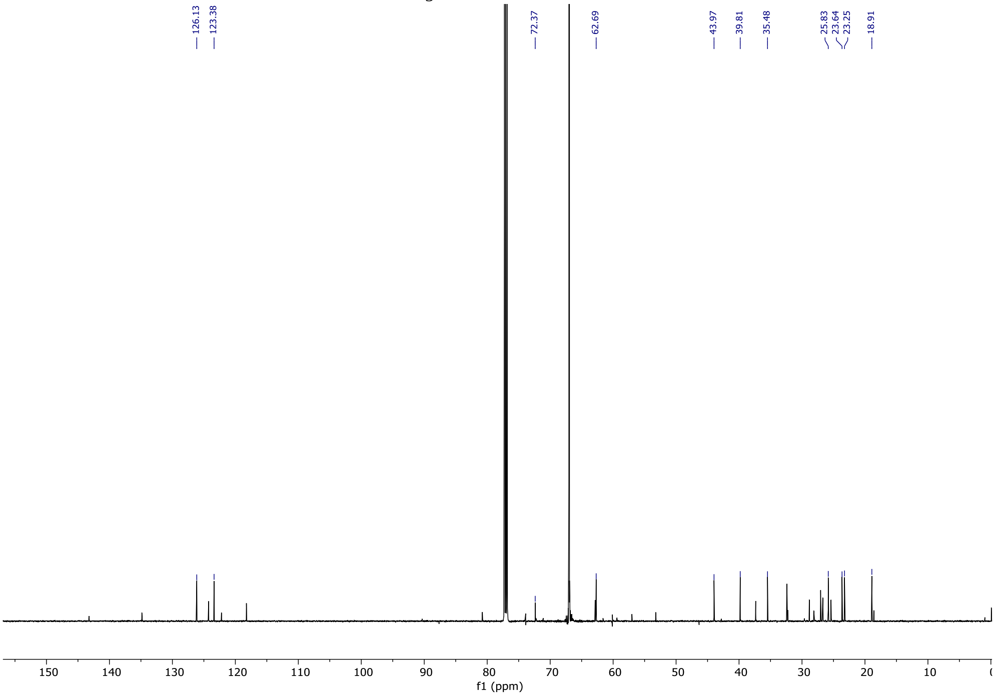

^{1}H NMR (600 MHz, $CDCl_3$)

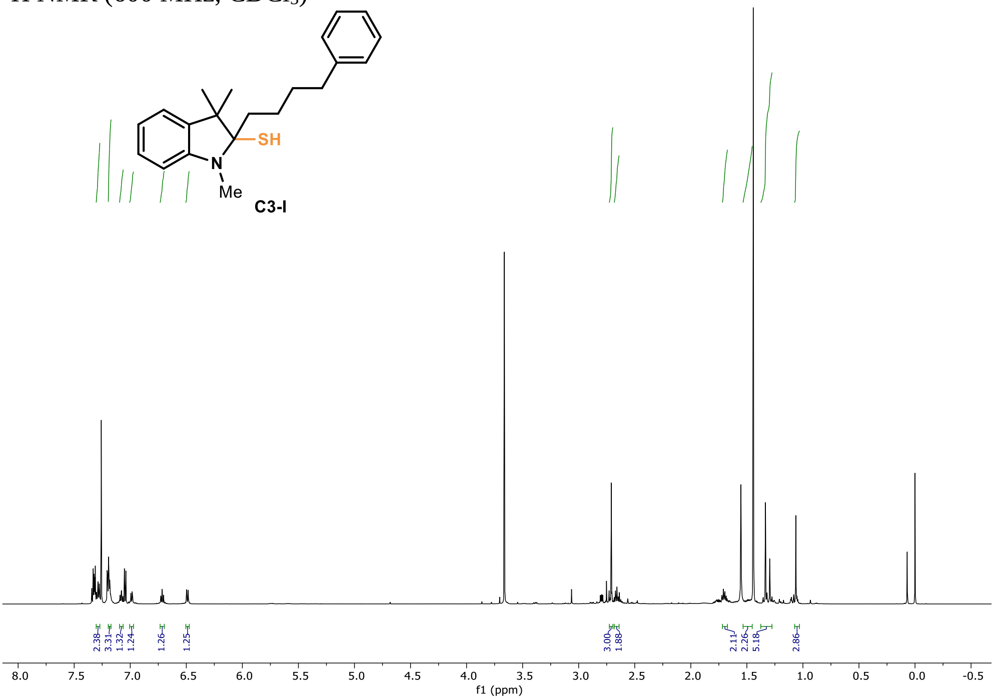

^{13}C NMR (151 MHz, $CDCl_3$)

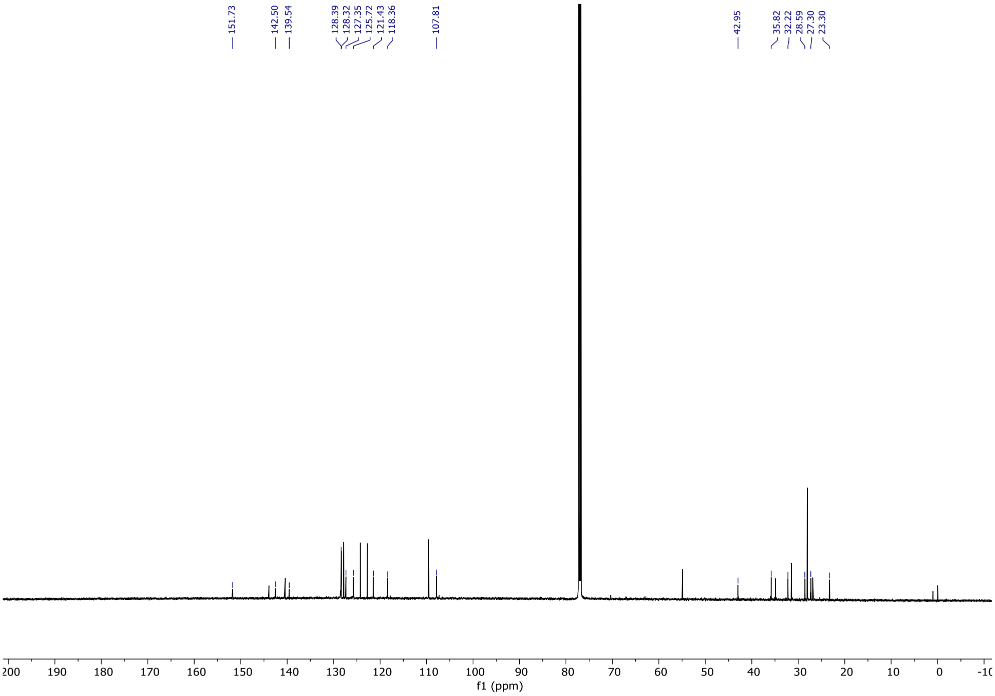

Supplement: Supplementary file 1 [file ja5c17373_si_001.pdf]
